# The mitochondrially-localized nucleoside diphosphate kinase D (NME4) is a novel metastasis suppressor

**DOI:** 10.1186/s12915-021-01155-5

**Published:** 2021-10-21

**Authors:** Marie-Lise Lacombe, Frederic Lamarche, Olivier De Wever, Teresita Padilla-Benavides, Alyssa Carlson, Imran Khan, Anda Huna, Sophie Vacher, Claire Calmel, Céline Desbourdes, Cécile Cottet-Rousselle, Isabelle Hininger-Favier, Stéphane Attia, Béatrice Nawrocki-Raby, Joël Raingeaud, Christelle Machon, Jérôme Guitton, Morgane Le Gall, Guilhem Clary, Cedric Broussard, Philippe Chafey, Patrice Thérond, David Bernard, Eric Fontaine, Malgorzata Tokarska-Schlattner, Patricia Steeg, Ivan Bièche, Uwe Schlattner, Mathieu Boissan

**Affiliations:** 1 Sorbonne Université, Inserm, Centre de Recherche Saint-Antoine, CRSA, Paris, France; 2 Université Grenoble Alpes, INSERM U1055, Laboratory of Fundamental and Applied Bioenergetics (LBFA), and SFR Environmental and Systems Biology (BEeSy), Grenoble, France; 3 Laboratory of Experimental Cancer Research, Department of Human Structure and Repair, Cancer Research Institute Ghent (CRIG), Ghent University, Ghent, Belgium; 4 Molecular Biology and Biochemistry Department, Wesleyan University, Middletown, USA; 5 Women’s Malignancies Branch, Center for Cancer Research, National Cancer Institute, Bethesda, USA; 6 Cancer Research Center of Lyon, INSERM U1052, CNRS UMR 5286, Léon Bérard Center, Lyon University, Lyon, France; 7 Unit of Pharmacogenetics, Department of Genetics, Curie Institute, Paris, France; 8 Reims Champagne Ardenne University, INSERM, P3Cell UMR-S 1250, SFR CAP-SANTE, Reims, France; 9 INSERM U1279, Gustave Roussy Institute, Villejuif, France; 10 Proteomics Platform 3P5, Paris University, Cochin Institute, INSERM, U1016, CNRS, UMR8104, Paris, France; 11 AP-HP, CHU Bicêtre, Laboratory of Biochemistry, Le Kremlin-Bicêtre Hospital, Le Kremlin-Bicêtre, France; 12 EA7537, Paris Saclay University, Châtenay-Malabry, France; 13 Université Grenoble Alpes, INSERM U1055, Laboratory of Fundamental and Applied Bioenergetics (LBFA), Institut Universitaire de France (IUF), Grenoble, France; 14 AP-HP, Laboratory of Biochemistry and Hormonology, Tenon Hospital, Paris, France

**Keywords:** Mitochondrial dynamics, Invasion, Metastasis, Nucleoside diphosphate kinase, NME4, Metabolic reprogramming, Prognosis biomarker, Retrograde signaling

## Abstract

**Background:**

Mitochondrial nucleoside diphosphate kinase (NDPK-D, NME4, NM23-H4) is a multifunctional enzyme mainly localized in the intermembrane space, bound to the inner membrane.

**Results:**

We constructed loss-of-function mutants of NDPK-D, lacking either NDP kinase activity or membrane interaction and expressed mutants or wild-type protein in cancer cells. In a complementary approach, we performed depletion of NDPK-D by RNA interference. Both loss-of-function mutations and NDPK-D depletion promoted epithelial-mesenchymal transition and increased migratory and invasive potential. Immunocompromised mice developed more metastases when injected with cells expressing mutant NDPK-D as compared to wild-type. This metastatic reprogramming is a consequence of mitochondrial alterations, including fragmentation and loss of mitochondria, a metabolic switch from respiration to glycolysis, increased ROS generation, and further metabolic changes in mitochondria, all of which can trigger pro-metastatic protein expression and signaling cascades. In human cancer, *NME4* expression is negatively associated with markers of epithelial-mesenchymal transition and tumor aggressiveness and a good prognosis factor for beneficial clinical outcome.

**Conclusions:**

These data demonstrate *NME4* as a novel metastasis suppressor gene, the first localizing to mitochondria, pointing to a role of mitochondria in metastatic dissemination.

**Supplementary Information:**

The online version contains supplementary material available at 10.1186/s12915-021-01155-5.

## Background

Carcinomas, the most prevalent malignancies in humans, arise from normal epithelial tissues in a multistep progression from benign precursor lesions. Metastasis, the final step in malignancy, is the cause of death for more than 90% of cancer patients. Molecular mechanisms underlying metastasis have to be elucidated for accurate detection and treatment [1]. During metastatic disease, complex pathways involving the tumor cell and the microenvironment mediate tumor invasion at the primary site, survival and arrest in the bloodstream, extravasation, and colonization at a secondary site. The first step in the metastatic cascade, i.e., the breakdown of epithelial intercellular adhesion and the acquisition of an invasive program allows epithelial cancer cells to breach the basement membrane and to invade stromal type I fibrillar collagen. These events are referred as epithelial-mesenchymal transition (EMT) and are considered crucial events in malignancy yet they are poorly understood [2]. During EMT, epithelial cells loose some of their epithelial characteristics, including cell adhesion and cell polarity; cytoskeletal rearrangement occurs that ultimately leads to an increased motility and an invasive phenotype.

Metastasis suppressors are cancer genes that inhibit the metastasis program without preventing primary tumor formation. Direct targeting of the metastatic process is an ultimate goal in cancer therapy which among others requires a more complete understanding of metastasis suppressor genes and their cellular functions [3]. Due to the complex multistep mechanisms underlying the metastatic process, metastasis suppressors show a large variety of molecular functions and cellular locations, including cytosol, plasma membrane and nucleus [3, 4]. From all the metastasis suppressors that have been described so far, not one has been localized in mitochondria.

Here we report on the *NME4/NM23-H4* gene, encoding a nucleoside diphosphate kinase (NDPK) that is localized in mitochondria: NDPK-D/NME4 (further only called NDPK-D). It is a member of the multifunctional NDPK/NME protein family [5, 6], localized mainly in the mitochondrial intermembrane space, bound to the inner membrane by anionic phospholipids like cardiolipin (CL) [7–9]. At that location, NDPK-D has two crucial functions for mitochondrial physiology: (i) phosphotransfer from oxidatively generated ATP to different nucleoside diphosphates, mainly GDP, to generate the GTP for local fueling of mitochondrial GTPases like Optic Atrophy 1 (OPA1), a driver of mitochondrial fusion at the mitochondrial inner membrane [10, 11] and (ii) CL transfer from the inner to the outer membrane, where it serves as a pro-mitophagic or pro-apoptotic signal [10, 12]. Interestingly, cytosolic/nuclear members of the NDPK/NME family are also metastasis suppressors, including the first of all and possibly best studied one, NDPK-A/NME1 [13], and possibly also NDPK-B/NME2 [14]. These NDPK isoforms act via their NDP kinase and histidine protein kinase activities on cell signaling, endocytosis and transcriptional regulation, finally affecting cell migration and proliferation [3, 15].

In this study, we separately invalidated the two NDPK-D activities, phosphotransfer and CL interaction/transfer, to analyze their effects on cell behavior. Cervical HeLa and breast MDA-MB-231 human tumor cells, which naturally express low levels of NDPK-D, were stably transfected with expression vectors, either empty or designed to express NDPK-D wild type or mutant proteins. Single point mutations were chosen to suppress either the catalytic NDPK activity of the enzyme or its ability to bind CL [9], which localizes the enzyme to the inner membrane and is essential for its function in CL intermembrane transfer. In a contrary experiment, we depleted NDPK-D by siRNA in the breast tumor cell line ZR75-1 which expresses a high level of NDPK-D protein. Strikingly, both mutations and NDPK-D depletion led to similar alterations in the cellular behavior, linked to altered mitochondrial structure and function, reprogramming of protein expression, and a morphotypic switch towards a pro-metastatic phenotype.

## Results

### NDPK-D mutations induce a morphotypic switch linked to a loss of intercellular adhesion

The HeLa clones that were analyzed here in detail have been used already in our earlier studies [9, 10, 12]. The control HeLa clones contain empty vector (abbreviated as CTR) and express very low levels of endogenous NDPK-D (Additional file 1: Fig. S1A). Clones stably transfected with vectors for different NDPK-D variants, namely wild-type (WT), CL-binding-deficient (BD; R90D mutation), or kinase-dead (KD; H151N mutation), express high levels of these NDPK-D proteins (Additional file 1: Fig. S1A). They present as a single strong band at the size of mature enzyme indicating a correct maturation of the NDPK-D variants (Additional file 1: Fig. S1A). In addition, in the case of WT and BD clones, also high NDP kinase enzyme activity was detectable in mitochondria while the activity of the catalytically inactive mutant was barely detectable (Additional file 1: Fig. S1B). Since the protein precursor is inactive [8], this further indicates correct mitochondrial import and processing of the pre-proteins. Exclusive localization of all NDPK-D proteins within mitochondria, and their absence in the cytosol, was validated by immunocytochemistry (Additional file 1: Fig. S1C). This confirms our earlier data with overexpression of GFP-fused protein in HEK293, subcellular fractionation [8, 9], and immunocytochemical localization of these NDPK-D variants in HeLa [9, 10, 12]. All this demonstrates correct processing and mitochondrial import of NDPK-D variants. Of note NDPK-A (NME1) and NDPK-B (NME2) protein levels remained unchanged in HeLa clones (Additional file 1: Fig. S1D).

**Fig. 1:**
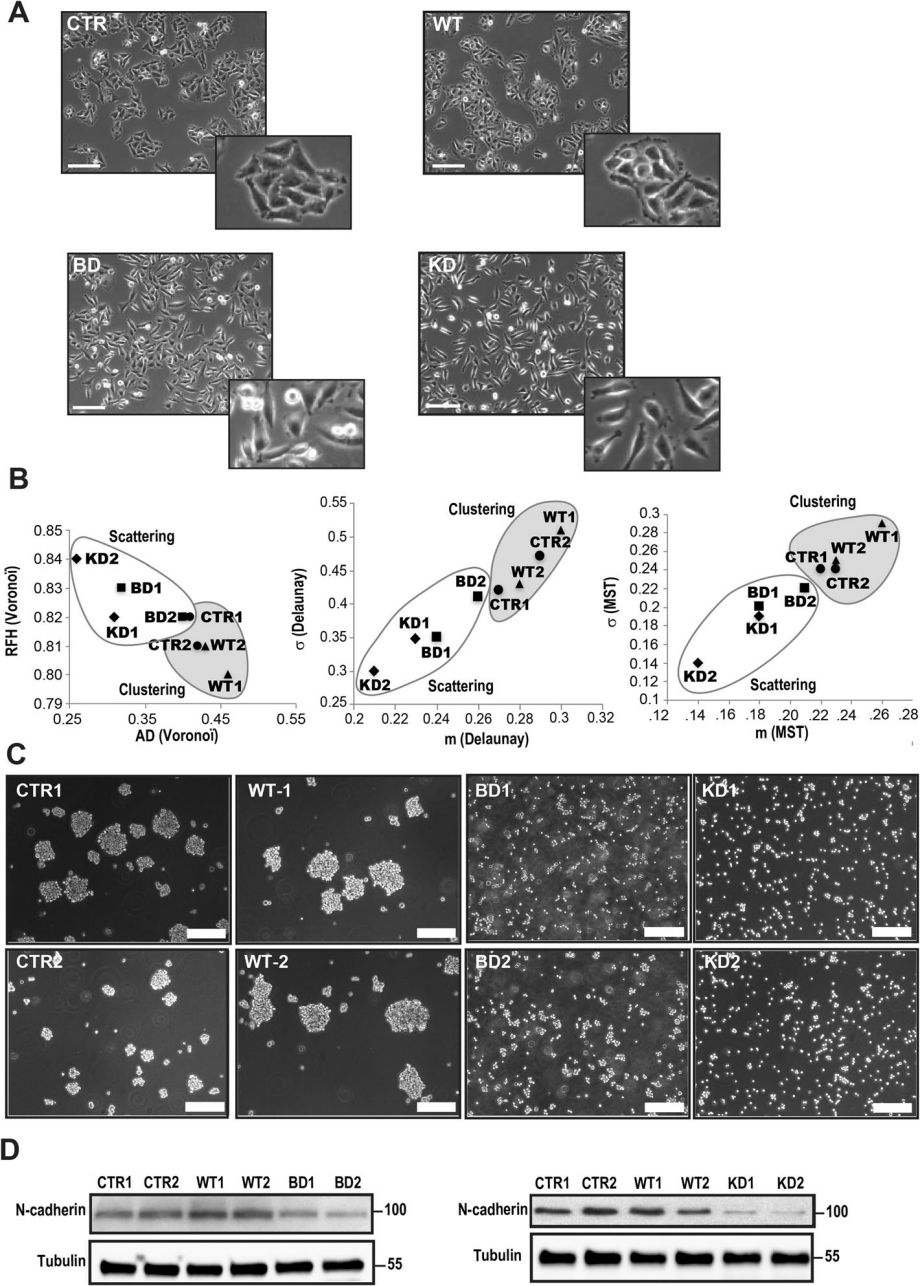
Morphotype and aggregation/adhesion of HeLa clones. **A** Morphology assessed by phase-contrast microscopy. Scale bars: 100 μm. **B** Cellular sociology of the different NDPK D clones. Clone partitions and graphs were obtained by three different methods after 24 h of culture [16]. Two parameters were deduced from each method, namely AD (area disorder) and RFH (roundness factor homogeneity) for Voronoi’s partition, m (average length) and σ (standard deviation) for both Delaunay’s graph and the MST. **C** Slow aggregation assay, performed by seeding the HeLa clones on top of a gelified agar medium. Scale bars: 200 μm. **D** N-cadherin levels. Representative immunoblots of HeLa cell extracts run in duplicate. Abbr. of HeLa clones throughout the text according to the expressed NDPK-D: CTR, control/empty vector; WT, wild-type; BD, CL-binding-deficient mutant; KD, kinase-dead mutant. Where indicated, two independently isolated clones of the same type (e.g., CTR1, CTR2) were analyzed

The most obvious difference immediately observable between the HeLa clones were two distinct and very different types of cell cohesion and morphology (Fig.1). While controls and NDPK-D WT expressing cells were organized as epithelioid clusters, even more compact for the WT clone, cells expressing either of the two NDPK-D mutants, BD or KD, grew as randomly dispersed single cells, exhibiting none to very few cell-cell contacts, most pronounced for the KD mutant (Fig. 1A). Cell cohesion was further quantified in a cell dispersion assay, using algorithmic programs of cell sociology [16]. This analysis confirmed that expression of mutated NDPK-D resulted in a more random spatial distribution of the cells compared with controls and WT enzyme expressing cells (Fig. 1B).

The acquisition of a scattered cellular phenotype is often associated with alterations in intercellular adhesion. We therefore investigated the impact of NDPK-D mutations on the cell-cell adhesion properties of HeLa cells in a slow aggregation assay [17], where cells are grown on soft agar (Fig. 1C). Again, control and NDPK-D WT expressing clones formed compact aggregates, whereas both mutant clones did not.

Cell-cell adhesion of HeLa cells relies entirely on N-cadherin, since the well-known epithelial marker E-cadherin is not expressed [18]. N-cadherin was markedly decreased in both mutant NDPK-D expressing clones as compared to control and WT NDPK-D expressing cells, again most pronounced for the KD mutant (Fig. 1D). All these data consistently show a clear and similar morphotypic switch that occurred in the mutant NDPK-D expressing clones, with a marked loss of cell-cell aggregation and cell-cell adhesion.

### NDPK-D mutations increase 2D and 3D cell migration

**Fig. 2:**
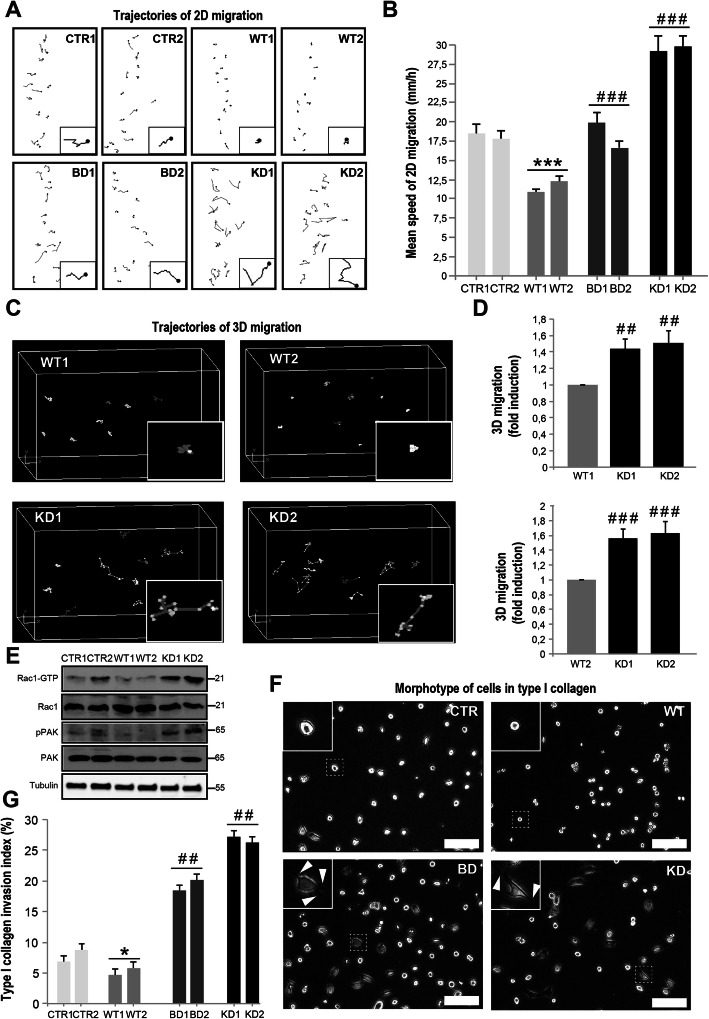
Migration and invasion properties of HeLa clones. **A**, **B** 2D migration assay with trajectories (**A**) and migration speed (**B**). The migration trajectories of cells were computed from images recorded every 15 min for 1 h. **C**, **D** 3D migration assay. Trajectories along the x-y-z plane (**C**) and migration speed (**D**) of the WT and KD clones. **E** Activation status of Rac1 (Rac1-GTP) and PAK (phosphorylated PAK, pPAK) of the CTR, WT, and KD clones as compared to total Rac1 and PAK protein. **F**, **G** Collagen invasion assay. The morphological aspect of cells in type I collagen (**F**; scale bars: 100 μm) with individual cells magnified (inserts) and arrowheads pointing to membrane protrusions, as well as invasion index (**G**). Note that mutations of NDPK-D result in the invasion of HeLa cells into native type I collagen gel. Data show means ± SEM (*n*=3). *p< 0.05, ***p< 0.005 relative to control/empty vector (CTR); ^##^*p*< 0.01 and ^###^*p*< 0.005 relative to wild-type (WT). For clone abbreviations see Fig. 1; where indicated, two clones of the same type were analyzed

To further examine consequences of altered cell morphology and cell-cell adhesion induced by NDPK-D mutants, we applied different migration assays (Fig. 2). In the 2D assay, trajectories of WT NDPK-D expressing clones were restricted and random, while a striking directional migration was observed with control and mutant expressing clones (Fig. 2A). Overexpression of WT NDPK-D significantly reduced the 2D migration speed as compared to controls and mutants (Fig. 2B). The 2D migration speed was significantly increased in KD as compared to WT expressing clones (Fig. 2B). The increased 2D migration speed exhibited by the mutant HeLa clones is also obvious through examination of video microscopy images (Additional file 2, 3, 4, 5: Movie 1-4). Similarly, the 3D migration assay revealed higher migration speed along the x-y-z planes for the two mutant expressing clones (KD1, KD2) as compared to the WT NDPK-D expressing clone (Fig. 2C, D). Since cell migration is largely mediated by Rho-GTPases, we evaluated Rac1 activation in these Hela clones via GST-pulldown assays (Fig. 2E). As compared to controls, overexpression of WT NDPK-D tended to reduce active, GTP-bound Rac1, while overexpression of KD NDPK-D strongly increased Rac1-GTP levels. Similar changes were observed for phosphorylation of p21-activated kinase PAK1, a downstream Rac1 effector. These data suggest that the Rac1 pathway participated in increased migration of this mutant.

### NDPK-D mutations increase the 3D invasive potential

Because the capacity to breach extracellular matrix barriers is critical for metastasis, we assessed whether expression of NDPK-D mutants affects the ability of HeLa cells to invade a three-dimensional matrix of native type I collagen during 24 h (Fig. 2F, G). HeLa cells are notoriously poor in degrading the extracellular matrix [19]. When seeded on native type I collagen, mutant NDPK-D formed numerous cellular protrusions (arrow heads in Fig. 2F), which invaded the collagen layer, while controls and WT enzyme expressing cells presented only few of these. Expression of both NDPK-D mutants strongly increased invasion through native type I collagen as compared to WT NDPK-D; the latter was even significantly lower as compared to the control (Fig. 2G). This is reminiscent to siRNA knock-down of cytosolic NDPK-A (NME1), a confirmed metastasis suppressor, which also generates a scattered (Additional file 6: Fig. S2A) and highly invasive phenotype (Additional file 6: Fig. S2B), reaching an invasion index of 20% through native type I collagen, similar to NDPK-D loss-of-function mutants. This indicates similar anti-invasive functions of NDPK-D (NME4) and NDPK-A (NME1) in HeLa cells. In addition, *NME1* silencing induced activation of the Rac1 signaling network, similar to NDPK-D loss-of-function mutants (Additional file 6: Fig. S2C). The invasive phenotype of mutant NDPK-D expression was further confirmed by a 14-day invasion assay (Additional file 7: Fig. S3). Here, sections of the collagen layer were examined 2-weeks after seeding the HeLa clones. While the WT clones remained on the surface, the KD clones deeply penetrated into the collagen layer. The invasive program of mutant clones was not related to an advantage in proliferation since their proliferation rates were lower than the one of the wild-type clones. This was also confirmed by protein levels of proliferation markers such as cyclin A, cyclin B1, and PCNA that were higher in WT clones than in CTR, BD, KD clones (Additional file 8: Fig. S4).

Selective pharmacological inhibition of pro-invasive pathways, including PI3K, Src, p38, JNK, and epidermal growth factor receptor (EGFR), strongly reduced invasion of a type I collagen matrix by both NDPK-D mutants (Additional file 9: Fig. S5A, B). Stimulation of EGFR and its downstream signaling (ERK, Akt, GSK3ß) by EGF was largely reduced in WT NDPK-D cells as compared to controls, while activation in NDPK-D mutants was comparable to controls or even higher (Additional file 9: Fig. S5C, D). Thus, strong responsiveness of mutant clones to EGF correlates with their reduced invasive potential upon EGFR inhibition.

### The cellular proteome reveals changes in metastasis-related and mitochondrial proteins

**Fig. 3:**
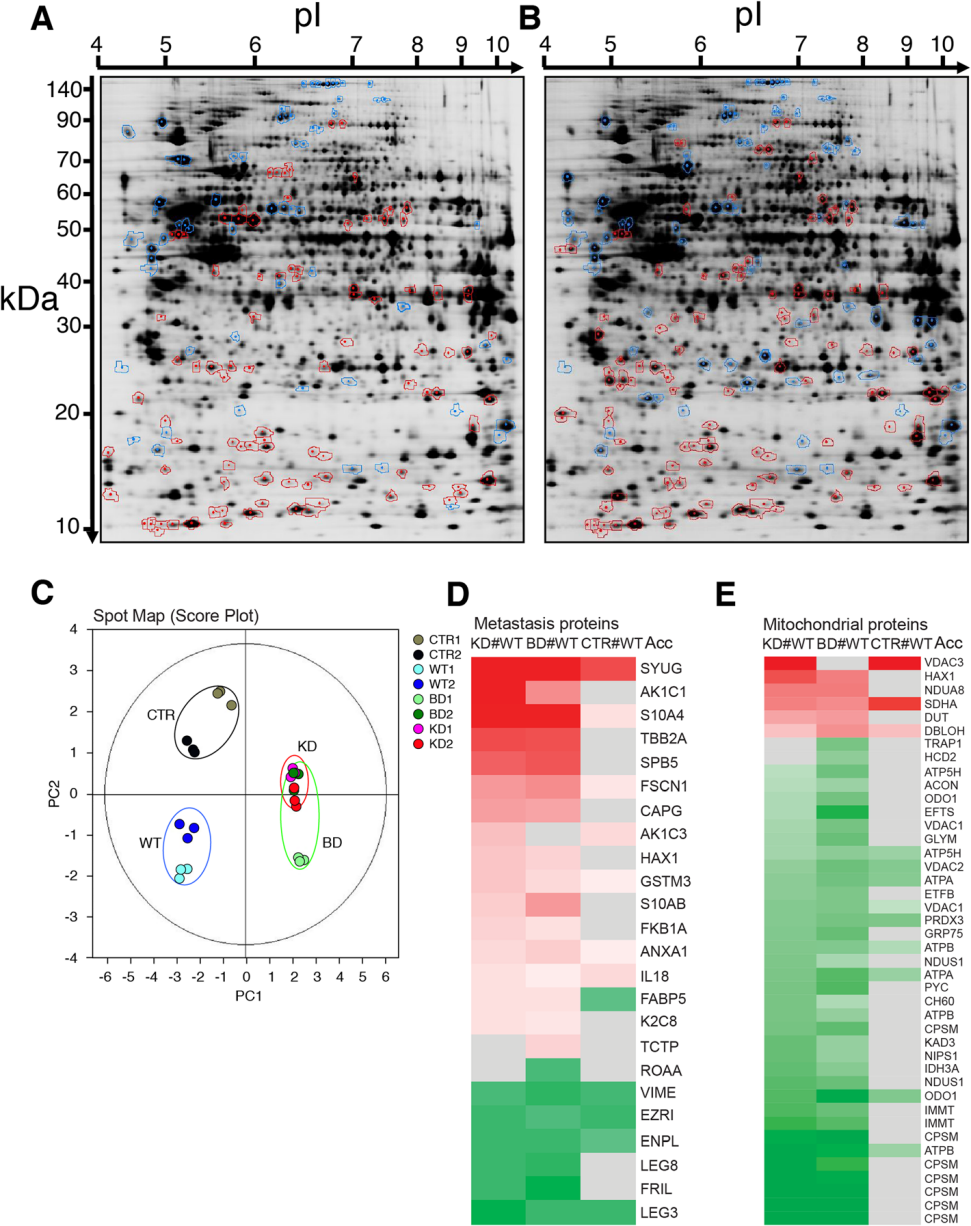
Cellular proteome of HeLa clones. **A**, **B** Two exemplary 2D gels showing identified differentially expressed protein spots, upregulated (circled in red) or downregulated (circled in blue) in mutant clones relative to WT. **A** KD mutant vs. WT: 157 spots differentially expressed, of which 94 spots upregulated and 63 spots downregulated. **B** BD mutant vs WT, 198 spots differentially expressed, of which 111 upregulated and 87 downregulated. **C** Principal component analysis (PCA) performed with all protein spots detected and matched. The score plot shows experimental maps; for more details see Material and Methods. **D**, **E** Heatmap clusters showing the relative changes of differentially expressed proteins within the two main identified functional groups related to metastasis (**D**) and mitochondrial dysfunction and oxidative stress (**E**); proteins were identified by IPA software, some mitochondrial proteins were further selected through annotation by UniProt. Color code for proteins: red, up-regulation; green, down-regulation; grey, no significant change; color saturation indicates the degree of change (see also Additional file 10: Table S1). For clone abbr. see Fig. 1; two clones of the same type (e.g., CTR1, CTR2) were analyzed in triplicate. In **D**, comparison of KD vs. WT yielded 22 proteins with altered expression (16 up- and 6 down-regulated). Comparison of BD vs. WT yielded 23 differentially expressed proteins (16 up- and 7 down-regulated), of which 21 are common with the previous one

The morphotypic switch and the scattered/migratory/invasive phenotype observed for Hela cells expressing NDPK-D mutants are striking features, considering that they are triggered by a single point mutation in a mitochondrial protein. This implies communication between molecular NDPK-D structure/function and cellular behavior in respect to cell adhesion, motility, and invasive potential. Since this should be mediated by changes in the cellular proteome, we next performed a comparative 2D-DIGE proteomic study with two independent clones of every experimental group (CTR, WT, BD, and KD NDPK-D). To identify significant changes within the differential expression pattern, multiple group-to-group comparisons were performed using the DeCyder biological variation analysis module (Fig. 3). A total of 206 differentially expressed protein spots were identified by mass spectrometry, corresponding to 157 different proteins (Additional file 10: Table S1; additional file 11: Table S2). Importantly, most changes in protein abundance relative to WT NDPK-D expressing cells occurred in the same sense for both mutants, KD (Fig. 3A) and BD (Fig. 3B). For quantitative analysis, principal component analysis was performed on the entire set of detected protein spots. Clearly, all samples of clones expressing mutant NDPK-D segregated from samples of WT NDPK-D expressing cells, while the control samples were different from both of the former groups (Fig. 3C; each group circled). Although the two clones analyzed for each of the four experimental groups also segregated to a certain degree, they remained quite close, emphasizing the good reproducibility of phenotypes and 2D-DIGE analysis. The proteomic profiles recapitulate the similarities seen for the scattered and migrative/invasive phenotypes. They suggest that both NDPK-D mutations trigger similar pathways to acquire these phenotypes, acting via a change in the cellular protein expression and/or degradation program.

Analysis of functionally related groups of proteins with IPA software identified more than twenty proteins involved in metastasis (Fig. 3D). The proteins are named according to their Entry Names in UniProt, see additional file 10: Table S1. They showed a change in expression level between WT *vs* control (CTR) and mutant (BD and KD) NDPK-D expressing clones. Of the overexpressed proteins involved in metastasis, fifteen were found in both KD and BD mutant clones; only one was unique to KD (AK1C3, abbreviated according to UniProt) and one to BD (TCTP). Among these proteins, the strongest overexpression was found for γ-synuclein (SYUG: + 7.8, + 10.8; all data given as fold-changes in KD and BD *vs*. WT). Further overexpressed proteins included actin-bundling protein Fascin (FSCN1: + 2.3, + 2.3), two calcium-binding S100 proteins (S10A4: + 3 to + 5; S10AB: about + 2), cytoskeletal tubulin β-2A (TBB2A: + 3.9, + 3.8), and interferon-stimulated gene 15 (ISG15: + 8.1, + 4.4). The latter, although not assigned to the metastatic pathway by IPA, was reported to promote invasion [20]. Of the downregulated proteins, again six were found in both mutant clones, and only one was unique to BD (ROAA). Overall, down-regulation was less marked. Of note, down-regulation of N-cadherin (Fig. 1D) failed to be identified by the proteomic analysis, probably due to its low pI (4.6) and high Mr (100 kDa). Immunoblotting analysis confirmed the 2D-DIGE results, e.g., overexpression of fascin, γ-synuclein, ISG15, S100A4 (S10A4), and tubulin β-2A in KD *vs*. WT (Additional file 12: Fig. S6A). At the mRNA level (Additional file 12: Fig. S6B-F), consistent with these changes in protein abundance, we observed strong up-regulation of ISG15, S100A4, and γ-synuclein. As expected, N-cadherin mRNA was downregulated in the KD clones as compared to WT. This suggests that these proteins are mainly regulated at the transcriptional level. Fascin mRNA levels were unchanged, indicating a different regulation. In conclusion, coordinated deregulation of multiple metastasis-related proteins in both NDPK-D mutant-expressing clones provides a molecular rationale for a role of NDPK-D in the metastatic process.

Another functional group identified by IPA was *Mitochondrial Dysfunction* and *Oxidative Stress* (Fig. 3E). Indeed, among proteins differentially expressed in mutant KD and BD clones *vs.* WT were many mitochondrial proteins. A marked change was downregulation of several core subunits of ATP synthase: alpha (ATPA: − 1.5, − 1.7), beta (ATPB: − 2.0, − 1.9), and delta (ATP5H: − 1.4, − 1.6), while few changes were detected in the respiratory chain. These concerned complex I, with a downregulation of the core subunit NADH-ubiquinone oxidoreductase 75 kDa (NDUS1, − 1.7, − 1.6) in the matrix-facing dehydrogenase module of the peripheral arm, and upregulation of the accessory subunit NADH dehydrogenase 1 alpha subcomplex subunit 8 (NDUA8, + 1.7, + 1.7), which faces the intermembrane space and is essential for complex I assembly [21, 22]. The most downregulated mitochondrial protein (> 2-fold) was carbamoylphosphate synthase 1 (CPSM), catalyzing the first committed step leading to arginine biosynthesis and urea cycle. This protein is represented by several spots resulting probably of maturation and/or posttranslational modifications [23]. Interestingly, there was also down-regulation of two other mitochondrial proteins that could potentially compensate for NDPK-D functions: adenylate kinase 3, a GTP: AMP phosphotransferase (KAD3: − 1.6, − 1.4) able to generate GTP and AMP from GDP and ADP and *vice versa*, and MICOS complex subunit MIC60 (IMMT: − 1.8, − 1.7). The latter complex bridges inner and outer mitochondrial membrane, similar to NDPK-D, and organizes cristae [24–26]. Finally, within the family of voltage-dependent anion channels (VDACs), controlling among others outer mitochondrial membrane permeability, an isoform switch occurred, with upregulated isoform 3 (VDAC3: + 2.4, + 1.3) and downregulated isoform 1 (VDAC1: − 1.5, − 1.5) and isoform 2 (VDAC2: − 1.4, − 1.6). Based on these data, and the phenotype of NDPK-D mutant expression described herein, we hypothesized (i) that there should be a primary effect of NDPK-D mutations on mitochondrial structure and/or function, and (ii) that this effect should be again similar for both mutants.

### NDPK-D mutations affect mitochondrial structure and function

**Fig. 4:**
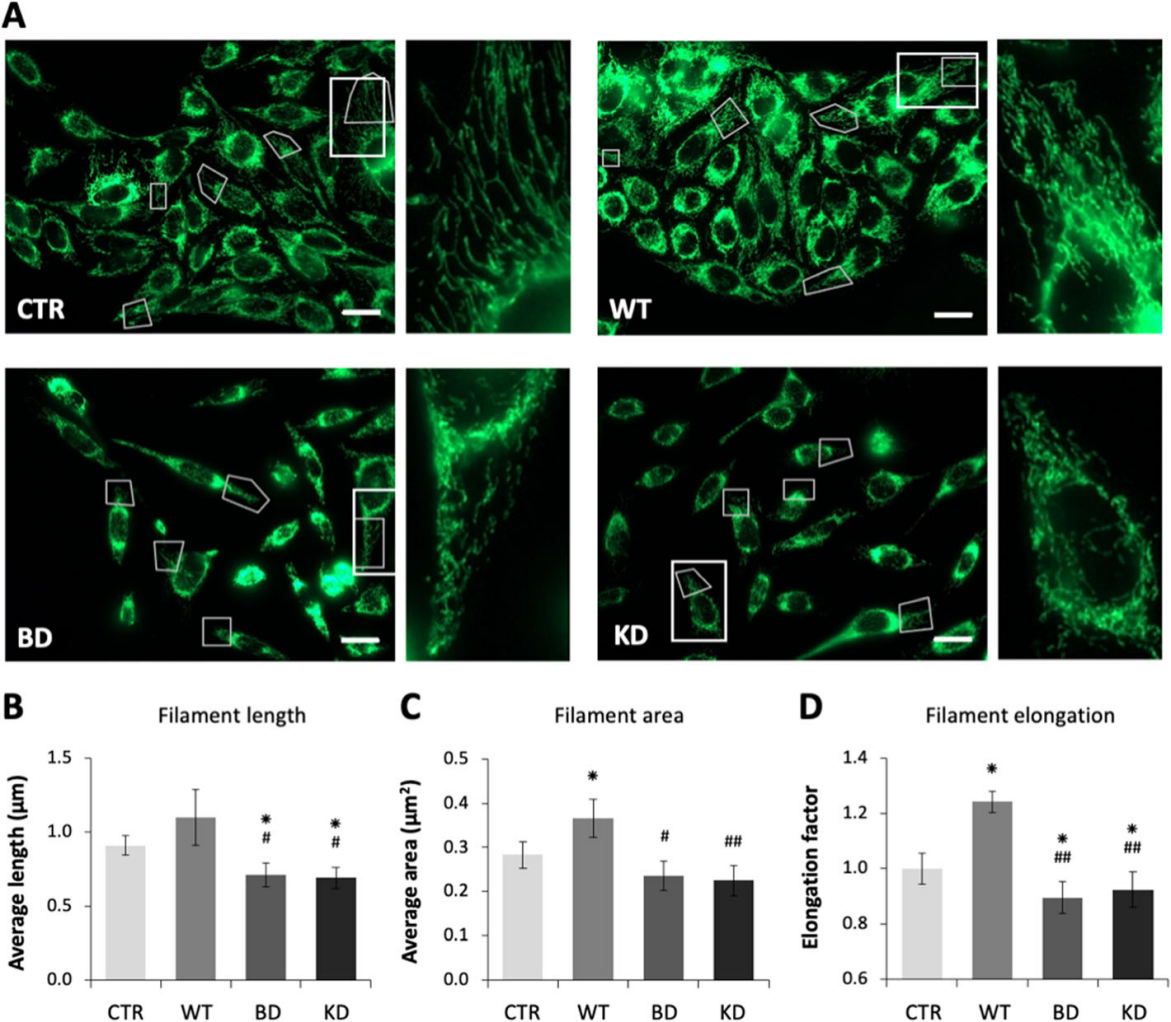
Mitochondrial network structure of HeLa clones. Network parameters determined in HeLa cells harboring empty vector control (CTR) or expressing wild-type (WT) or mutant NDPK-D (BD, KD), fixed and immunostained for mitochondrial MnSOD. **A** Representative confocal images show the regions of interests used for quantification (faint line boxes) and a representative region (bold line box) shown with 3.5-fold magnification to the right. Scale bar: 20 μm. **B** Average length of the mitochondrial filaments. **C** Average area of the mitochondrial filaments. **D** Elongation factor of the mitochondtrial filaments. All data are means ± SEM (*n*=5). *p< 0.05 relative to control/empty vector (CTR); ^#^*p*< 0.05 and ^##^*p*< 0.01 relative to wild-type (WT). For clone abbreviations, see Fig. 1

**Fig. 5:**
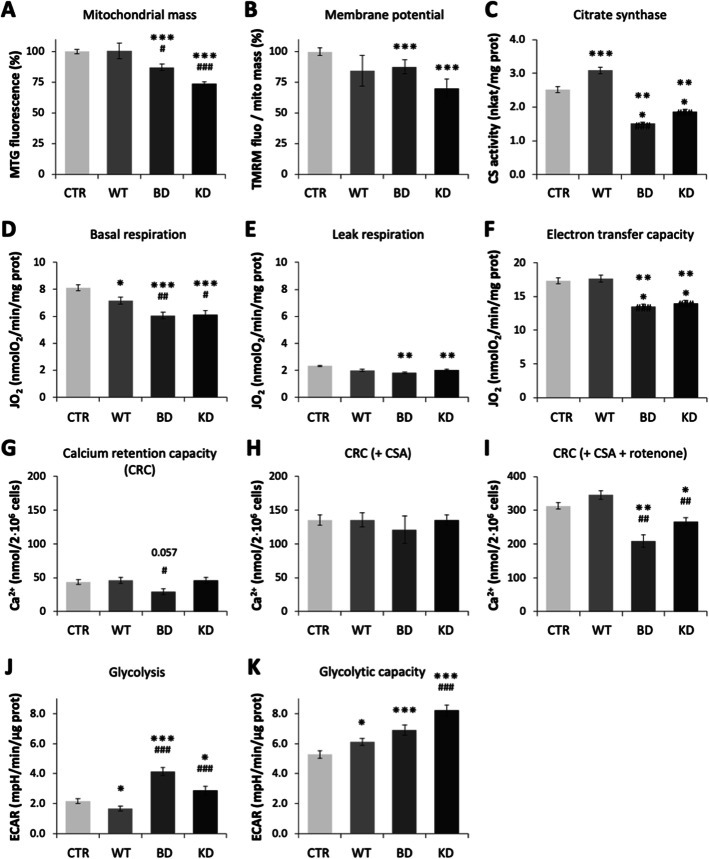
Mitochondrial and glycolytic functions of HeLa clones. HeLa cells harboring empty vector control (CTR) or expressing wild-type (WT) or mutant NDPK-D (BD, KD). **A** Mitochondrial mass determined with Mitotracker Green (MTG)-loaded cells; data are means ± SEM (*n*=18). **B** Mitochondrial membrane potential determined with TMRM loaded cells as difference before and after uncoupling with CCCP; data are means ± SEM (*n*=12). **C** Activity of Krebs cycle enzyme citrate synthase (CS); data are means ± SEM (*n*=7). **D**–**F** Respiration of intact cells (succinate as substrate) determined by oxygraphy; data are means ± SEM (*n*=12): **D** basal respiration in presence of glucose, **E** leak respiration after ATP synthase inhibition with oligomycin, **F** electron transfer capacity after uncoupling with CCCP. **G**–**I** Maximal calcium retention capacity (CRC) of permeabilized HeLa cells (succinate as substrate) before permeability transition occurs; data are means ± SEM (*n*=3): **G** without inhibitors, **H** with cyclosporin A (CSA), **I** with CSA and rotenone combined. **J**, **K** Extracellular acidification rate (ECAR) determined by Agilent Seahorse XF; data are means ± SEM (*n*=29): **J** basal ECAR, indicative for basal glycolysis, **K** maximal ECAR after inhibition of mitochondrial ATP synthase with oligomycin, indicative for glycolytic capacity. All data are from at least 3 different cultures. *p< 0.05, **p< 0.01, ***p< 0.005 relative to control/empty vector (CTR); ^#^*p*< 0.05, ^##^*p*< 0.01, and ^###^*p*< 0.005 relative to wild-type (WT). For clone abbreviations, see Fig. 1

Given its mitochondrial localization, we suspected that NDPK-D loss-of-function has primary effects on mitochondria. We first studied the mitochondrial network of HeLa cells, fixed and immunostained for the mitochondrial protein Mn-superoxide dismutase (MnSOD, Fig. 4A). Both NDPK-D mutant clones showed fragmentation of the network as compared to WT and control cells, determined by decreased filament length (Fig. 4B), area (Fig. 4C), and elongation (Fig. 4D). In contrast, the WT clone had higher elongation and surface area parameters as compared to controls (Fig. 4C, D). Thus, high levels of wild-type NDPK-D led to the most connected, filamentous mitochondrial network, while expression of NDPK-D mutants led to mitochondrial fragmentation, consistent with the key role of NDPK-D in fueling the mitochondrial fusion protein OPA1 [11]. Similar networks were observed with MitoTracker Green live stained live cells (Additional file 13: Fig. S7). Correlated with fragmentation, NDPK-D mutant clones also had lower mitochondrial mass as compared to WT and control cells, consistent with a preferential elimination of fragmented, smaller mitochondria (Fig. 5A).

We then determined basic functional parameters of mitochondria. The average mitochondrial membrane potential (ΔΨ_m_) decreased mainly in the NDPK-D mutant clones, giving first evidence for some mitochondrial dysfunction (Fig. 5B). Next, activity of the Krebs cycle enzyme citrate synthase (CS) increased with overexpression of WT NDPK-D, but decreased with the loss-of-function mutants as compared to controls (Fig. 5C). These changes cannot be explained by altered mitochondrial mass, thus indicating some rewiring of Krebs cycle activity in mutant *vs*. WT NDPK-D clones, consistent with a decreased abundance of the key Krebs cycle enzyme isocitrate dehydrogenase in mutant clones (IDH3A: − 1.6, − 1.5) in 2D-DIGE.

Respiratory parameters of intact cells were analyzed by oxygraphy. Basal respiration and total electron transfer capacity after uncoupling with CCCP (Fig. 5D–F [27]) were reduced in both mutant NDPK-D clones as compared to the WT NDPK-D clone and controls, reflecting reduced mitochondrial content (Fig. 5A). Leak respiration, i.e., basal uncoupling of mutant clones was less reduced, significant only relative to controls. Per mitochondrial mass, leak respiration even increased in the KD mutant (not shown), consistent with its decreased membrane potential. The capacity of mitochondria to accumulate calcium without opening the mitochondrial permeability transition pore (mtPTP) is another global readout of mitochondrial function (Fig. 5G–I). This calcium retention capacity, determined in digitonin permeabilized HeLa cells, was unchanged at baseline (except for the BD mutant) and with mtPTP inhibition by cyclosporine A (Fig. 5G, H). However, mtPTP inhibition by rotenone, an inhibitor of respiratory complex I [28, 29], alone (not shown) or in combination with cyclosporine A (Fig. 5I), was reduced in both mutant NDPK-D clones as compared to the WT and controls. The specificity of the effect for rotenone suggests altered mtPTP regulation at the level of complex I [29].

**Fig. 6:**
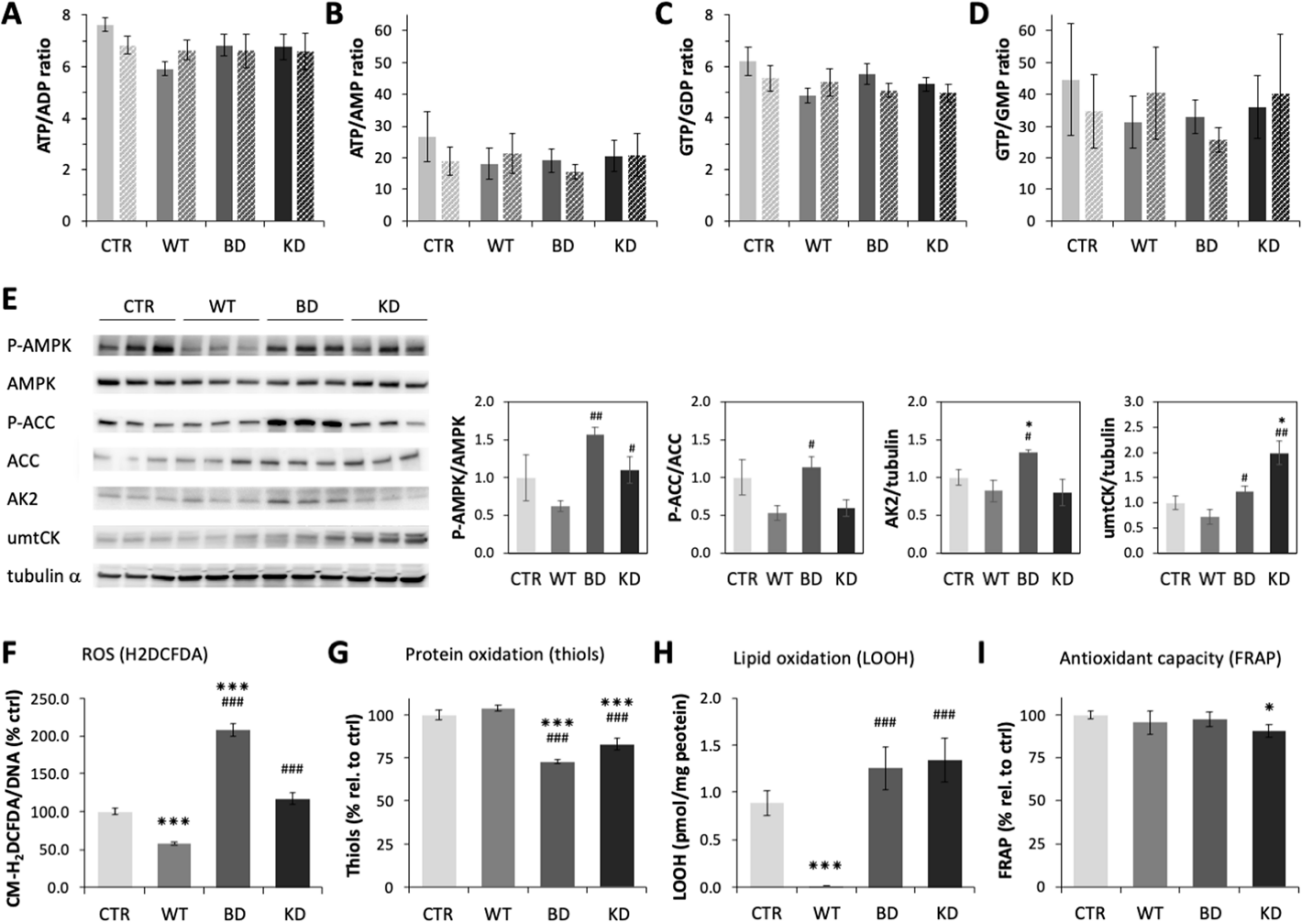
Energy-related kinases, nucleotides, and oxidative stress in HeLa clones. **A**–**D** Quantification of nucleotide ratios in HeLa cells (two clones of each condition, solid and hatched bars). **A** ATP/ADP ratio. **B** ATP/AMP ratio. **C** GTP/GDP ratio. **D** GTP/GMP ratio. **E** Expression of energy-related kinases in cell signaling and metabolism. Left: Representative immunoblots of cell extracts of the four HeLa clones for AMP-activated protein kinase (AMPK) and its activating phosphorylation at αT172 (P-AMPK), acetyl-CoA carboxylase (ACC), and its inhibiting phosphorylation at S79 (P-ACC), mitochondrial adenylate kinase isoform 2 (AK2), and mitochondrial ubiquitous creatine kinase (umtCK). Tubulin α served as loading control. Right: Quantification of band intensity ratios. Data given as means ± SEM (n=3), *p< 0.05, **p< 0.01 relative to CTR; #*p*< 0.05, #*p*< 0.01 relative to WT. **F**–**I** Quantification of oxidative stress markers determined in HeLa cells harboring empty vector control (CTR) or expressing WT or mutant NDPK-D (BD, KD). **F** Cellular levels of reactive oxygen species (ROS) determined with the fluorophore CM-H2DCFDA. Data are means ± SEM (*n*=3). **G** Reduced protein thiols (SH); their decrease indicates protein oxidation. Data are means ± SEM (*n*=3). **H** Lipid hydroperoxides. Data are means ± SEM (*n*=3). **I** Total antioxidant capacity determined by Ferric Reducing Ability of Plasma (FRAP), proportional to the reducing power of electron-donating antioxidants. Data are means ± SEM (*n*=6). *p< 0.05, ***p< 0.005 relative to control/empty vector (CTR); ^###^*p*< 0.005 relative to wild-type (WT). For clone abbreviations, see Fig. 1

The glycolytic activity of NDPK-D mutant clones at baseline and after inhibition of mitochondrial ATP synthesis as determined from the extracellular acidification rate were both increased as compared to the control and WT NDPK-D clones (Fig. 5 J, K). This is consistent with a compensatory metabolic switch from impaired respiratory ATP synthesis to increased glycolytic ATP generation in the NDPK-D mutant clones. We therefore investigated consequences of this metabolic switch on cell energetics. Based on a full quantification of adenine, guanine, cytosine, and uracil nucleotides (not shown), overall nucleotide equilibria like ATP/ADP and GTP/GDP ratios as well as ATP/AMP and GTP/GMP ratios were not significantly altered in NDPK-D mutants (Fig. 6A–D). However, induction of mild energy stress was apparent by activation or overexpression of kinases that are involved in cellular energy homeostasis (Fig. 6E). The energy sensor AMP-activated protein kinase (AMPK) was phosphorylated and activated in BD and KD clones relative to WT, also observed with phosphorylation of the AMPK substrate acetyl-CoA carboxylase in BD clones (Fig. 6E). The mitochondrial isoform of creatine kinase (umtCK) was upregulated in both BD and KD clones relative to WT, and the mitochondrial adenylate kinase AK2 was upregulated in the BD clone only (Fig. 6E). Upregulation of these kinases in the mitochondrial intermembrane space often occurs as a compensatory response under energy stress [30].

Finally, we were interested whether the observed mitochondrial dysfunctions would affect the cellular levels of reactive oxygen species (ROS) (Fig. 6F–I)). Indeed, increased ROS generation was observed in both mutants as compared with the WT expressing clone. The latter has a decreased ROS level as compared with control (Fig. 6F). This is in agreement with the measurement of markers of peroxidation. Oxidation of proteins (reduced thiols) was increased in mutants relative to control and wild-type (Fig. 6G). Lipid peroxides were barely detectable in wild-type expressing cells as compared with control. In both mutants, a significant increase in lipid peroxides was observed (Fig. 6H). The KD clone also had reduced antioxidant capacity (Fig. 6I).

### NDPK-D is a gatekeeper against EMT in breast cancer cells

To investigate the general relevance of NDPK-D for EMT, invasion, and metastasis, we turned to human breast cancer. We first analyzed *NME4* transcript levels by RTqPCR of a panel of human breast tumor cell lines according to their normal-like, hormone receptor (HR)-positive, and triple-negative (HR- and HER2-negative) status, where the HR-positive subtype has a more favorable prognosis than the triple-negative subtype (Additional file 14: Fig. S8). We observed significantly more *NME4* mRNA in the HR-positive human breast tumor cell lines than in the normal-like cell lines; these levels significantly decreased in the triple-negative human breast tumor cell lines, reaching a similar level to that observed in normal-like cell lines (Additional file 14: Fig. S8).

For genetic manipulation of functional NDPK-D levels in breast cancer, we chose two of these human breast tumor cell lines, MDA-MB-231 and ZR75-1. The former had the lowest level of *NME4* mRNA and was highly invasive and metastatic, while the latter had the highest level of *NME4* mRNA and was minimally invasive with an epithelial-like phenotype (Additional file 14: Fig. S8). We overexpressed WT and both BD and KD mutants of NDPK-D in MDA-MB-231 cells, similar to our approach with HeLa cells. The control MDA-MB-231 clones containing empty vector (CTR) expressed undetectable levels of endogenous NDPK-D (Additional file 15: Fig. S9A). Clones stably transfected with vectors for WT, BD, or KD NDPK-D expressed high levels of these proteins, presenting as a single strong band at the size of mature enzyme (Additional file 15: Fig. S9A). As shown for HeLa clones (Additional file 1: Fig S1C), MDA-MB-231 clones exhibited an immunostaining with anti-NDPK-D antibodies strictly colocalizing with mitochondria-selective marker (Additional file 15: Fig. S9B). With ZR75-1 cells, we did the contrary experiment, depleting NDPK-D specifically by expressing two different siRNAs. Western blotting confirmed the effective siRNA-mediated knockdown of NDPK-D (Additional file 16: Fig. S10)*.* We then investigated in both cell lines the functional consequences of such genetically modified NDPK-D expression on cell-cell adhesion, migration, and invasion properties.

**Fig. 7:**
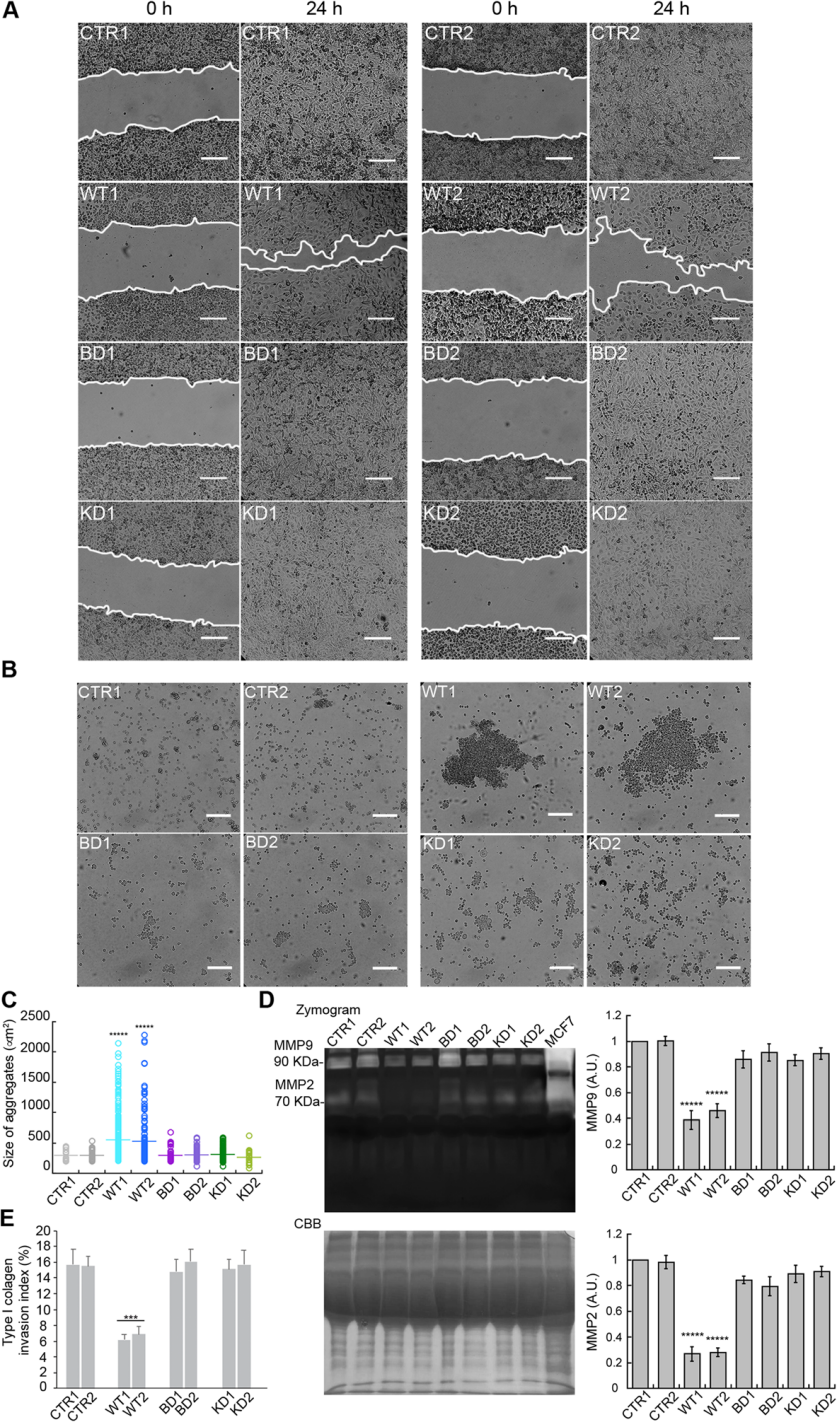
Migration, adhesion, and MMP activity of MDA-MB-231 cells genetically modified for NDPK-D. **A** Representative light microscopy images of MDA-MB-231 cell wound healing assay. Time 0 represents confluent monolayer wounds at 0 h and wounds were monitored 24 h after performing the scratch, in which empty vector control (CTR) monolayers became fully closed. Two different clones for each condition were studied. Images are representative of three independent biological replicates. Scale bar: 100 μm. **B** Representative light microscopy images of MDA-MB-231 dispase-based cell aggregation assay. Images are representative of three independent biological replicates; at least thirty pictures were analyzed for each replicate. Two different clones for each condition were studied. Scale bar 100 μm. **C** The size of the aggregates observed in B is depicted as the area of their horizontal projections. Data show means ± SEM of three independent biological replicates imaged. *****p< 0.00001 relative to control/empty vector (CTR). **D** Left panel, top, representative images of MMP activity by gelatin degradation zymography; the degradation bands of MMP9 and MMP2 are detected at 92 KDa and 72 KDa respectively. Left panel, bottom, representative Coomassie brilliant blue (CBB) of samples run simultaneously is shown as a loading control. Right panel, bar graphs represent the densitometric and statistical analyses of the bands obtained by gelatin zymography shown for MMP9 and MMP2 of four independent biological replicates. Concentrated culture media from MCF7 cells was used as positive control. Two different clones for each condition were studied. Data show means ± SEM (*n*=4). *****p< 0.00001 relative to control/empty vector (CTR). **E** Type I collagen invasion assay of MDA-MB-231 cells. Two different clones for each condition were studied. Data show means ± SEM. ***p< 0.001 relative to control/empty vector (CTR). Abbr. of MDA-MB-231 clones according to the expressed NDPK-D: CTR, control/empty vector; WT, wild-type; BD, CL-binding-deficient mutant; KD, kinase-dead mutant

**Fig. 8:**
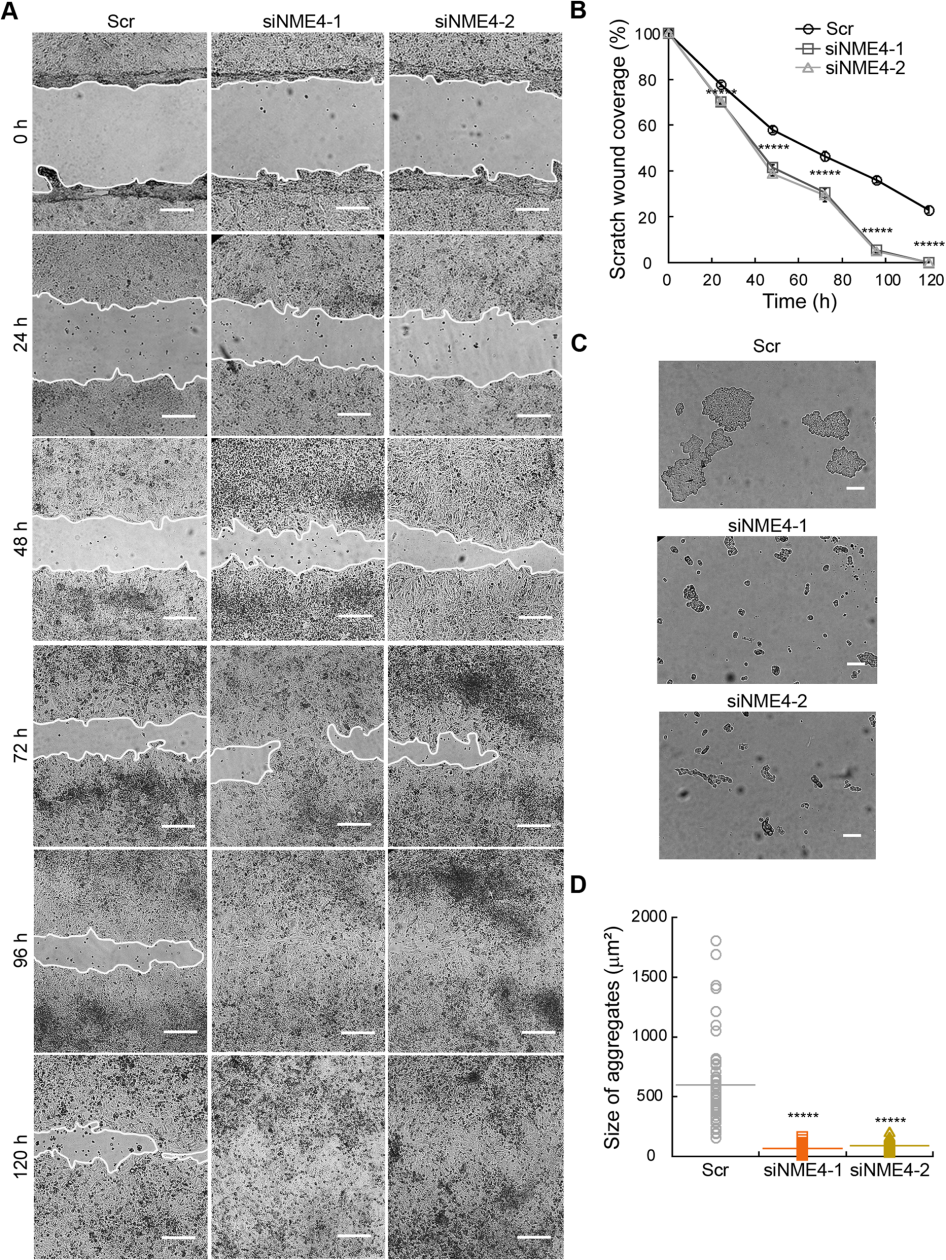
Migration and adhesion properties of ZR75-1 cells depleted for NDPK-D. **A** Representative light microscopy images of ZR75-1 cell wound healing assay. Time 0 represents confluent monolayer wounds at 0 h. Wounds were monitored for 120 h after performing the scratch, in which knockdown monolayers became fully closed. Two different siRNA targeting NME4 were used. Images are representative of three independent biological replicates. Scale bar 100 μm. **B** Quantification of the wound healing assay shown in A. Data show means ± SEM (*n*=3). *****p< 0.00001 relative to scramble control (Scr). **C)** Representative light microscopy images of ZR75-1 dispase-based cell aggregation assay. Images are representative of three independent biological replicates; at least fifty pictures were analyzed for each replicate. Two different siRNA targeting NME4 were used. Scale bar 50 μm. **D** The size of the aggregates observed in C is depicted as the area of their horizontal projections. Data show means ± SEM of three independent biological replicates imaged. *****p< 0.00001 relative to scramble control (Scr).

First, we applied a wound-healing assay, where a confluent cell monolayer is breached and the degree of migration to close the wound in a given time period is determined (Figs. 7A and 8A, B). In the case of MDA-MB-231 cells, two different clones for each condition were studied. When comparing the wounds immediately after the scratch (0 h) and 24 h later, control, BD and KD cells completely closed the wound, while WT cells were unable to do so, leaving 20–40% of the original scratch wound surface uncovered (Fig. 7A). In the contrary experiment with ZR75-1 cells, a model of slow growth breast carcinoma, wound closure was analyzed for 120 h after scratching (Fig. 8A, B). Here, cells depleted of NDPK-D migrated faster and covered nearly 100% of the scratch wound area at 96 h. Migration of control cells expressing NDPK-D was significantly slower than that of the knockdown cells and they were unable to close the wound at 96 h. We then analyzed a global readout of cell migration during wound healing, the secretion and activation of the metalloproteinases MMP2 and MMP9 (Fig. 7D, Additional file 17: Fig. S11A, B). Cell migration requires cyclic formation and destruction of focal adhesions and alteration of the composition of the extracellular matrix [31]. Migrating cells achieve this process by secreting Zn^2+^-dependent MMPs that respond to growth factors, cytokines, and hormones [32, 33]. The MDA-MB-231 clones overexpressing WT NDPK-D as compared to control clones showed a decrease by 60% and 80% in the gelatinase activity of MMP9 and MMP2, respectively (Fig. 7D), consistent with their impaired wound healing. No significant changes in MMP activity were detected in cells expressing BD or KD NDPK-D. In the contrary experiment with NDPK-D depletion in ZR75-1 cells, we found that NDPK-D depletion increased the secretion of MMP9 by 1.5-fold (Additional file 17: Fig. S11A, B), consistent with accelerated wound healing in this case. MMP2 activation was undetectable in these cells.

Accordingly, we assessed whether expression of NDPK-D variants affects the ability of MDA-MB-231 cells to invade a three-dimensional matrix of native type I collagen during 24 h (Fig. 7E). Expression of WT NDPK-D strongly reduced invasion through native type I collagen as compared to CTR; the latter had an invasive potential similar to that of both NDPK-D mutants.

We then studied cellular aggregation by disrupting cell-cell contacts with the protease dispase (Figs. 7B, C and 8C, D). Consistent with a highly invasive phenotype, control MDA-MB-231 clones as well as BD and KD clones rendered a low number of aggregates with a diameter over 200 μm^2^ (Fig. 7B, C). In contrast, clones overexpressing WT NDPK-D presented enhanced cell-cell adhesion properties with over 100 aggregates that largely exceeded 200 μm^2^, with sizes even beyond 1000 μm^2^, and a mean area of 600 μm^2^ in both clones (Fig. 7B, C). Conversely, siRNA-mediated knockdown of NDPK-D in ZR75-1 cells resulted in decreased adhesive capabilities compared to controls expressing NDPK-D (Fig. 8C, D). Aggregates of cells transfected with the scramble siRNA presented average areas of 600 μm^2^, whereas those of NDPK-D-depleted cells had only few aggregates over 200 μm^2^ (Fig. 8C, D).

Finally, we tested the effect of NDPK-D depletion on mitochondrial function and oxidative stress. Mitochondrial membrane potential, ΔΨ_m_, was reduced about 20% in NDPK-D-depleted ZR75-1 cells when compared to the control cells expressing NDPK-D (Additional file 18: Fig. S12A**)**. Since mitochondrial dysfunction can result in oxidative stress, we used the mitochondrial superoxide fluorogenic indicator MitoSOX™ Red to selectively detect superoxide species in live cells. MitoSOX™ Red is oxidized by superoxide, resulting in red fluorescence. The ZR75-1 cells depleted of NDPK-D presented a small but significant increase in mitochondrial ROS when compared to control cells expressing NDPK-D (Additional file 18: Fig. S12B), potentially as a result of impaired mitochondrial function.

Taken together, results obtained with breast cancer MDA-MB-231 and ZR75-1 cells are consistent with our data on cervical cancer HeLa cells, namely showing increased cell motility, reduced cell-cell adhesion, and mitochondrial dysfunction with NDPK-D downregulation or loss-of-function mutations. This strongly supports our conclusion that NDPK-D expression is negatively associated with breast cancer progression and invasion.

### Overexpression of NDPK-D reduces in vivo metastasis dissemination

**Fig. 9:**
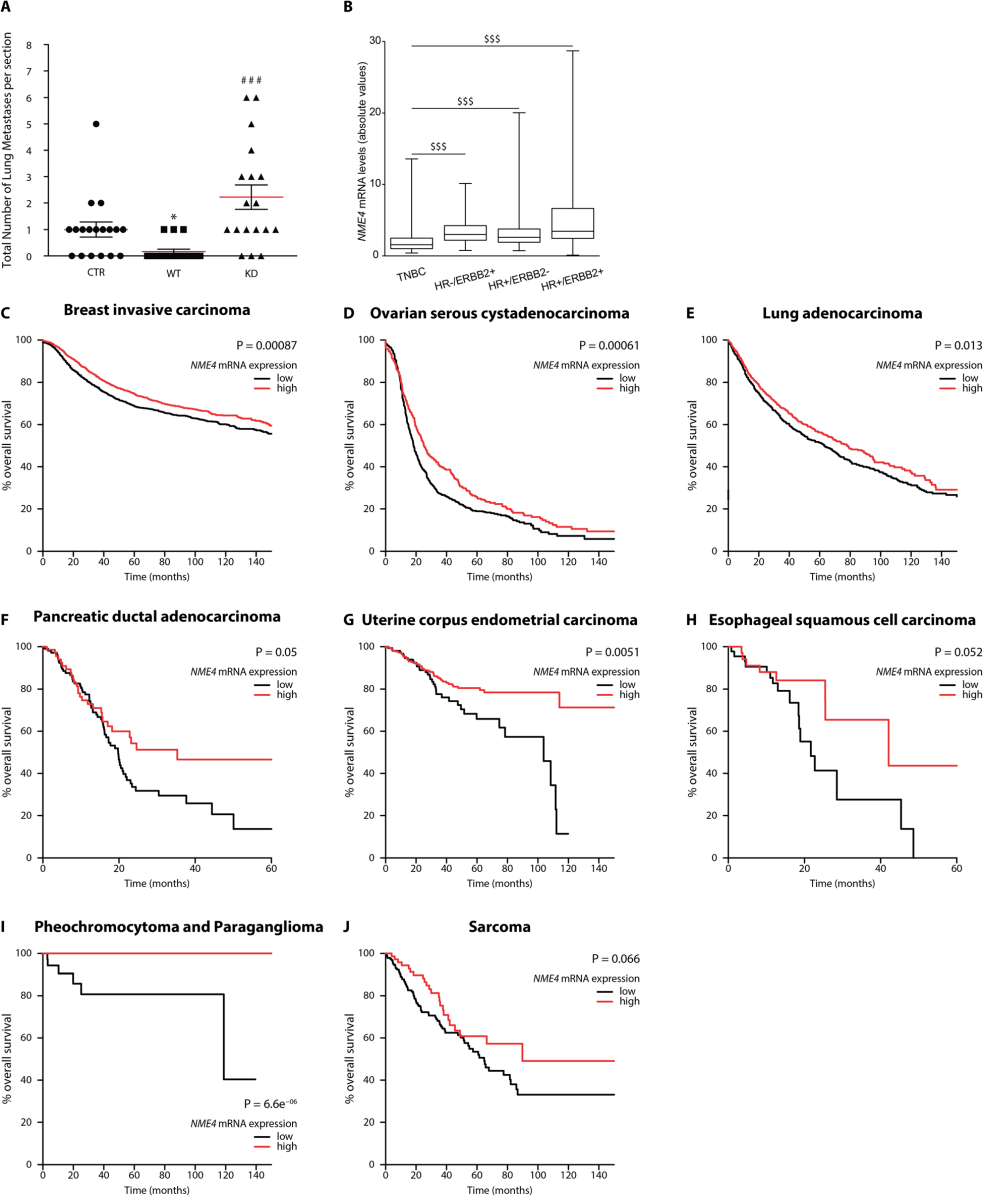
NME4-related metastasis-suppression and prognosis in human cancer. **A** Experimental metastasis assay, where different HeLa clones, empty vector (CTR1, CTR2), wild-type (WT1, WT2), and the kinase-dead mutant (KD1, KD2), were injected in the tail vein of nude mice. After 13 weeks, mice were sacrificed, lungs removed, and the number of lung metastases counted. Total number of lung metastases per section is given after pooling both clones of the same condition (CTR, WT, KD). Eighteen mice of each condition CTR, WT, KD were analyzed. *p<0.05 relative to control/empty vector (CTR); ^###^*p*<0.001 relative to wild-type (WT). **B** mRNA levels of *NME4* in four different human breast tumor subtypes, TNBC (triple-negative breast cancer), HR^-^/ERBB2^+^ (hormone receptors negative, HER2 positive), HR^+^/ERBB2^-^ (hormone receptors positive, HER2 negative), and HR^+^/ERBB2^+^ (hormone receptors positive, HER2 positive) in a cohort of 526 human breast tumor clinical specimens. ^$$$^*p*<0.001. **C**–**J** Kaplan-Meier analysis of overall survival according to *NME4* mRNA expression in KM plotter database of invasive breast carcinoma (**C**), ovarian serous cystadenocarcinoma (**D**), lung carcinoma (**E**), pancreatic ductal adenocarcinoma (**F**), uterine corpus endometrial carcinoma (**G**), esophageal squamous cell carcinoma (**H**), pheochromocytoma and paraganglioma (**I**), and sarcoma (**J**)

As NDPK-D loss-of-function increases the invasive potential of HeLa cells in vitro, we used nude mice to test the role of NDPK-D for metastasis formation in vivo. We injected HeLa cells expressing the different NDPK-D species, empty vector (CTR1, CTR2), wild-type (WT1, WT2), and the kinase-dead mutant (KD1, KD2) via the tail vein of nude mice. After 13 weeks, we found that overexpression of the wild-type NDPK-D reduced pulmonary colonization as compared to empty vector expression condition (Fig. 9A and Additional file 19: Fig. S13). By contrast, overexpression of the KD NDPK-D mutant significantly augmented the number of lung metastases as compared to the wild-type NDPK-D overexpression. These data identify *NME4* as a new metastasis suppressor gene.

### NME4 expression is negatively associated with EMT and tumor invasion markers and is associated with beneficial clinical outcome in human cancer

Based on our novel findings on anti-invasive and anti-metastatic functions of NDPK-D, we predicted that its expression might be down-regulated in human aggressive tumors showing EMT and invasion in comparison to tumors with a good prognosis. We first determined mRNA levels of *NME4* (encoding NDPK-D), *CDH1*, and *KRT18* (encoding the two well-known epithelial markers E-cadherin and cytokeratin 18, respectively), in an important cohort of 526 human breast tumors from patients with well-documented follow-up by using RT-qPCR (Additional file 20: Table S3). Consistently, we found a strong positive association of *NME4* with *CDH1* and *KRT18* (Additional file 21: Fig. S14AB). Strikingly, *NME4* mRNA levels in this cohort were the lowest in the most aggressive human breast tumors with worst prognosis, the so-called triple-negative breast tumors (Fig. 9B). Similar associations were observed with another metastasis suppressor in this cohort: *NME1* (Additional file 21: Fig. S14C-E).

To confirm these findings, we interrogated different transcriptomic databases of human breast tumors. The METABRIC database (1904 human breast tumors) consistently revealed a positive association of *NME4* with epithelial markers *CDH1* and *KRT18* (Additional file 22: Fig. S15A) and a negative association with mesenchymal markers *CDH2* and *VIM* (Additional file 22: Fig. S15B) as well as many EMT drivers like *ZEB1*, *ZEB2*, *SNAI2*, *TWIST1*, and *TWIST2* (Additional file 22: Fig. S15C-E). The *NME4* mRNA levels also negatively associated with the overall EMT score (Additional file 22: Fig. S15FG). The TCGA database (1084 human breast invasive carcinoma samples) revealed similar associations (Additional file 23: Table S4 and additional file 24: Fig. S16), in particular strong positive association of *NME4* with epithelial markers (*KRT18*, the CK8 gene *KRT8*, the plakoglobin gene *JUP*, the ZO-3 gene *TJP3*, and the claudin 3 gene *CLDN3*) and negative association of *NME4* with mesenchymal markers (*VIM*, *CDH2*) and EMT drivers (*SNAI1*, *SNAI2*, *ZEB1*, *ZEB2*). We further observed an overall negative association between *NME4* and tumor invasion markers like genes encoding matrix-degrading proteases (*MMP7*, *ADAM17*) and actin cytoskeleton remodelers (*ROCK1*, *ROCK2*, *LIMK2*, *CFL2*, *MYO5A*). Similar associations were found for other claudins and MMPs, including MMP14 (MT1-MMP) which is essential for matrix degradation during tumor invasion. We further interrogated the TCGA database on human cervical squamous cell carcinoma. This revealed similar associations, in particular positive association with epithelial markers (*KRT18*, *KRT8*, *CLDN3*) and negative association with EMT drivers (*SNAI2* and *ZEB2*). We also observed an overall negative association between *NME4* and tumor invasion markers in this tumor type, in particular between *NME4* and *MMP14*, the key player of invasion, and between *NME4* and markers of invadopodia, the plasma membrane protrusions responsible of matrix degradation and enriched in MMP14 (Additional file 25: Table S5). These data identify low *NME4* expression as associated with EMT and tumor invasion features as a generic trait in human clinical tumor samples.

Finally, we assessed the prognostic value of *NME4* expression in several human tumor cohorts by interrogation of the publicly available human cancer KM Plotter database that contains gene expression data and overall survival information stratifying patient samples into groups of low and high expression. The outcome was then compared between low and high *NME4* expression groups in eight different tumor types. In six carcinoma types (breast, ovarian, lung, pancreatic, uterine, and esophageal carcinoma), low expression of *NME4* was associated with a poor prognosis (Fig. 9C–H); this is also the case for tumors other than carcinomas such as pheochromocytoma, paraganglioma, and sarcoma (Fig. 9IJ). Taken together, these data show that positive association of *NME4* expression with beneficial clinical outcome is a generic trait in cancer.

## Discussion

Cancer cell migration and metastasis belong to the hallmarks of cancer, which severely limit therapeutic options and thus patient survival. Over the past 25 years, more than twenty metastasis-suppressor genes have been described that specifically inhibit metastasis formation without necessarily affecting primary tumor growth [3]. In the present study, we identify the mitochondrial NDPK-D (NME4, NM23-H4) as a novel metastasis suppressor. NDPK-D mutations invalidating either the catalytic NDP kinase activity of the enzyme (KD) or its ability to bind cardiolipin (CL) in the mitochondrial inner membrane (BD) in HeLa and MDA-MB-231 cells, both induced a similar, strong metastatic program. This was apparent by pronounced cellular scattering, loss of intercellular adhesion, increased 2D and 3D cell migration, increased 3D invasion through stromal type I fibrillar collagen, and activation of the small GTPase Rac1 positively regulating cell migration and invasion. Overexpressing WT NDPK-D had an opposite, anti-metastatic effect as compared to controls. Conversely, depletion of NDPK-D in ZR75-1 cells resulted in an increase of migration and a reduction of cell-cell adhesion. A major argument demonstrating the anti-metastatic activity of NDPK-D is the significant reduction of metastasis formation in nude mice in vivo through expression of WT NDPK-D in comparison to controls expressing a low level of NDPK-D, and even more so in comparison to expression of NDPK-D kinase dead mutant. These effects were specific to altered function of mitochondrial NDPK-D and not due to modified expression of cytosolic NDPK-A or -B.

In human tumors, we found a negative correlation between *NME4* expression and metastatic activity or disease outcome. Different cohorts of breast cancer revealed that expression of *NME4* is negatively associated with mesenchymal, EMT and tumor invasion markers, but positively associated with epithelial markers. Examination of a cervical cancer cohort revealed similar associations. Importantly, *NME4* mRNA levels were the lowest in the most aggressive human breast tumors. In breast tumors and several other tumor types, low *NME4* expression was associated with a shorter overall survival, i.e. poor prognosis. Taken together, these data establish NME4 as a suitable prognosis factor.

To date, only few studies addressed *NME4* expression in human cancers as compared to the non-tumoral tissue [34]. Most of these studies show overexpression of *NME4* mRNA in several types of tumors as compared to uninvolved tissue [35]. This is also the case for non-small cell lung cancer, where *NME4* silencing was shown to inhibit proliferation [36]. In oral cancer, *NME4* expression is inhibited by the microRNA miR-196, whose expression is strongly increased in cancer tissue and correlates with lymph node metastasis [37]. Functionally, this onco-miR promoted cell migration, invasion, and lymph node metastasis without affecting cell growth. Taken together, our data and those of available literature indicate that *NME4* expression would increase during formation of the primary tumor and then would decrease when the tumor becomes metastatic. Such biphasic expression has also been reported for other metastasis suppressor genes like *NME1* [38, 39]. It is consistent with *NME4* mRNA levels in human breast tumor cell lines, which are high in hormone receptor-positive cell lines (favorable prognosis), but low in triple-negative cell lines (poor prognosis).

We hypothesized that downstream effectors of NDPK-D function as a barrier against EMT, i.e. against the transition from in situ to invasive carcinoma. Indeed, the morphotypic switch occurring in HeLa cells when expressing mutant as compared to WT NDPK-D and controls was accompanied by profound changes in the cellular proteome, involving more than 150 proteins. These changes were remarkably similar for both NDPK-D mutant cells, often more pronounced for the kinase dead KD, while changes in WT NDPK-D cells relative to controls mostly occurred in the opposite sense, consistent with the cellular phenotype. Expressing NDPK-D mutants altered expression of many metastasis-related proteins, in accordance with a pro-metastatic reprogramming. This includes up-regulation of two proteins closely linked to metastasis, actin-bundling fascin (FSCN1) [40] and S100 protein family member S100A4 (S10A4) [41, 42], further tubulin beta-2A (TBB2A), involved in cancer progression together with other tubulin isotypes [43], and finally γ-synuclein (SYUG), a protein with unknown function but predicting bad prognosis in various cancers [44] and promoting invasion and metastasis in in vitro assays as well as in animal models [45]. FSCN1 upregulation was reported in multiple studies for more aggressive and metastatic epithelial cancers and as a significant, independent prognostic indicator of poor outcome [40]. It is believed to facilitate metastasis by promoting the formation of invasive membrane protrusions called invadopodia and filopodia [46]. S10A4 upregulation was also observed in multiple cancers, while forced down-regulation suppressed the metastatic potential of tumor cells in animal models of lung carcinoma and osteosarcoma [42]. Compelling evidence shows that S10A4 is directly involved in the formation of metastases without affecting the initiation and growth of the primary tumor, hence appearing as a pro-metastatic protein, unlike NDPK-A/NME1 and NDPK-D/NME4 that are metastasis suppressors. Deregulated expression of S100 proteins, mostly up-regulation, occurs in most cancers [41], and we observed that here also for S10A6, S10A8, S10AD, and S10AG. The most highly overexpressed of all proteins, interferon-stimulated gene 15 (ISG15), was more recently also associated with tumor progression. This ubiquitin-like protein conjugates cellular substrates to form ISGylated proteins and can trigger tumorigenesis and metastasis in hepatocarcinoma [47] and breast cancer cell lines [20]. Most changes in protein expression seem to occur at the transcriptional level.

Our data clearly describe the cellular reprograming in both NDPK-D loss-of-function mutants that leads to a metastasis program. However, how this reprogramming is initiated, and why it is highly similar for the two NDPK-D mutations that affect different and independent protein functions? We have found very similar effects of the two mutants already at the mitochondrial level for a large majority of analyzed parameters: fragmentation of the mitochondrial network, loss of mitochondrial mass, downregulated mitochondrial proteins, reduced cellular respiration, increased aerobic glycolysis, altered Krebs cycle activity, reduced sensitivity of mitochondrial permeability transition for inhibition, oxidative damage, and mild energy stress linked to activation of AMPK signaling together with increased expression of mitochondrial umtCK and AK2. Out of these changes, only fragmentation of the mitochondrial network can be directly linked to NDPK-D dysfunction and may thus constitute the primary event. As we found earlier, NDPK-D forms a complex with the pro-fusion motor protein OPA1 at the inner mitochondrial membrane, mediated by CL-binding of both partners [9, 10, 12]. In this complex, NDPK-D fuels the OPA1 GTPase with the necessary GTP for its inner membrane fusion and remodeling activities [11]. Silencing of either NDPK-D or OPA1 leads to a similar, fragmented mitochondrial network by disturbing the fission/fusion equilibrium. Importantly, the GTP fueling by NDPK-D is not only abrogated by the KD mutant, but also reduced by the BD mutant, since in this case, complex formation with OPA1 is inhibited [10–12]. Both mutants would thus reduce the channeling of GTP between NDPK-D and OPA1 [48], since catalytic activity is either deleted or improperly located due to the altered CL-binding. As a consequence, both mutations would thus reduce mitochondrial fusion and trigger fragmentation. As seen in HeLa cells, this seems to be a dominant negative effect, since the low endogenous NDPK-D levels which do maintain a control phenotype close to WT overexpressors do not so in case of mutant overexpression. There also seems to be a dose effect, since HeLa control clones expressing low but detectable levels of NDPK-D behave similar, but not identical to WT-overexpressing clones, while MDA-MB-231 control clones with undetectable NDPK-D levels are clearly different to WT overexpressors and behave similar to the loss-of-function BD and KD mutants. Important for our study, also overexpression of OPA1 was shown to reduce cell migration and invasion in multiple cancer types and even tumor progression in vivo [49]. Mechanistically, mitochondrial fragmentation is known to facilitate the trafficking of mitochondria to the leading edge of the migrating and invasive cancer cell, where they fuel membrane dynamics and cell movements [49–53]. However, OPA1 mutations, responsible for optic atrophy and neurological disorders, seem not to be associated with cancer.

Most of the other mitochondrial phenotypes that we observed could be a direct consequence of mitochondrial fragmentation. It is well known that fragmentation, i.e. the presence of smaller mitochondria, facilitates elimination of mitochondria by mitophagy [54, 55]. Reduced mitochondrial mass then explains the metabolic shift consisting in a decrease in cellular respiration and a compensatory increase in glycolytic activity. There may be also additional effects on respiratory complex I as evidenced by altered subunit expression, rotenone inhibition of mtPTP, and an increase in cellular ROS generation leading to oxidative damage. However, this issue requires further analysis before definite conclusions can be made. Mitochondrial fragmentation and elimination would further induce a mild energy stress as revealed by activated AMPK signaling and upregulation of mitochondrial kinases (umtCK, AK2) that handle high-energy phosphates and localize to the intermembrane space like NDPK-D. Further metabolic reprogramming seems to occur in the Krebs cycle. Activity of CS, the enzyme catalyzing the first committed step at the cycle’s entry point, and abundance of isocitrate dehydrogenase (IDH3A) increase with WT NDPK-D expression, but decrease with NDPK-D mutant expression as compared to controls. Indeed, NDPK-D loss-of-function may directly interfere with the Krebs cycle due to its matrix-localized portion [9]. Here, it can functionally interact with succinyl coenzyme A synthetase (succinylthiokinase) to convert the generated GTP into ATP [56, 57].

How mitochondrial dysfunction then leads to metastatic reprogramming? In fact, changes in mitochondrial structure and function are increasingly recognized as important determinants not only for cancer but also for the metastatic process [58, 59]. In particular fragmentation of the mitochondrial network facilitates invasion and migration of cancer cells, while a fused mitochondrial network is rather inhibitory [55]. Generally, metastatic cancer cells have lower levels of another pro-fusion protein, MFN, and higher expression of pro-fission DRP1 [50, 60–62]. Experimentally, stimulating DRP1 [51] or silencing MFN [50] increases metastatic potential, while silencing or pharmacologically inhibiting DRP1 or overexpressing MFN reduces cell migration and metastasis formation [50, 60, 63, 64]. Also, EGF-induced mitochondrial localization of EGFR favors mitochondrial fission and thus increases cell motility and metastasis [65], consistent with increased EGF signaling in both mutant NDPK-D clones as compared to WT NDPK-D cells.

Mitochondrial fragmentation and dysfunction would then trigger further potential retrograde signals. For example, AMPK signaling has multi-faceted aspects in cancer, but most recent studies point to roles of activated AMPK in promoting EMT and metastasis [66, 67]. Further, increased ROS generation in NDPK-D mutant cells could mediate pro-metastatic gene expression and metastatic phenotypes as shown in different models of mitochondrial impairment like mtDNA mutations [68], inhibition of autophagy [69], or altered mitochondrial calcium homeostasis [70]. Finally, metabolic reprogramming can favor EMT and generate pro-metastatic conditions. Increased expression of umtCK and AK2 in loss-of-function clones could be more than a compensatory adaptation to energy stress [71]. In particular, umtCK was associated with bad prognosis and metastasis [30, 72, 73]. Also reduced CS activity may favor metastasis, since its knockdown can induce an EMT phenotype and enhance metastasis in cervical carcinoma cells, although the contrary can occur in other cancers [74, 75]. Thus, reduced function of mitochondrial NDPK-D would act via different, complex pathways to promote metastasis, as typical for metastasis suppressors in general, but mechanistically different to what is known about metastasis suppression by the cytosolic isoforms NDPK-A and -B [3].

## Conclusions

Collectively, our data reveal a prominent role of altered NDPK-D in crucial features of cancer metastasis such as loss of intercellular adhesion, migration, invasion, and EMT. *In fine*, they suggest a communication between mitochondria, cytosol, and nuclear genes for a pro-metastatic reprogramming of cellular protein expression as the driving force towards the observed morphotypic switch. Definitely, our in vitro, in vivo, and clinical findings show for the first time that yet another member of the NME/NDPK family, *NME4,* is a new metastasis suppressor gene, and the first one localized in mitochondria, and that *NME4* has the potential of being a strong prognostic biomarker. Future studies will have to dissect the underlying basic mechanisms in more details. In perspective, targeting dysregulated mitochondrial fission/fusion dynamics may provide a novel strategy for inhibiting cancer metastasis.

## Methods

### Materials

T-Rex^TM^ HeLa cells and the pcDNA4/TO vector were obtained from Invitrogen (ThermoFischer Scientific, Waltham, MA, USA). MDA-MB-231 cells were a kind gift of Dr Patricia Steeg (NIH, Bethesda, MD, USA). Constructs to express the NDPK-D WT or NDPK-D mutated at His151 in the catalytic site (KD) or at Arg90 at the CL binding site (BD) were obtained as described [9]. Recombinant expression and purification of NDPK-D, as well as generation of anti-human NDPK-D polyclonal antibodies in rabbits are described elsewhere [8, 9]. Specific primary antibodies against NDPK-A and B were obtained and used as described in Boissan et al. [39]. Mouse monoclonal antibodies anti-Mn-superoxide dismutase (SOD), anti-S100A4, anti-Fascin, anti-alpha-tubulin, and anti-tubulin beta II were from Bender Medsystems GmbH (Vienna, Austria), Abnova (Taipei, Taiwan), Agilent (Santa Clara, CA, USA), Sigma-Aldrich (St-Louis, MO, USA) and Abcam (Cambridge, MA, USA), respectively. Rabbit monoclonal anti-gamma synuclein was obtained from Abcam. Polyclonal goat anti-ISG15, mouse monoclonal anti-phospho-Thr^202^Tyr^204^ ERK1/2, and rabbit polyclonal anti-ERK1/2 and cyclin A were from Santa Cruz Biotechnology Inc. (Santa Cruz, CA, USA). Rabbit monoclonal anti-phospho-Tyr^1068^ EGFR, rabbit monoclonal anti-EGFR, rabbit polyclonal anti-phospho-Ser^473^ AKT, rabbit polyclonal anti-AKT, rabbit polyclonal anti-phospho-Ser^9^ GSK3β, mouse monoclonal anti-GSK3β, rabbit polyclonal anti-phospho-^Ser199/204^ PAK1, anti-PAK1, anti-AMPKα, anti-phospho-^Thr172^ AMPKα, anti-ACC, and anti-phospho-^Ser79^ ACC were from Cell Signaling Technology Inc. (Beverly, MA, USA). Mouse monoclonal anti-RAC1 was from BD Biosciences (San Jose, CA, USA). Mouse monoclonal anti-Cyclin B1 and PCNA were from Neomarkers (Fremont, CA, USA) and DakoCytomation (Glostrup, Denmark), respectively. Rabbit polyclonal anti-AK2 was from Abgent (San Diego, CA, USA) and anti-umtCK was described earlier [76].

Pharmacological inhibitors of PI3K (GSK2126458) and Src (MA475271) were obtained from GlaxoSmithKline (GSK, Brentford, UK) and AstraZeneca (AZ, London, UK), respectively. Pharmacological inhibitors of p38 (SB203580), JNK (SP600125), and EGFR (lapatinib) were purchased from Selleckchem (Houston, TX, USA). Human EGF was purchased from PeproTech (Rocky Hill, NJ, USA).

### Cell culture and preparation of cellular and mitochondrial extracts

T-Rex^TM^ HeLa cells were stably transfected with the vector pcDNA™4/TO without insert (control) or with an insert coding for the NDPK-D WT or the NDPK-D mutants (KD and BD) as described [9]. HeLa clones, grown in MEM medium as described [9], overexpress comparable levels of NDPK-D proteins already without specific induction. Maximal expression levels can be achieved by incubation with 1 μg/ml tetracycline for 24 h. For cell extract preparation, cells were grown in 3.5 cm diameter Petri dishes or in 6 well plates, were rinsed twice with ice-cold PBS and lysed in 50 μl RIPA/well containing anti-proteases (Calbiochem, cocktail set III or Complete®, Sigma-Aldrich), anti-phosphatases (Sigma-Aldrich, cocktail n°2) and 1 mM EDTA. The lysate was either used immediately or frozen in liquid nitrogen and stored at -20 °C until use. For citrate synthase activity measurements, the fresh lysate was sonicated for additional 5 s at 50% power and centrifuged at 10,000×*g* for 20 min at 4 °C and the supernatant kept. Crude HeLa mitochondria were isolated by differential centrifugation according to Eskes et al. [77]. The protein concentration was determined by a BCA protein assay (Pierce), using bovine serum albumin (BSA) as standard.

MDA-MB-231, ZR75-1, and HBL100 cell lines were cultured in DMEM containing 10% fetal bovine serum (FBS). BT-474, BT-549, HCC-1428, MDA-MB-468 cells were grown in RPMI-1640 medium containing 10% FBS and 100 U/mL penicillin and 100 μg/mL streptomycin (P/S). HCC-1143, HCC-1187, HCC-1599, HCC-1500, and HCC-1937 cells were grown in RPMI-1640 medium containing 10% FBS, P/S, 1.5 g/L sodium bicarbonate, 10 mM Hepes and 1 mM sodium pyruvate. T47D cells were grown in RPMI-1640 medium containing 10% FBS, P/S and 0.2 U/mL bovine insulin. BT-483 cells were grown in RPMI-1640 medium containing 20% FBS, P/S and 0.01 mg/mL bovine insulin. MCF-10A, MCF-10-2A, and 184B5 cells were grown in DMEM-F12 containing 5% horse serum, 20 ng/mL EGF, 100 ng/mL cholera toxin, 0.01 mg/mL insulin and 500 ng/mL hydrocortisone. MCF-12A cells were grown in DMEM-F12 containing 5% horse serum, 20 ng/mL EGF, 100 ng/mL cholera toxin, 0.01 mg/mL insulin, 500 ng/mL hydrocortisone, 1.2 g/L sodium bicarbonate, 0.5 mM sodium pyruvate and 15 mM Hepes. HMEC and hTERT-HME1 cells were grown in Mammary Epithelial Cell Growth Medium BulletKit (Lonza, Basel, Switzerland). Hs578T and MDA-MB-361 cells were grown in DMEM containing with 10% FBS and P/S. BT-20 and MCF-7 cells were grown in MEM containing 10% FBS, 1.5 g/L sodium bicarbonate, 0.1 mM non-essential amino acids and 1 mM sodium pyruvate. MDA-MB-157 and MDA-MB-453 cells were grown in Leibovitz’s L-15 medium containing 10% FBS, P/S and 10 mM Hepes. MDA-MB-415 cells were grown in Leibovitz's L-15 medium containing 15% FBS, P/S, 10 mM Hepes, 0.01 mg/mL insulin and 0.01 mg/mL glutathione. CAMA1 cells were grown in Eagle’s MEM containing 10% FBS and P/S. MDA-MB-435S cells were grown in Leibovitz’s L-15 medium containing 10% FBS, P/S, and 0.01 mg/mL insulin. All cell lines were maintained at 37 °C in a humidified atmosphere with 5% CO_2_.

### Immunoblot analysis

Proteins from cell extracts were electrophoretically separated on 10% or 12.5% SDS polyacrylamide gels and transferred onto Immobilon P membranes (0.1 μm, Merck Millipore, Burlington, MA, USA) for 2 h at 22 V in 10 mM CAPS buffer, pH 11, 10% methanol for NDPK-D, as described in [9], or onto nitrocellulose membranes for 90 min at 50 V in 0.025 M Tris-base, 0.192 M glycine, 20% methanol, and 0.02% SDS for the other proteins. The polyclonal anti-NDPK-D was diluted 1/7500, the anti-α tubulin (loading control) 1/5000, and the other primary antibodies 1/500. Blots were revealed with appropriate peroxidase-coupled secondary antibodies and ECL Plus substrate (GE Healthcare, Chicago, IL, USA). Images of the full immunoblots can be found in Additional file 26.

### Cell dispersion, aggregation, invasion, and migration assays

Cellular spatial distribution was characterized and quantified using algorithmic programs of cellular sociology based on the use of three previously described geometrical models, namely Voronoï's partition, Delaunay’ graph, and minimum spanning tree (MST) as described [16, 78]. The aggregation assay was performed as reported [17] by seeding cells on top of a gelified agar medium. Aggregate formation was scored under an inverted microscope at × 10 magnification after 24 h incubation at 37 °C. Native type I collagen invasion assays were performed as described earlier [78–80]. Two- and three-dimensional migration assays are described in [16, 78] and [81], respectively.

### Cell dispase assay

Confluent monolayers of cells were washed with ice-cold PBS and separated from the plates by incubation with PBS free of Ca^2+^ and 0.6 U/mL of dispase I (MP Biomedicals, Irvine, CA, USA) for 35 min at 37 °C. The dispase solution was removed by centrifuging the cells for 2 min at 400×*g* and replaced by 200 μL of PBS. The cells were mechanically separated by pipetting up and down five times with a 200-μL pipette. The aggregates were observed by light microscopy using the 10X objective (Echo Rebel Microscope, San Diego, CA, USA). The size of the aggregates was measured using the Fiji software (aggregates < 200 μm^2^ were excluded from the quantification).

### Wound healing assay

Cells were grown until confluence on 24 well plates in DMEM supplemented with 10% FBS and antibiotics. Cells were starved for 24 h in DMEM without FBS and treated for 2 h with Cytosine β-D-Arabinofuranoside (AraC) to inhibit cell proliferation during the experiment. After starvation, cells were scratch-wounded using a sterile 200 μL pipette tip and suspended cells were removed by washing with PBS twice. The progress of cell migration into the wound was monitored every 24 h until wound closure using the 10X objective of an Echo Rebel Microscope (San Diego, CA, USA). The bottom of the plate was marked for reference, and the same field of the monolayers were photographed immediately after performing the wound (time = 0 h) and at different time points after performing the scratch.

### Matrix metalloprotease activity by gelatin zymography

Culture media were collected and concentrated using 10 KDa cut-off ultra-centrifugal filter units (Amicon, Merck-Millipore, Burlington, MA, USA). Protein concentration was determined by the Bradford method, and 200 μg of concentrated supernatant proteins were assayed for proteolytic activity on gelatin-substrate gels. Briefly, samples were mixed with non-reducing loading buffer containing 2.5% SDS, 1% sucrose, and separated in 8% acrylamide gels co-polymerized with 1 mg/mL gelatin. Electrophoresis was conducted at 80 V for 2.5 h, then the gels were rinsed twice in 2.5% Triton X-100, and then incubated in 50 mM Tris-HCl pH 7.4 and 5 mM CaCl_2_ assay buffer at 37 °C for 24 h. Gels were fixed and stained with 0.25% Coomassie Brilliant Blue G-250 in 10% acetic acid and 30% methanol. Proteolytic activity was detected as clear bands against the background stain of undigested substrate in the gel. Quantification was performed using ImageJ2 software (NIH, Bethesda, MD, USA).

### Enzyme activities

NDPK activity in mitochondrial extracts (1-10 μg protein/assay) was measured spectrophotometrically by a coupled pyruvate kinase-lactate dehydrogenase assay using 0.2 mM ATP and 0.2 mM TDP as substrates and adding 100 μM Ap5A to inhibit endogenous adenylate kinase, as described previously [8, 9]. As an estimate of mitochondrial mass, citrate synthase (CS) activity was measured in cell lysates in the presence of 150 mM Tris pH 8, 150 μM 5,5′-dithiobis-(2-nitrobenzoic acid) (DTNB), 300 μM acetyl-coenzyme A and 500 μM oxaloacetate. Reduction of DTNB by CS at 37 °C was followed spectrophotometrically at 412 nm and CS activity calculated in nkat/mg of total protein.

### Rac1 pulldown assay

Cells were seeded overnight and starved in low serum for 24 h then washed twice with ice cold PBS supplemented with MgCl2 (25 mM) and lysed in lysis buffer (50 mM Tris-HCl pH 7.5, 1% Triton X-100, 25 mM MgCl2, 500 mM NaCl, 2 mM sodium pyrophosphate, 1 mM NaVO4, 2 mM phenylmethylsulphonyl fluoride (PMSF), 10 μg/mL aprotinin, 10 μg/mL leupeptin). Lysates were clarified by centrifugation, and equal amounts of protein lysates were incubated with 20 μL of purified glutathione S-transferase (GST)-CRIB (Cdc42/Rac interactive binding motif) immobilized on glutathione-Sepharose beads (GE Healthcare, Chicago, IL, USA) for 1 h rocking at 4 °C. The beads were washed 3 times in lysis buffer and boiled for 10 min in SDS sample buffer (62.5 mM Tris-HCl pH 6.8, 10% glycerol, 0.002% bromophenol blue, 2% SDS, and 5% β-mercaptoethanol). The samples were analyzed for Rac1 pull-down by Western blot using an anti-Rac1 antibody.

### Mitochondrial network analysis

For cellular staining of mitochondria, cells cultivated on microscope glass slides were fixed in 3.7% paraformaldehyde, permeabilized in PBS containing 0.5% Triton, blocked in PBS containing 3% BSA, and incubated with anti-mouse MnSOD primary antibody (dilution 1:150) and with donkey anti-mouse IgG secondary antibody, Alexa Fluor 488-conjugate (dilution 1:150). Slides were examined with a Leica HC microscope. For mitochondrial staining with a mitochondrion-selective dye and NDPK-D immunodetection, cells were incubated with 50 nM MitoTrackerTM Red CMXRos (ThermoFisher Scientific, Waltham, MA, USA) for 30 min at 37 °C before fixation, and then treated as described above before incubation with anti-NDPK-D affinity-purified antibody and followed by the Alexa-Fluor 488-conjugated secondary anti-rabbit antibodies. Before examination, slides were mounted with Fluoromount-G (Electron Microscopy Sciences, Hatfield, PA, USA).

Fixed cells immunostained for mitochondrial MnSOD were subjected to image analysis with Image J software to extract foreground and segment mitochondria using an adaptive threshold (“top-hat filtering”). In the resulting binary image of the mitochondrial network, regions of interest (ROI) were selected in peripheral regions of cells, where individual mitochondrial elements can be more easily detected as compared to the mitochondrial clusters close to the nucleus. Morphometric analysis of each ROI was done with Volocity v.4.0.1 software (Improvision, France), which yielded values for average length and the elongation factor, calculated as (mean_perimeter^2^)/(4 π mean_area). To visualize the mitochondrial network in living cells, Hela cells grown on Labtek® plates (ThermoFisher Scientific, Waltham, MA, USA) were labeled with 100 nM MitoTracker Green FM (ThermoFisher Scientific, Waltham, MA, USA) for 20 min at 37 °C. Images were acquired with a confocal laser-scanning microscope (Leica TCS SP2 AOBS; 488 nm excitation, 500–550 nm detection), equipped with a perfusion chamber (LaCon), and an incubation system (O_2_–CO_2_–°C, PeCon). Confocal pinhole (set to 1 Airy) and all other parameters were kept constant for all experiments, and images were taken from randomly chosen fields containing 8–12 cells.

### Mitochondrial and glycolytic function

Mitochondrial membrane potential was determined with about 10^6^ HeLa cells per sample, first incubated for 30 min at 37 °C with 50 nM TMRM (tetramethylrhodamine, methyl ester, ThermoFisher Scientific, Waltham, MA, USA), a membrane potential sensitive dye, and 100 nM Mitotracker Green^FM^ (ThermoFisher Scientific, Waltham, MA, USA). Cells were then centrifuged at 700*g* for 4 min at 4 °C, the pellet was resuspended in 1 ml PBS, and TMRM fluorescence gated by Mitotracker signal was analyzed by FACS (BD LSR FORTESSA, Becton Dickinson, Le Pont-de-Claix, France). Cells were then incubated with 1 μl 50 mM CCCP for another 5 min at room temperature to entirely depolarize the mitochondria, and again analyzed by FACS. About 20,000–50,000 events gated on Mitotracker fluorescence were measured, and differences in samples before and after the addition of CCCP were calculated as readout for mitochondrial membrane potential. Laser excitation was 488 nm and 532 nm for Mitotracker Green^FM^ and TMRM, respectively. Fluorescence emission was collected with a 530/30 nm band-pass filter for Mitotracker Green^FM^ and 585/15 nm band-pass filter for TMRM.

Changes in mitochondrial membrane potential produced by NDPK-D knockdown in ZR75-1 cells were determined with the tetramethylrhodamine ethyl ester (TMRE)-Mitochondrial Membrane Potential Assay Kit (Abcam, Cambridge, MA, USA) following the manufacturer’s protocol. Briefly, ZR-75-1 cells were supplemented with 200 nM TMRE and incubated in the dark for 10 min at 37 °C. Then, the cells were trypsinized and washed three times with PBS. Fluorescence intensity of TMRE was measured by Spectrum Cellometer (Nexcelom Biosciences, Lawrence, MA, USA) by setting the filter excitation at 502 nm and emission at 595 nm, as previously reported [82, 83]. Data was analyzed with FCS Express 7 (De Novo Software).

Oxygen consumption in intact HeLa cells was measured in a thermostatically controlled Clark electrode oxygraph at 37 °C (Strathkelvin MS200A system). HeLa cells were detached by trypsin and counted. A cell suspension (100 millions of trypan blue negative cells per ml) was prepared in Roswell Park Memorial Institute (RPMI) medium (ThermoFisher Scientific, Waltham, MA, USA). Five million cells were added in the oxygraph chamber containing RPMI medium to a final volume of 500 μl. Oxygen consumption of cells was measured with succinate as substrate before and after the addition of oligomycin (0.06 μg/ml) and FCCP (0.5 μM), and results expressed as nmol O_2_ per minute per mg of cellular protein.

Calcium retention capacity in digitonin permeabilized cells was determined in trypsin-detached HeLa cells (2 million trypan blue negative cells), permeabilized immediately before use by incubation under stirring for 2 min at 30 °C in 250 mM sucrose, 10 mM Tris-MOPS, 1 mM Pi-Tris (pH 7.4) supplemented with 100 μg/ml digitonin. Initially, 0.25 μM Calcium Green-5 N (ThermoFisher Scientific, Waltham, MA, USA) was added, followed by 5 mM succinate, to a final volume of 1 ml. The calcium retention capacity was measured by sequential addition of 12,5 μM Ca^2+^ pulses until permeability transition occurs [28]. Extramitochondrial Ca^2+^ was measured fluorometrically at 30 °C using a PTI Quantamaster C61 spectrofluorimeter (excitation: 506 nm; emission: 530 nm) [29]. Results are expressed as nmol Ca^2+^ per 2 millions of trypan blue negative cells.

Extracellular acidification rate (ECAR) to estimate glycolytic activity was determined by an Agilent Seahorse XF flux analyzer according to manufacturer’s instructions.

### ROS and oxidative stress

ROS production was detected using the dye *CM*-*H2DCFDA.* Cells were incubated with CM-H2DCFDA (9 μΜ) in DMEM without FBS. Quantification was performed with a plate fluorescence reader (Spectrafluor Plus, Tecan-France, Trappes, France) at 520 nm [84]. Further markers of oxidative stress were analyzed as described in [85], including protein oxidation by thiols groups (SH) [86] and Ferric Reducing Ability of Plasma (FRAP) by ferric reduction [87]. The lipid hydroperoxides were determined using a lipid hydroperoxide assay kit (Cayman Chemical Co., Ann Arbor, MI, USA) according to the manufacturer’s instructions.

To quantify changes in oxidative stress produced in the mitochondria, cells were incubated with 5 μM MitoSOX™ reagent dissolved in DMSO, following the manufacturer’s instructions. After 10 min incubation at 37 °C in the dark, the cells were trypsinized and washed three times with PBS. Fluorescence was recorded using the Spectrum Cellometer (Nexcelom Biosciences, Lawrence, MA, USA) with excitation/emission maxima of 510/580 nm. Data was analyzed with FCS Express 7 (De Novo Software).

### Measurement of intracellular nucleotides by LC-HRMS

Cell extracts were prepared by cell lysis with the cold mixture methanol/water (70/30, v/v) after removing of cell medium and two washes with cold PBS. Extracts were stored at − 80 °C before analysis. Analysis of nucleoside mono-, di- and triphosphates in cell extracts was performed on an Ultimate 3000 liquid chromatography system (ThermoFisher Scientific, Waltham, MA, USA) coupled with a Q-Exactive Plus Orbitrap mass spectrometer (ThermoFisher Scientific, Waltham, MA, USA) using a validated method [88]. Results were expressed as the ratio of triphosphate/diphosphate which corresponds to area of the nucleoside triphosphate peak/area of the nucleoside diphosphate peak and as the ratio of triphosphate/monophosphate which corresponds to area of the nucleoside triphosphate peak/area of the nucleoside monophosphate peak.

### Proliferation assays

Cell proliferation was examined in real-time using the xCELLigence RTCA MP System (Roche Molecular Systems, Pleasanton, CA, USA). HeLa clones were seeded at 5000 cells/well into 96-well plates and proliferation was continuously monitored every hour over a time period between 12 and 36 h. Data analysis was performed using RTCA 1.2 software supplied with the instrument. Levels of proliferation markers, cyclin A, cyclin B1, and PCNA were analyzed by Western blotting of HeLa clone extracts.

### 2D-DIGE proteomic analysis

#### Sample preparation for 2D-electrophoresis

Cells were grown in 6 cm diameter Petri dishes close to confluence (10^6^ cells), washed in cold PBS, harvested with a rubber policeman and pelleted by centrifugation at 800×*g* for 5 min. Pellets, dried by aspiration, were frozen in liquid nitrogen and stored at -80 °C until use. Pellets were lysed and homogenized 20 min on ice in 100 μL of UTCD buffer (8 M urea, 2 M thiourea, 4% CHAPS, and 50 mM dithiothreitol (DTT)). The lysates were centrifuged at 20,000×*g*, at 4 °C for 1 h. The supernatants were collected and proteins were precipitated with a 2-D Clean-Up Kit (GE Healthcare, Chicago, IL, USA) following the manufacturer’s instructions. The pellets were solubilized in 100 μL of UTC buffer (UTCD buffer without DTT) and the protein concentration determined using Quick-Start Bradford Dye Reagent (Bio-Rad, Hercules, CA, USA).

#### Two-dimensional differential in-gel electrophoresis (2D-DIGE)

Three independent samples of two independent clones for each condition (control HeLa-Trex cells transfected with empty vector (CTR1A, B, C; CTR2B, C, D); cells overexpressing the wild-type NDPK-D (WT1A, B, C; WT2A, C, D), the catalytically inactive (KD1A, B, D; KD2A, B, C) and the CL-binding-deficient enzyme (BD1A, B, C; BD2A, B, D) were analyzed by 2D-DIGE. Fifty micrograms of proteins of each sample were labeled with Cy3 or Cy5 CyDye™DIGE Fluor minimal dyes (GE Healthcare, Chicago, IL, USA) following the manufacturer’s instructions. The internal standard (IS) was prepared by mixing equal amounts of each sample and labeled with Cy2. Fifty micrograms of labeled samples (Cy3 or Cy5) and internal standard (Cy2) were mixed in twelve different combinations as follows: WT1A-Cy3/CTR1A-Cy5/IS-Cy2, WT2A-Cy3/CTR2B-Cy5/IS-Cy2, KD2A-Cy3/WT2C-Cy5/IS-Cy2, WT1B-Cy3/KD1A-Cy5/IS-Cy2, BD1A-Cy3/WT2D-Cy5/IS-Cy2, BD2A-Cy3/WT1C-Cy5/IS-Cy2, KD1B-Cy3/CTR1B-Cy5/IS-Cy2, CTR2C-Cy3/KD1D-Cy5/ IS-Cy2, BD1B-Cy3/KD2B-Cy5/IS-Cy2, KD2C-Cy3/BD2B-Cy5/IS-Cy2, CTR1C-Cy3/BD1C-Cy5/IS-Cy2, and CTR2D-Cy3/BD2D-Cy5/IS-Cy2. Each of the twelve mixes (150 μg) was analyzed by 2D-DIGE as previously described with minor modifications [89]. Protein separation was performed by isoelectrofocusing on 18-cm pH 3**–**11NL Immobiline™ Drystrips (IPG strips, GE Healthcare, Chicago, IL, USA) in the first dimension and SDS-PAGE on twelve different 8 to 18% acrylamide gels in the second dimension. Cy2, Cy3, and Cy5 components of each gel were individually imaged as described previously [89].

#### Statistical analysis

Image analysis, relative quantification of spot intensity, statistical evaluation using one-way ANOVA followed by a Tukey’s multiple comparison test and PCA (principal component analysis) were carried out with DeCyder 7.2 software (GE Healthcare, Chicago, IL, USA). Normalization across all gels was performed using the internal standard. A spot was considered as differentially represented between two sample groups if the following conditions were fulfilled: *p* value below 0.05 and protein abundance fold change above + 1.3 or below **−** 1.3.

#### Protein identification by Mass Spectrometry (MS) and database searching

For MS identification of proteins of interest, two distinct semi-preparative 2D-gels were prepared using 400 μg of WT and 400 μg of a mix of BD and KD, respectively, to rehydrate the IPG strips. After electrophoresis, 2D-gels were fixed and stained as described in [90]. Gels were scanned using a Typhoon 9400 Trio Variable Mode Imager (GE Healthcare, Chicago, IL, USA) at 488/520 nm, 100 μm resolution. Spots of interest were excised using the Ettan spot picker (GE Healthcare, Chicago, IL, USA). In-gel digestion was carried out with trypsin, according to a published procedure with minor adjustments [91] and using for all steps a Freedom EVO 100 digester/spotter robot (Tecan, Switzerland). For MS and MS/MS ORBITRAP, analyses were performed using an Ultimate 3000 Rapid Separation Liquid Chromatographic (RSLC) system (Thermo Fisher Scientific, Waltham, MA, USA) online with a hybrid LTQ-Orbitrap-Velos mass spectrometer (ThermoFisher Scientific, Waltham, MA, USA). The Linear Trap Quadrupole Orbitrap mass spectrometer acquired data throughout the elution process and operated in a data-dependent scheme with full MS scans acquired with the Orbitrap, followed by up to 20 LTQ MS/MS CID spectra on the most abundant ions detected in the MS scan. The fragmentation was permitted for precursors with a charge state of 2, 3, 4, and above. For the spectral processing, the software used to generate mgf (Mascot generic format) files was Proteome discoverer v1.4.0.288. The threshold of Signal to Noise for extraction values is 3. Database searches were carried out using Mascot version 2.4 (Matrix Science, London, UK) on “*homo sapiens*” proteins (20,345 sequences) from the SwissProt databank containing 542,503 sequences (192,888,369 residues) (February 2014). The search parameters were as follows: carbamidomethylation as a variable modification for cysteines, and oxidation as a variable modification for methionines. Up to 1 missed tryptic cleavage was tolerated, and mass accuracy tolerance levels of 10 ppm for precursors and 0.45 Da for fragments were used for all tryptic mass searches. Positive identification was based on a Mascot score above the significance level (i.e., 5%).

### RNA interference

Two specific siRNAs targeting *NME1* (Si1 5′-GGCUGUAGGAAAUCUAGUU; Si2 5′-GGAUUCCGCCUUGUUGGUC) or targeting *NME4* (Si1 5′ -AGCACAAGAUUGGACCAAU; Si2 5′ -GCAAGAACCCAAGCCCACA) synthesized by ThermoFisher Scientific (Waltham, MA, USA) were used. The siRNA control sequence was 5′-GGCUGUAGAAGCUAUAGUU. Cells were transfected with control or specific siRNA sequence using the DharmaFECT 4 transfection reagent (Dharmacon, Inc, Lafayette, CO, USA).

### Experimental metastasis assays

All the animal experiments were carried out at NCI (Frederick, MA, USA) under an approved NCI-Animal Use Agreement. HeLa cells stably expressing different constructs (CTR1, CTR2, WT1, WT2, KD1, KD2) were trypsinized, washed, and resuspended in PBS and injected into the lateral tail vein (n=9 for each group) of 6-week-old Balb/c athymic nude female mice (1 × 10^6^ HeLa cells per injection). Thirteen weeks post-injection, at necropsy, the lungs were collected and fixed in Bouins' solution. Lung metastatic lesions were counted using H&E section and reported as a mean for each group.

### RT-qPCR (HeLa cell lines)

Quantitative PCR was performed on HeLa stable cell lines using the mix 2X Roche LightCycler (480 SY Green Master Mix- ref 4 887 352 001- Roche Diagnostics, Mannheim, Germany) on a Light Cycler 96 Real-Time PCR Roche (Roche Diagnostics, Mannheim, Germany). Data from each sample were normalized on the basis of its content in HPRT (hypoxanthine-guanine phosphor-ribosyl transferase) transcripts. The primers used were: *ISG15* (upper primer, 5′-GAGAGGCAGCGAACTCATCT-3′; lower primer, 5′-CTTCAGCTCTGACACCGACA-3′), *S10A4* (upper primer, 5′-GAGCTGCCCAGCTTCTTG-3′; lower primer, 5′-TGCAGGACAGGAAGACACAG-3′), *FSCN1* (upper primer, 5′-GGCAATGGACAGAGGACAGT-3′; lower primer, 5′-AAAATGCCTTGGCTTGAATG-3′), *CDH2* (upper primer, 5′-GCTGCTGGCGGCCCTG-3′; lower primer, 5′-CACCTTAAAATCTGCAGGCTC-3′), *SYUG* (upper primer, 5′-GGAGGACTTGAGGCCATCTG-3′; lower primer, 5′-CTCCTCTGCCACTTCCTCTTTC-3′), *HPRT* (upper primer, 5′-GACCAGTCAACAGGGGACAT-3′; lower primer, 5′-GTGTCAATTATATCTTCCACAATCAAG-3′),. Data were collected and analyzed with Roche LightCycler® 96 System Software 3.5.3 (Roche Diagnostics, Mannheim, Germany). Data were expressed as a relative amount (2^-ΔΔCT^) of a control experiment used as a calibrator.

### Breast cancer cohort

Primary breast tumors were obtained from 526 women treated at Institut Curie - Hôpital René Huguenin (Saint-Cloud, France) between 1978 and 2008. Clinical data of the patients and characteristics of the tumors are reported in Additional file 20: Table S3. All patients have given their approval for the potential use of their tumor samples for scientific purposes. This study was approved by the local ethics committee (Breast Group of Institut Curie - René Huguenin Hospital). The samples were immediately stored in liquid nitrogen until RNA extraction. A tumor sample was considered suitable for this study if the proportion of tumor cells exceeded 70%. All patients (mean age 60.9 years, range 29–91 years) met the following criteria: primary unilateral non-metastatic breast carcinoma for which complete clinicopathological data and follow-up were available; no radiotherapy or chemotherapy before surgery; and full follow-up at Institut Curie - Hôpital René Huguenin. Estrogen receptor (ER), progesterone receptor (PR), and human epidermal growth factor receptor 2 (ERBB2) statuses were determined at the protein level by biochemical methods (Dextran-coated charcoal method, enzyme immunoassay, or immunohistochemistry) and confirmed by real-time quantitative RT-PCR. The population was divided into four groups according to HR (ER and PR) and ERBB2 statuses as follows: two luminal subtypes [HR+ (ERα+ or PR+)/ERBB2+ (*n*=58)] and [HR+ (ERα+ or PR+)/ERBB2- (*n*=294)]; an ERBB2+ subtype [HR− (ERα− and PR−)/ERBB2+ (*n*=73)] and a triple-negative subtype [HR− (ERα− and PR-)/ERBB2− (*n*=101)].

### RT-qPCR (breast cancer samples)

Samples of the breast tumor cohort (526 human clinical specimens) have been analyzed in RT-qPCR. Conditions for total RNA extraction, cDNA synthesis and PCR reaction have been described elsewhere [92]. Quantitative values were obtained from the cycle number (Ct value) using ABI Prism 7900HT Sequence Detection System and PE Biosystems analysis software according to the manufacturer’s instruction (Perkin-Elmer, Waltham, MA, USA). Data from each sample were normalized on the basis of its content in *TBP* transcripts. *TBP* encoding the TATA box-binding protein (a component of the DNA-binding protein complex TFIID) was selected as an endogenous control due to the moderate level of its transcripts and the absence of known *TBP* retro-pseudogenes (retro-pseudogenes lead to co-amplification of contaminating genomic DNA and thus interfere with RT-PCR transcripts, despite the use of primers in separate exons). Results, expressed as N-fold differences in target gene expression relative to the *TBP* gene and termed “N_target_”, were determined as N_target_ = 2^ΔCtsample^, where the ΔCt value of the sample was determined by subtracting the average Ct value of target gene from the average Ct value of *TBP* gene. Primers for *NME4 (*upper primer, 5′-GGACACACCGACTCGGCTGA-3′; lower primer, 5′-GCGTGGATGACATTCCTGCTG-3′*), NME1* (upper primer, 5′-ATCAAACCAGATGGGGTCCAG-3′; lower primer, 5′-AGAAGATCTTCGGAAGCTTGCAT-3′), *CK18* (upper primer, 5′-GATGGCGAGGACTTTAATCTTGGT-3′; lower primer, 5′-GGTGGTGGTCTTTTGGATGGTT-3′*), CDH1 (*upper primer, 5′-CGCATTGCCACATACACTCTCTT-3′; lower primer, 5′-TCGGGCTTGTTGTCATTCTGAT-3′), *VIM* (upper primer, 5′-CTCCCTCTGGTTGATACCCACTC-3′; lower primer, 5′AGAAGTTTCGTTGATAACCTGTCCA-3′), and *TBP* (upper primer, 5′-TGCACAGGAGCCAAGAGTGAA-3′; lower primer, 5′-CACATCACAGCTCCCCACCA-3′), were selected with Oligo 6.0 program (National Biosciences, Plymouth, MN, USA).

### METABRIC and TCGA databases

Gene expression data were extracted from cBioPortal for Cancer Genomics (https://www.cbioportal.org/), which provides visualization, analysis, and download of large-scale cancer genomics data sets [93, 94], by specifically focusing on METABRIC (Molecular Taxonomy of Breast Cancer International Consortium) [95, 96] and TCGA (The Cancer Genome Atlas) research network database. EMT signature was calculated with the methodology defined in [97].

### Statistical analysis

Statistical analyses were performed using GraphPadPrism (version 7.00) software. The comparisons of *NME4* mRNA levels between the different subgroups of human breast tumors and the comparisons of lung metastases number between the different CTR, WT, KD clones in immunocompromised mice, were performed by the Kruskall-Wallis test followed by two by two comparison performed with the Dunn’s test. Relationships between mRNA expression of the different target genes from the human breast tumor cohort (*n*=526 human breast tumor clinical specimens) and from the TCGA databank were identified using the non-parametric Spearman’s rank correlation test (relationship between two quantitative parameters). Linear regression analysis with ANOVA test was performed to determine significance for correlations between different genes from the METABRIC databank. Survival distributions were estimated with the Kaplan-Meier method and the significance of differences between survival rates was ascertained with the log-rank test. For all other comparisons between two groups, we performed an unpaired Student’s *t* test. Differences were considered significant at confidence levels greater than 95% (*p*< 0.05).

## Supplementary Information

**Additional file 1: Fig. S1.** NDPK protein expression, kinase activity, and subcellular localization in HeLa clones. HeLa cells were stably transfected with empty vector pcDNA4TO (CTR) or constructs for expression of NDPK-D WT (WT), CL-binding deficient R90D (BD) or kinase dead H151N (KD). **A)** Immunoblot detection of NDPK-D (NME4) in extracts of the transfected HeLa cells with α-tubulin as loading control. **B)** NDP kinase activity in purified HeLa mitochondria. Values are means ± SEM (n=3). **C)** HeLa clones stably transfected with empty vector (CTR), or expressing NDPK-D WT, BD or KD mutants, showing identical labeling of mitochondrion-selective dye MitoTracker Red CMXRos (red) and immunolabeled NDPK-D (green). Mitochondrial network details are indicated by faint line boxes magnified in bold line boxes. Scale bar, 10 μm. **D)** Immunoblot detection of NDPK-A (NME1) and NDPK-B (NME2) in extracts of the transfected HeLa cells with α-tubulin as loading control.

**Additional file 2: Movie 1.** Videomicroscopy of control HeLa clones.

**Additional file 3: Movie 2.** Videomicroscopy of wild-type NDPK-D HeLa clones.

**Additional file 4: Movie 3.** Videomicroscopy of membrane-binding deficient NDPK-D mutant HeLa clones.

**Additional file 5: Movie 4.** Videomicroscopy of kinase-dead NDPK-D mutant HeLa clones.

**Additional file 6: Fig. S2.** Invasion assay of HeLa cells depleted for NDPK-A. **A)** Phase-contrast microscopy of control (scramble, Scr) and NDPK-A-depleted HeLa cells 72 h post-transfection. Note: Silenced cells are scattered as compared to control. **B)** Control siRNA and NDPK-A depleted (siNME1-1, siNME1-2) HeLa cells were tested for their ability to invade native type I collagen in a 24 h invasion assay. Data are means ± SEM (2 independent experiments). **C)** Activation status of Rac1 (Rac1-GTP) and PAK (phosphorylated PAK, pPAK) of NDPK-A depleted (siNME1-1, siNME1-2) HeLa cells as compared to total Rac1 and PAK protein, and NDPK-A protein levels. ^$$^p< 0.01. 

**Additional file 7: Fig. S3.** 14-days invasion assay of NDPK-D HeLa clones. Clones WT (left) and KD (right) are shown (for abbreviations see Fig. 1). Cells were seeded on the surface of collagen type I indicated by an arrow. Representative cross-sections of the collagen gel after a 14-day culture period stained with hematoxylin and eosin are shown (scale bar, 100 μm).

**Additional file 8: Fig. S4.** Proliferation assays of HeLa clones. **A)** Cell proliferation of HeLa clones (CTR, WT, BD, KD; for abbr. see Fig. 1) was examined between 12 and 36 h using the xCELLigence System. Proliferation rate (slope) was determined by the RTCA Software supplied with the instrument. Values are means ± SEM (n=3). **B)** Levels of proliferation markers, cyclin A, cyclin B1 and PCNA with α-tubulin as loading control were analyzed by Western blotting of HeLa clone extracts. ***p< 0.005 relative to control/empty vector (CTR); ^###^p< 0.005 relative to wild-type (WT).

**Additional file 9: Fig. S5.** Pro-invasive signaling pathways in HeLa clones. **A, B)** Mutant NDPK-D cells were tested for their ability to invade native type I collagen in the presence of pharmacological inhibitors of the PI3K (GSK2126458), Src (MA475271), p38 (SB203580), JNK (SP600125) signaling pathways **(A)**, and epidermal growth factor receptor (EGFR) (lapatinib) **(B)**, all at two different concentrations (1 and 10 μM). **C, D)** Activation of the EGFR signaling pathway after 10 nM EGF stimulation analyzed by immunoblotting with phospho-specific and total protein antibodies as indicated, with α-tubulin as loading control; **(C)** clones CTR, WT, BD; **(D)** clones CTR, WT, KD. Note: Activation of the EGF pathway is seen by phosphorylation of EGFR (at Tyr^1080^, activatory), ERK1/2, AKT, and GSK3β (at Ser^9^, inhibitory). ^$$^p< 0.01 and ^$$$^p< 0.005. For clone abbreviations see Fig. 1.

**Additional file 10: Table S1.** Differently expressed proteins in HeLa clones expressing the mutant and the wild type NDPK-D. The full protein names are from the UniProt database. Accession number are from UniProt (Acc._HUMAN) and SwissProt databases. The one-way analysis of variance (ANOVA) test, followed by a Tukey’s multiple comparison test, was used to determine protein spots significantly different between analyses. *p*-values were calculated across pairwise comparisons (clones KD vs WT, BD vs WT and CTR vs WT) and considered significant when < 0.05. Proteins were ordered following the fold changes in the KD vs WT comparison. * Two identifications for the same spot. Bold values, fold change statistically significant (*p*< 0.05) and ≥1.3. Italic values, fold change not statistically valid (*p* > 0.05) or ≤1.3. § Proteins reported to present a mitochondrial localization (UniProt annotation) are indicated by M.

**Additional file 11: Table S2.** Mass spectrometry identification data for spots of interest. For each protein identified in a spot of interest, the table gives: the protein full name, the accession number from UniProt database, the gene name, the Mascot scores, the number of unique peptides, the calculated emPAI (Exponentially Modified Protein Abundance Index) value, the experimental and theoretical pI (isoelectric point) and molecular weight (Mw) and the sequence coverage (%) (see: http://www.matrixscience.com). * Two identifications for the same spot.

**Additional file 12: Fig. S6.** Immunoblot and RTqPCR analysis of candidates identified by proteomics. **A)** Protein levels of five candidates found overexpressed in KD vs. WT by 2D-DIGE proteomics (fascin, γ-synuclein, ISG15, S100A4 and tubulin-βΙΙA) were analyzed by immunoblotting. Two independent clones of each type (indicated as 1 and 2) with two different cultures for each clone were analyzed; α-tubulin is given as loading control. The arrow indicates the correct ISG15 band. **B-F)** mRNA levels in the WT and KD clones were measured by RTqPCR: fascin **(B)**, γ-synuclein **(C)**, ISG15 **(D)**, S100A4 **(E)**, and N-cadherin involved in cell-cell contacts **(F)**. Data are means ± SEM (n=3). ^###^p< 0.005 relative to WT. For clone abbreviations see Fig. 1.

**Additional file 13: Fig. S7.** Mitochondrial network structure in live-stained HeLa clones. HeLa cells harboring empty vector control (CTR) or expressing wild-type NDPK-D (WT) or mutant NDPK-D (BD, KD) were labeled with 100 nM Mitotracker Green. Representative confocal images are shown together with a 2.7-fold magnified detail to the right. Scale bar, 20 μm.

**Additional file 14: Fig. S8.** NME4 expression is reduced in human breast tumor cell lines with the triple-negative phenotype. *NME4* mRNA levels were measured by RT–qPCR in normal-like human breast cell lines, in hormone receptor-positive (HR+) human breast tumor cell lines, and in triple-negative (TN) human breast tumor cell lines. Each data point represents one cell line. Three independent analyses were performed for each cell line. Data are expressed as means ± SEM. ***p< 0.001, **p< 0.01.

**Additional file 15: Fig. S9.** NDPK-D protein expression and mitochondrial localization in MDA-MB-231 clones. **A)** Immunoblot detection of NDPK-D from MDA-MB-231 cells stably transfected with empty pcDNA4TO (CTR) or constructs for expression of NDPK-D WT, BD or KD. Alpha-tubulin was used as loading control. **B)** MDA-MB-231 clones stably transfected with empty vector (CTR), or expressing NDPK-D WT or mutants BD or KD, showing labeling of mitochondrion-selective dye MitoTracker Red CMXRos (red) and immunolabeled NDPK-D (green). Mitochondrial network details are indicated by faint line boxes magnified in bold line boxes. Scale bar, 10 μm.

**Additional file 16: Fig. S10.** NDPK-D protein expression of ZR75-1 cells. Immunoblot detection of NDPK-D from ZR75-1 cells depleted of NDPK-D by siRNA. Alpha-tubulin was used as loading control.

**Additional file 17: Fig. S11.** MMP activity of ZR75-1 cells depleted for NDPK-D. **A)** Left panel, representative images of MMP activity by gelatin degradation zymography; the degradation bands of MMP9 are detected at 92 KDa. Right panel, representative Coomassie brilliant blue (CBB) of samples run simultaneously is shown as a loading control. Two different siRNA targeting NDPK-D were used. **B)** Bar graphs represent the densitometric and statistical analyses of the bands obtained by gelatin zymography shown for MMP9 of five independent biological replicates. Data show means ± SEM (*n*=5). *p< 0.05 relative to scramble control (Scr).

**Additional file 18: Fig. S12.** Mitochondrial potential and mitochondrial stress of ZR75-1 cells depleted for NDPK-D. **A)** Mitochondrial membrane potential was measured by staining ZR75-1 cells depleted for NDPK-D with 200 nM TMRE and the percentage of fluorescence intensity of three independent biological replicates was plotted. Data show means ± SEM of three independent biological replicates imaged. *p< 0.05 relative to scramble control (Scr). **B)** Mitochondrial oxidative state was determined by staining the same cell lines with 5 μM MitoSOX^TM^ and the percentage of fluorescence intensity of three independent biological replicates was plotted. Data show means ± SEM of three independent biological replicates imaged. *p< 0.05 relative to scramble control (Scr).

**Additional file 19: Fig. S13.** Hematoxylin and eosin labeling of lung metastases after injection of HeLa clones. Representative H&E labeling of lung metastases after i.v injection of CTR, WT, and KD HeLa clones. Scale bar, 5 mm.

**Additional file 20: Table S3.** Characteristics of the 526 human breast tumor cohort.

**Additional file 21: Fig. S14.** Association between *NME4*, *NME1* and markers of EMT in human breast tumors. *NME4* and *NME1* status in the cohort of 526 human breast tumor clinical samples: mRNA correlation between *NME4* and *CDH1* **(A)**, *NME4* and *KRT18* **(B),** *NME1* and *CDH1* (**C),** *NME1* and *KRT18* **(D)**, *NME1* and *VIM* **(E).**

**Additional file 22: Fig. S15.** Association between *NME4* and regulators of EMT in the human breast tumor METABRIC database. The database (1904 human breast tumors) was retrieved for mRNA expression of *NME4* and EMT markers and their correlation analyzed: epithelial markers, *CDH1* and *KRT18* **(A)**; mesenchymal markers, *CDH2* and *VIM* **(B)**; EMT drivers, *ZEB1*, *ZEB2* **(C)**, *SNAI1*, *SNAI2* **(D)**, *TWIST1*, *TWIST2* **(E)**, and the EMT score **(F)**. Correlation coefficients are summarized in **(G)**.

**Additional file 23: Table S4.** Association between *NME4* and EMT and tumor invasion marker expression in breast tumors. The relationship between *NME4* expression and several key players of EMT and tumor invasion was studied in human breast tumors from the TCGA database.

**Additional file 24: Fig. S16.** Association between *NME4* and markers of EMT and tumor invasion in the human breast tumor TCGA database. The database was retrieved for mRNA expression of *NME4* and EMT (*KRT18, CDH2, ZEB2, CTNNB1, CLDN3*) and tumor invasion (*MMP7, ADAM17, ROCK2, CFL2, MYO5A*) markers and their correlation analyzed (see Additional file 23: Table S4 ).

**Additional file 25: Table S5.** Association between *NME4*, EMT and tumor invasion marker expression in cervix tumors. The relationship between *NME4* expression and several key players of EMT and tumor invasion was studied in human cervix tumors from the TCGA database.

**Additional file 26.** Images of the full immunoblots.

## Data Availability

All data generated or analyzed during this study are included in this published article and its supplementary information files. Gene expression data were publicly extracted from cBioPortal for Cancer Genomics (https://www.cbioportal.org/) by specifically focusing on METABRIC and TCGA research network database.
