# Supplementary figures and images for: The mitochondrially-localized nucleoside diphosphate kinase D (NME4) is a novel metastasis suppressor
Source: BMC Biol. 2021 Oct 21;19:228. doi: 10.1186/s12915-021-01155-5 (PMC8529772; doi:10.1186/s12915-021-01155-5)

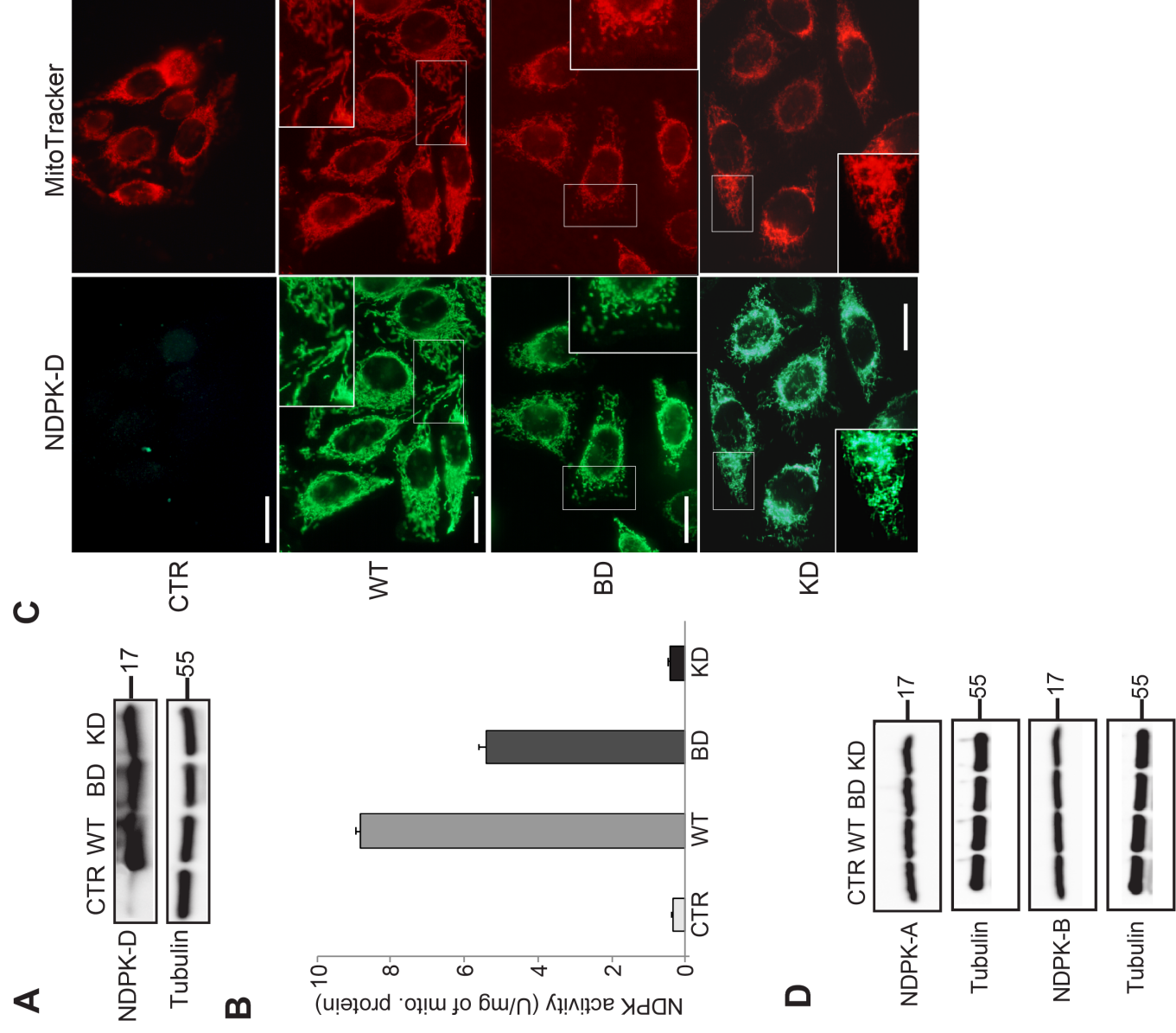

Supplement: Supplementary file 1 — Additional file 1: Fig. S1. NDPK protein expression, kinase activity, and subcellular localization in HeLa clones. HeLa cells were stably transfected with empty vector pcDNA4TO (CTR) or constructs for expression of NDPK-D WT (WT), CL-binding deficient R90D (BD) or kinase dead H151N (KD). A) Immunoblot detection of NDPK-D (NME4) in extracts of the transfected HeLa cells with α-tubulin as loading control. B) NDP kinase activity in purified HeLa mitochondria. Values are means ± SEM (n=3). C) HeLa clones stably transfected with empty vector (CTR), or expressing NDPK-D WT, BD or KD mutants, showing identical labeling of mitochondrion-selective dye MitoTracker Red CMXRos (red) and immunolabeled NDPK-D (green). Mitochondrial network details are indicated by faint line boxes magnified in bold line boxes. Scale bar, 10 μm. D) Immunoblot detection of NDPK-A (NME1) and NDPK-B (NME2) in extracts of the transfected HeLa cells with α-tubulin as loading control. [file 12915_2021_1155_MOESM1_ESM.pdf]

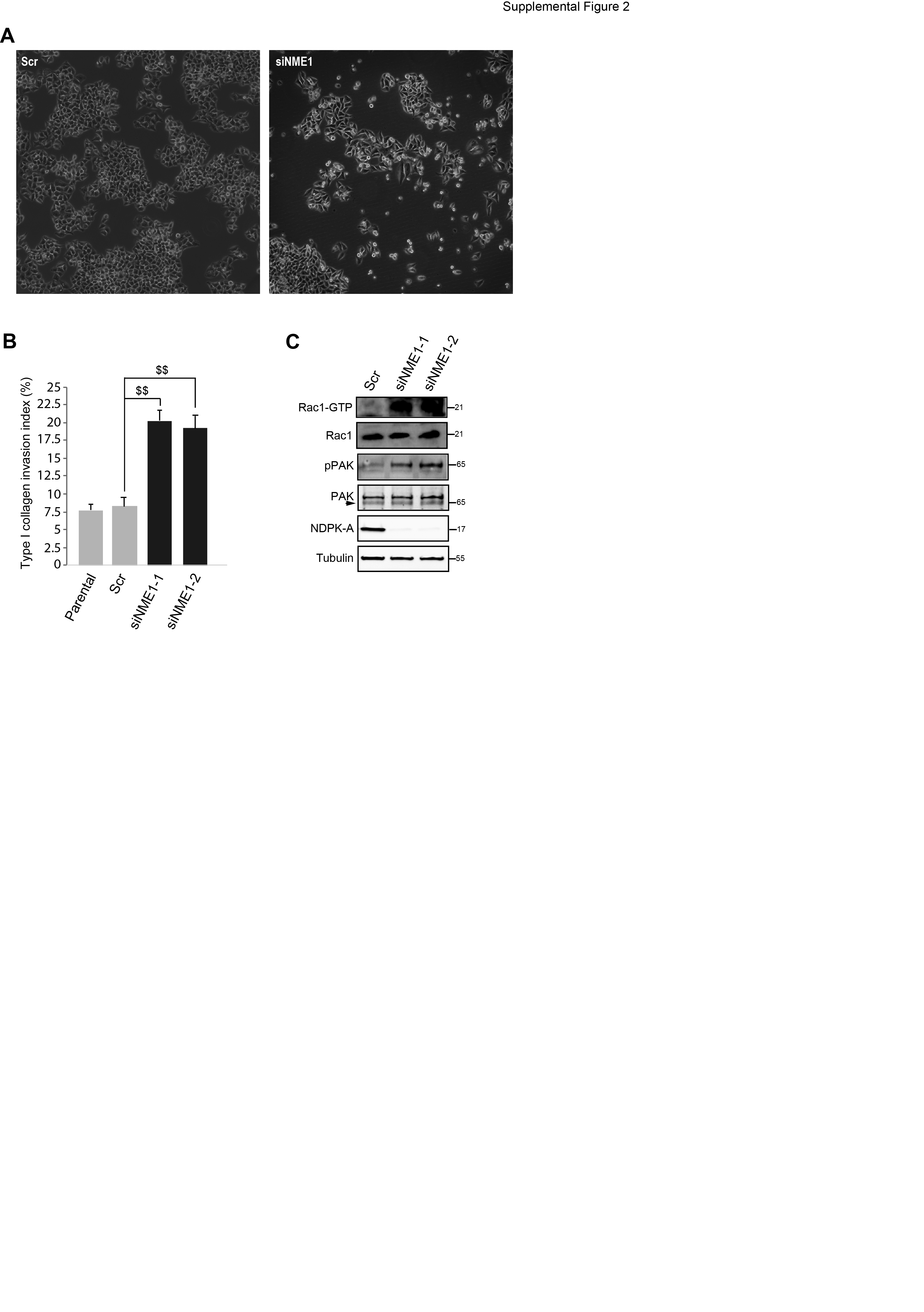

Supplement: Supplementary file 6 — Additional file 6: Fig. S2. Invasion assay of HeLa cells depleted for NDPK-A. A) Phase-contrast microscopy of control (scramble, Scr) and NDPK-A-depleted HeLa cells 72 h post-transfection. Note: Silenced cells are scattered as compared to control. B) Control siRNA and NDPK-A depleted (siNME1-1, siNME1-2) HeLa cells were tested for their ability to invade native type I collagen in a 24 h invasion assay. Data are means ± SEM (2 independent experiments). C) Activation status of Rac1 (Rac1-GTP) and PAK (phosphorylated PAK, pPAK) of NDPK-A depleted (siNME1-1, siNME1-2) HeLa cells as compared to total Rac1 and PAK protein, and NDPK-A protein levels. $$p< 0.01. [file 12915_2021_1155_MOESM6_ESM.tif]

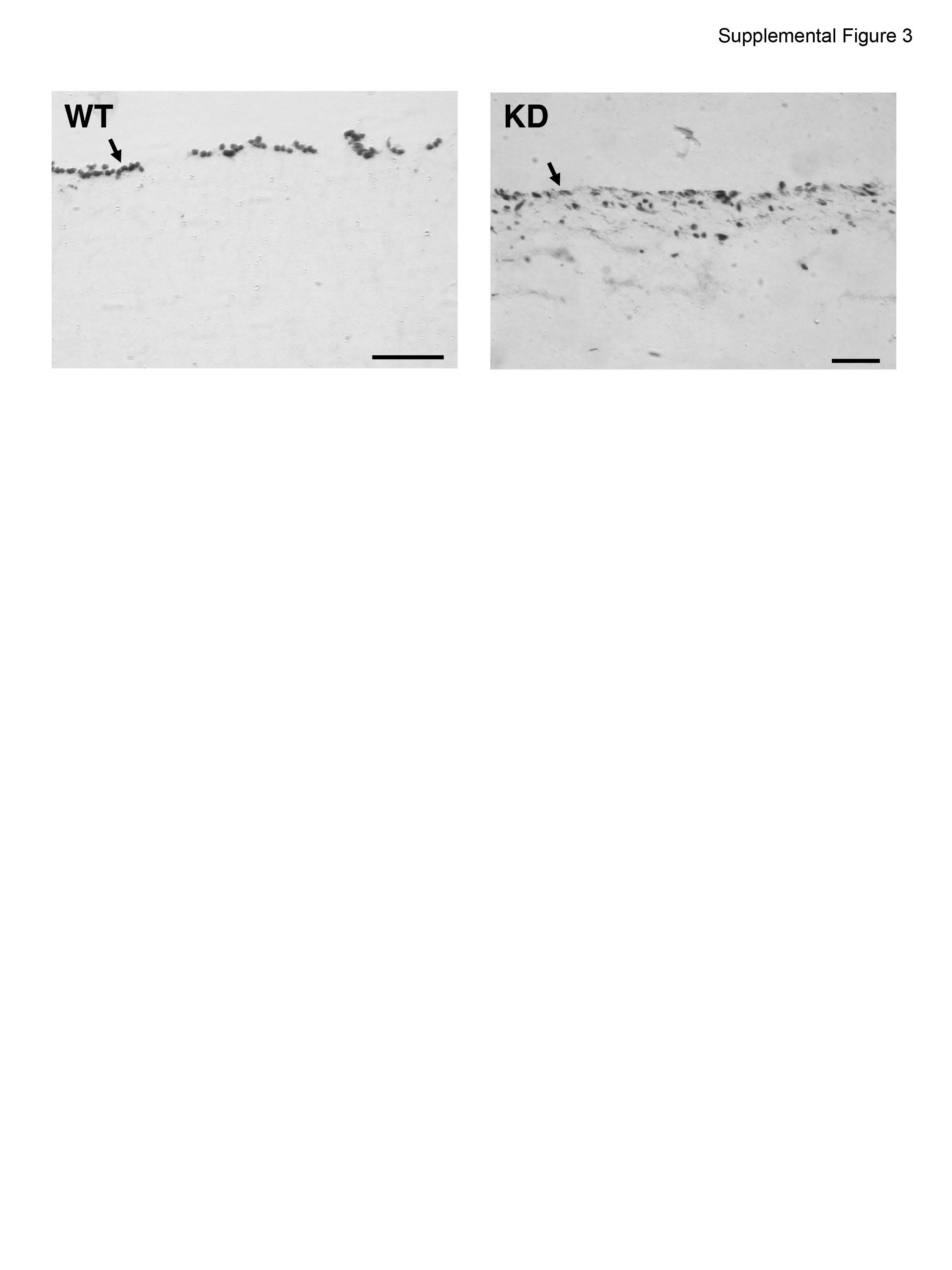

Supplement: Supplementary file 7 — Additional file 7: Fig. S3. 14-days invasion assay of NDPK-D HeLa clones. Clones WT (left) and KD (right) are shown (for abbreviations see Fig. 1). Cells were seeded on the surface of collagen type I indicated by an arrow. Representative cross-sections of the collagen gel after a 14-day culture period stained with hematoxylin and eosin are shown (scale bar, 100 μm). [file 12915_2021_1155_MOESM7_ESM.tif]

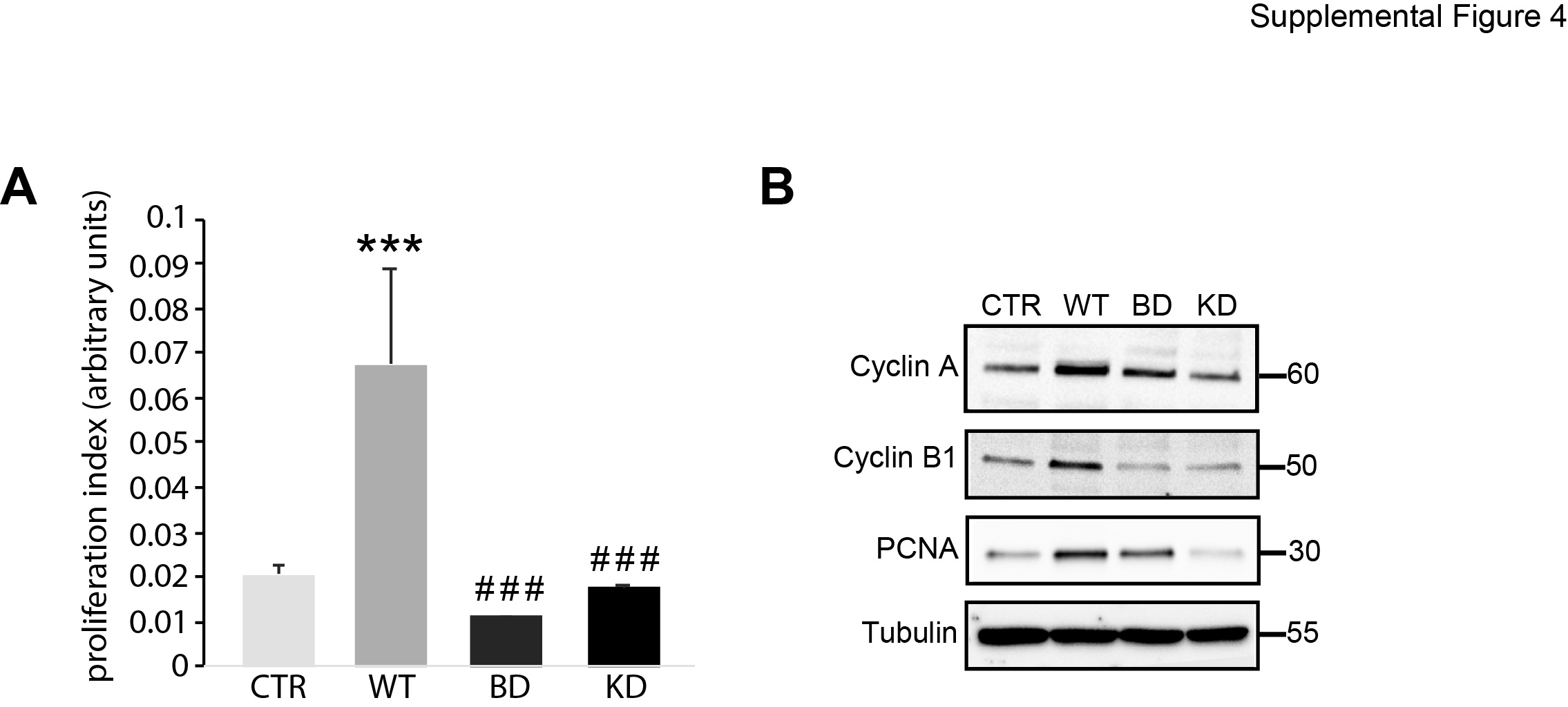

Supplement: Supplementary file 8 — Additional file 8: Fig. S4. Proliferation assays of HeLa clones. A) Cell proliferation of HeLa clones (CTR, WT, BD, KD; for abbr. see Fig. 1) was examined between 12 and 36 h using the xCELLigence System. Proliferation rate (slope) was determined by the RTCA Software supplied with the instrument. Values are means ± SEM (n=3). B) Levels of proliferation markers, cyclin A, cyclin B1 and PCNA with α-tubulin as loading control were analyzed by Western blotting of HeLa clone extracts. ***p< 0.005 relative to control/empty vector (CTR); ###p< 0.005 relative to wild-type (WT). [file 12915_2021_1155_MOESM8_ESM.tif]

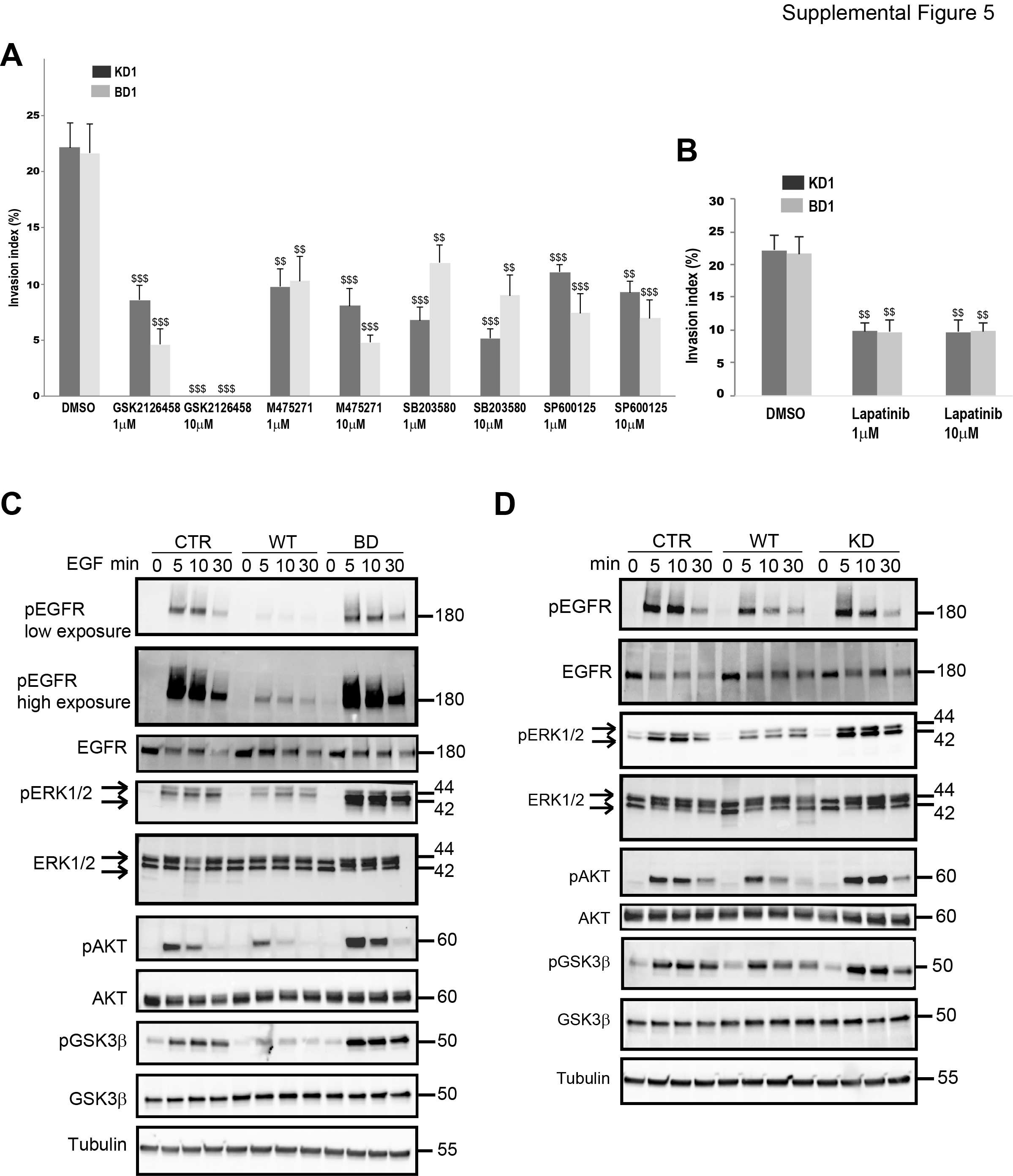

Supplement: Supplementary file 9 — Additional file 9: Fig. S5. Pro-invasive signaling pathways in HeLa clones. A, B) Mutant NDPK-D cells were tested for their ability to invade native type I collagen in the presence of pharmacological inhibitors of the PI3K (GSK2126458), Src (MA475271), p38 (SB203580), JNK (SP600125) signaling pathways (A), and epidermal growth factor receptor (EGFR) (lapatinib) (B), all at two different concentrations (1 and 10 μM). C, D) Activation of the EGFR signaling pathway after 10 nM EGF stimulation analyzed by immunoblotting with phospho-specific and total protein antibodies as indicated, with α-tubulin as loading control; (C) clones CTR, WT, BD; (D) clones CTR, WT, KD. Note: Activation of the EGF pathway is seen by phosphorylation of EGFR (at Tyr1080, activatory), ERK1/2, AKT, and GSK3β (at Ser9, inhibitory). $$p< 0.01 and $$$p< 0.005. For clone abbreviations see Fig. 1. [file 12915_2021_1155_MOESM9_ESM.tif]

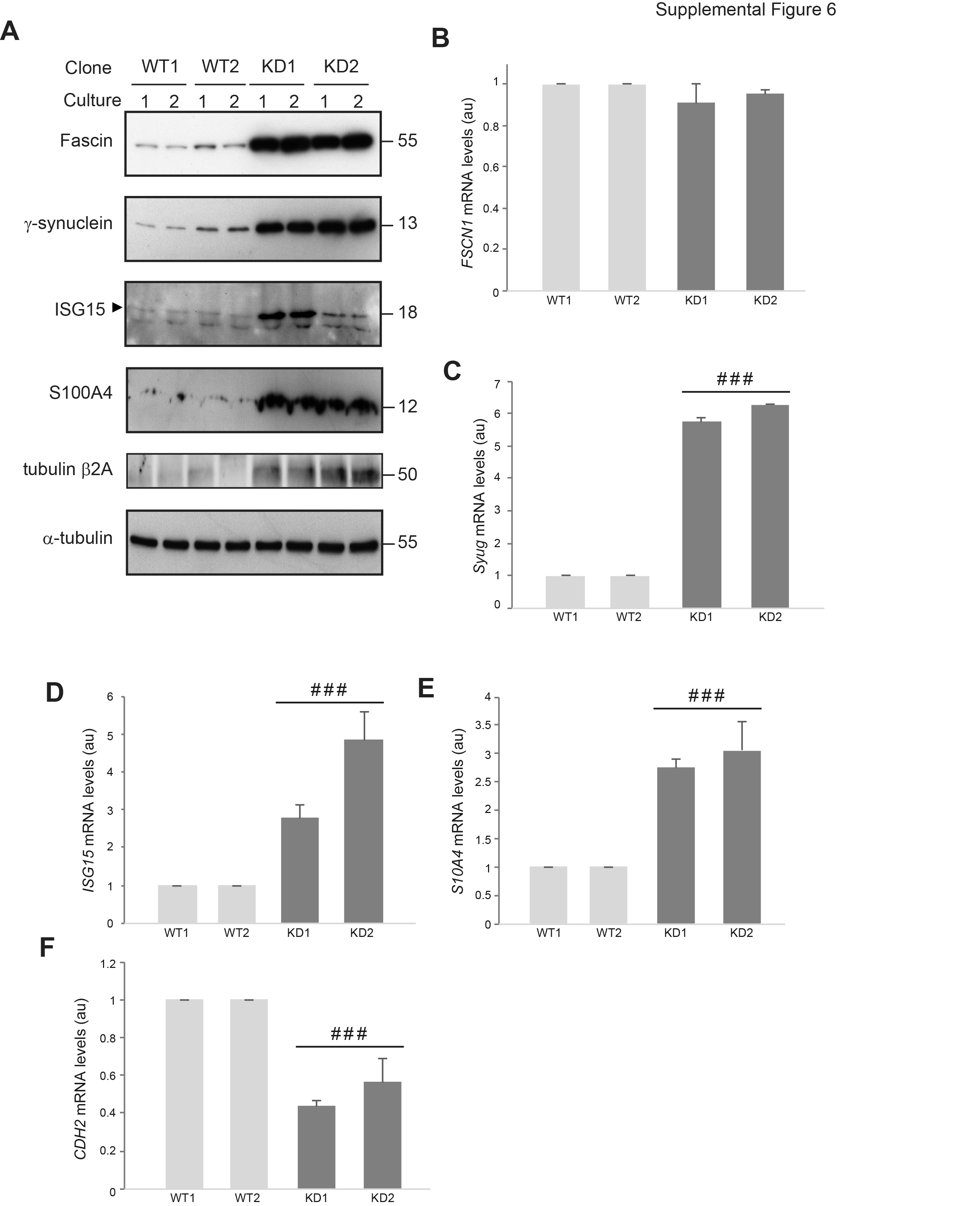

Supplement: Supplementary file 12 — Additional file 12: Fig. S6. Immunoblot and RTqPCR analysis of candidates identified by proteomics. A) Protein levels of five candidates found overexpressed in KD vs. WT by 2D-DIGE proteomics (fascin, γ-synuclein, ISG15, S100A4 and tubulin-βΙΙA) were analyzed by immunoblotting. Two independent clones of each type (indicated as 1 and 2) with two different cultures for each clone were analyzed; α-tubulin is given as loading control. The arrow indicates the correct ISG15 band. B-F) mRNA levels in the WT and KD clones were measured by RTqPCR: fascin (B), γ-synuclein (C), ISG15 (D), S100A4 (E), and N-cadherin involved in cell-cell contacts (F). Data are means ± SEM (n=3). ###p< 0.005 relative to WT. For clone abbreviations see Fig. 1. [file 12915_2021_1155_MOESM12_ESM.tif]

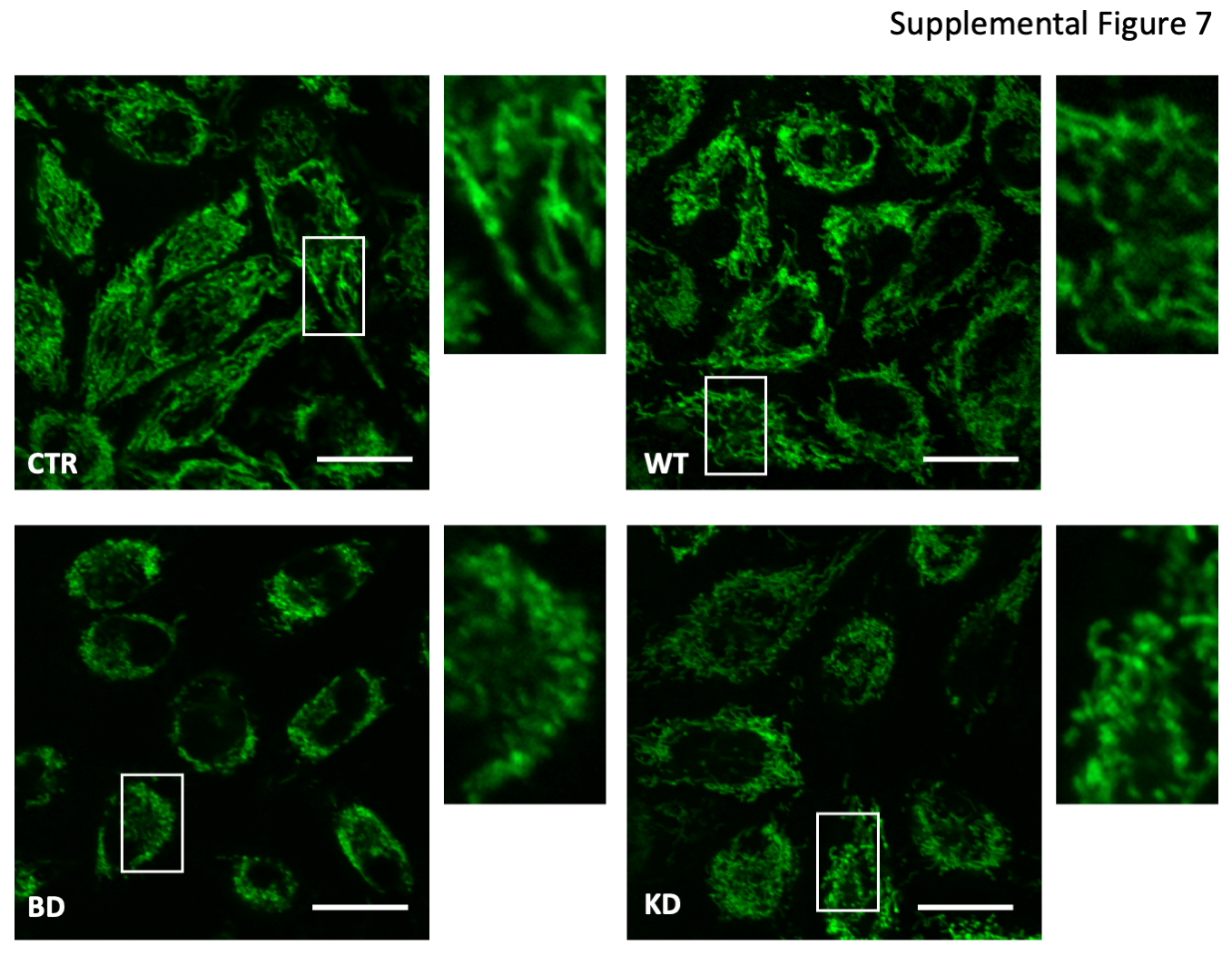

Supplement: Supplementary file 13 — Additional file 13: Fig. S7. Mitochondrial network structure in live-stained HeLa clones. HeLa cells harboring empty vector control (CTR) or expressing wild-type NDPK-D (WT) or mutant NDPK-D (BD, KD) were labeled with 100 nM Mitotracker Green. Representative confocal images are shown together with a 2.7-fold magnified detail to the right. Scale bar, 20 μm. [file 12915_2021_1155_MOESM13_ESM.tiff]

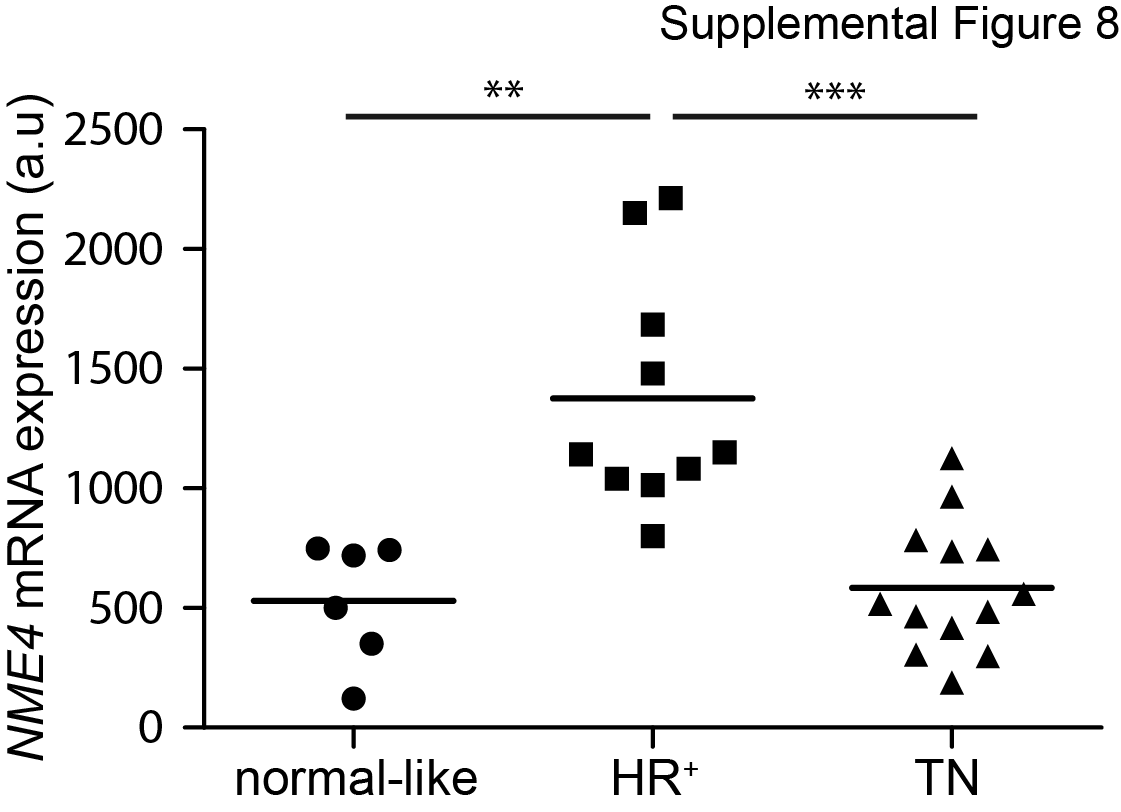

Supplement: Supplementary file 14 — Additional file 14: Fig. S8. NME4 expression is reduced in human breast tumor cell lines with the triple-negative phenotype. NME4 mRNA levels were measured by RT–qPCR in normal-like human breast cell lines, in hormone receptor-positive (HR+) human breast tumor cell lines, and in triple-negative (TN) human breast tumor cell lines. Each data point represents one cell line. Three independent analyses were performed for each cell line. Data are expressed as means ± SEM. ***p< 0.001, **p< 0.01. [file 12915_2021_1155_MOESM14_ESM.tif]

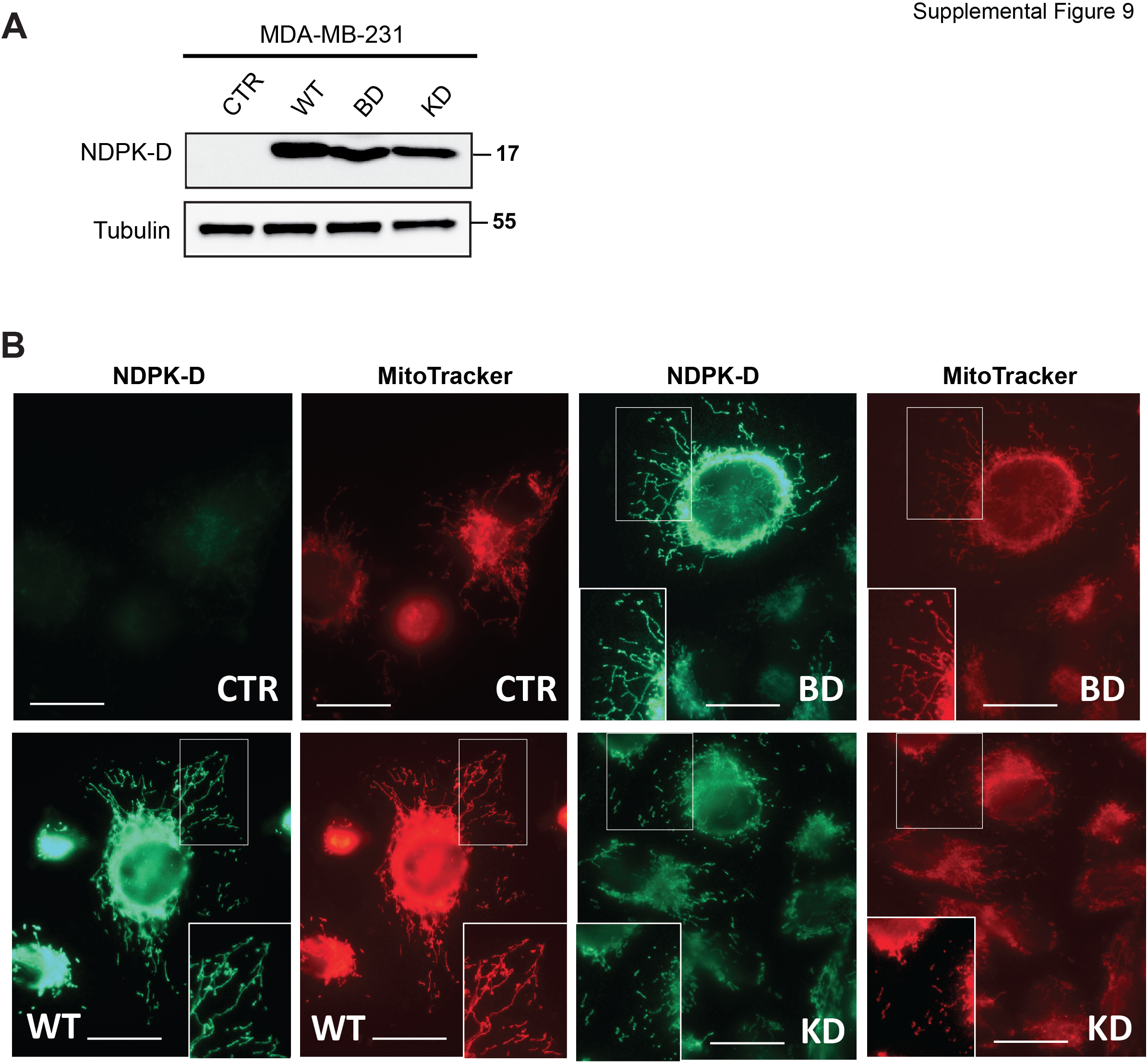

Supplement: Supplementary file 15 — Additional file 15: Fig. S9. NDPK-D protein expression and mitochondrial localization in MDA-MB-231 clones. A) Immunoblot detection of NDPK-D from MDA-MB-231 cells stably transfected with empty pcDNA4TO (CTR) or constructs for expression of NDPK-D WT, BD or KD. Alpha-tubulin was used as loading control. B) MDA-MB-231 clones stably transfected with empty vector (CTR), or expressing NDPK-D WT or mutants BD or KD, showing labeling of mitochondrion-selective dye MitoTracker Red CMXRos (red) and immunolabeled NDPK-D (green). Mitochondrial network details are indicated by faint line boxes magnified in bold line boxes. Scale bar, 10 μm. [file 12915_2021_1155_MOESM15_ESM.tif]

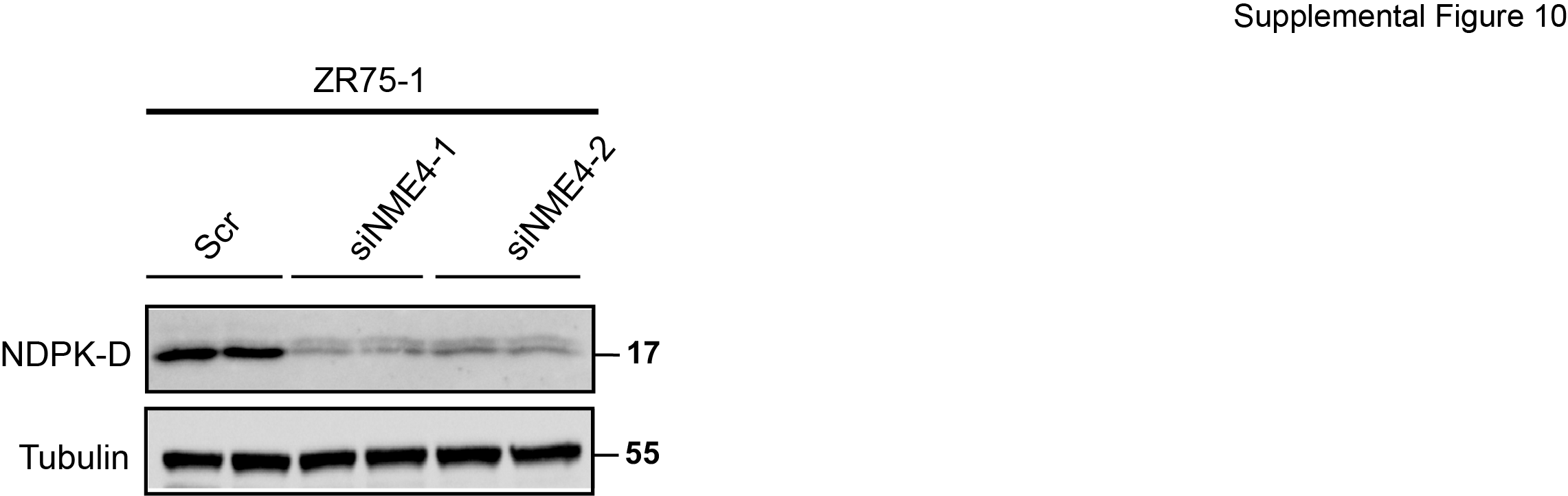

Supplement: Supplementary file 16 — Additional file 16: Fig. S10. NDPK-D protein expression of ZR75-1 cells. Immunoblot detection of NDPK-D from ZR75-1 cells depleted of NDPK-D by siRNA. Alpha-tubulin was used as loading control. [file 12915_2021_1155_MOESM16_ESM.tif]

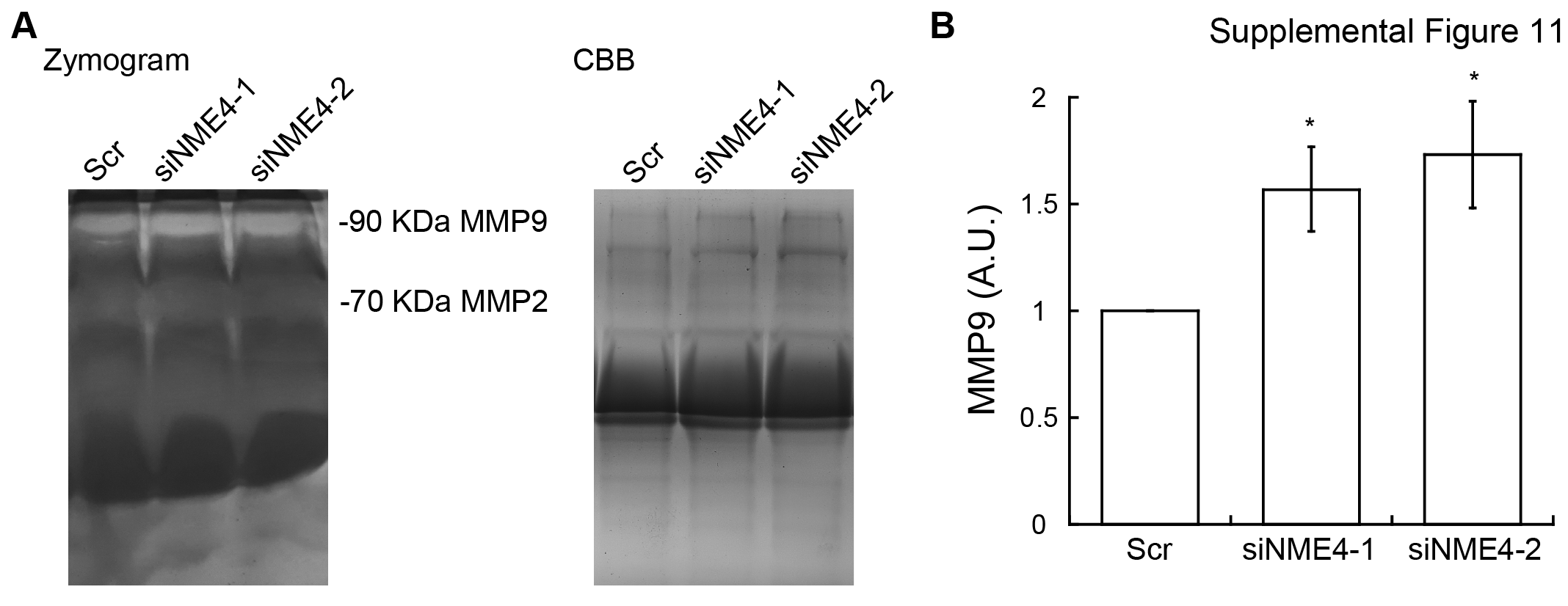

Supplement: Supplementary file 17 — Additional file 17: Fig. S11. MMP activity of ZR75-1 cells depleted for NDPK-D. A) Left panel, representative images of MMP activity by gelatin degradation zymography; the degradation bands of MMP9 are detected at 92 KDa. Right panel, representative Coomassie brilliant blue (CBB) of samples run simultaneously is shown as a loading control. Two different siRNA targeting NDPK-D were used. B) Bar graphs represent the densitometric and statistical analyses of the bands obtained by gelatin zymography shown for MMP9 of five independent biological replicates. Data show means ± SEM (n=5). *p< 0.05 relative to scramble control (Scr). [file 12915_2021_1155_MOESM17_ESM.tif]

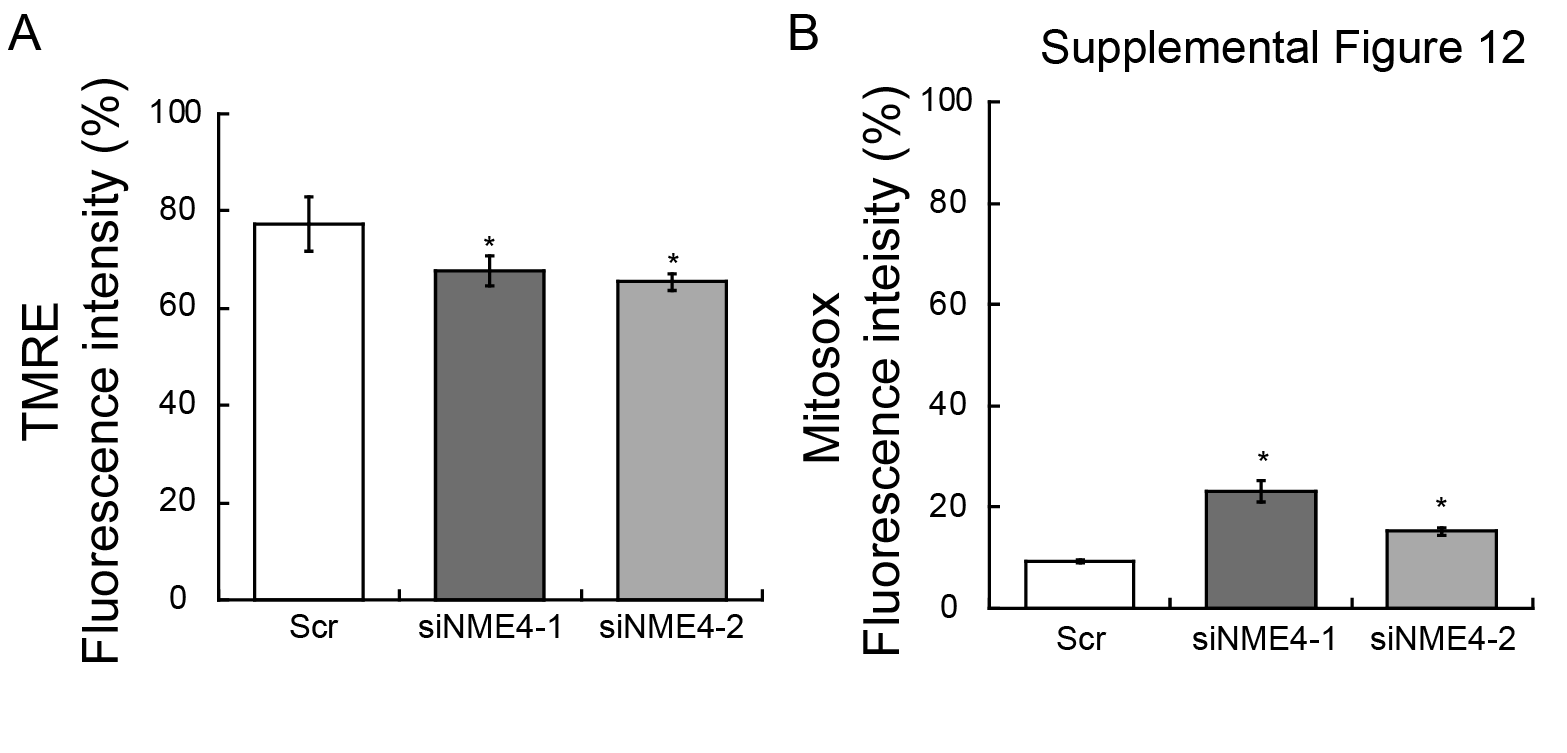

Supplement: Supplementary file 18 — Additional file 18: Fig. S12. Mitochondrial potential and mitochondrial stress of ZR75-1 cells depleted for NDPK-D. A) Mitochondrial membrane potential was measured by staining ZR75-1 cells depleted for NDPK-D with 200 nM TMRE and the percentage of fluorescence intensity of three independent biological replicates was plotted. Data show means ± SEM of three independent biological replicates imaged. *p< 0.05 relative to scramble control (Scr). B) Mitochondrial oxidative state was determined by staining the same cell lines with 5 μM MitoSOXTM and the percentage of fluorescence intensity of three independent biological replicates was plotted. Data show means ± SEM of three independent biological replicates imaged. *p< 0.05 relative to scramble control (Scr). [file 12915_2021_1155_MOESM18_ESM.tif]

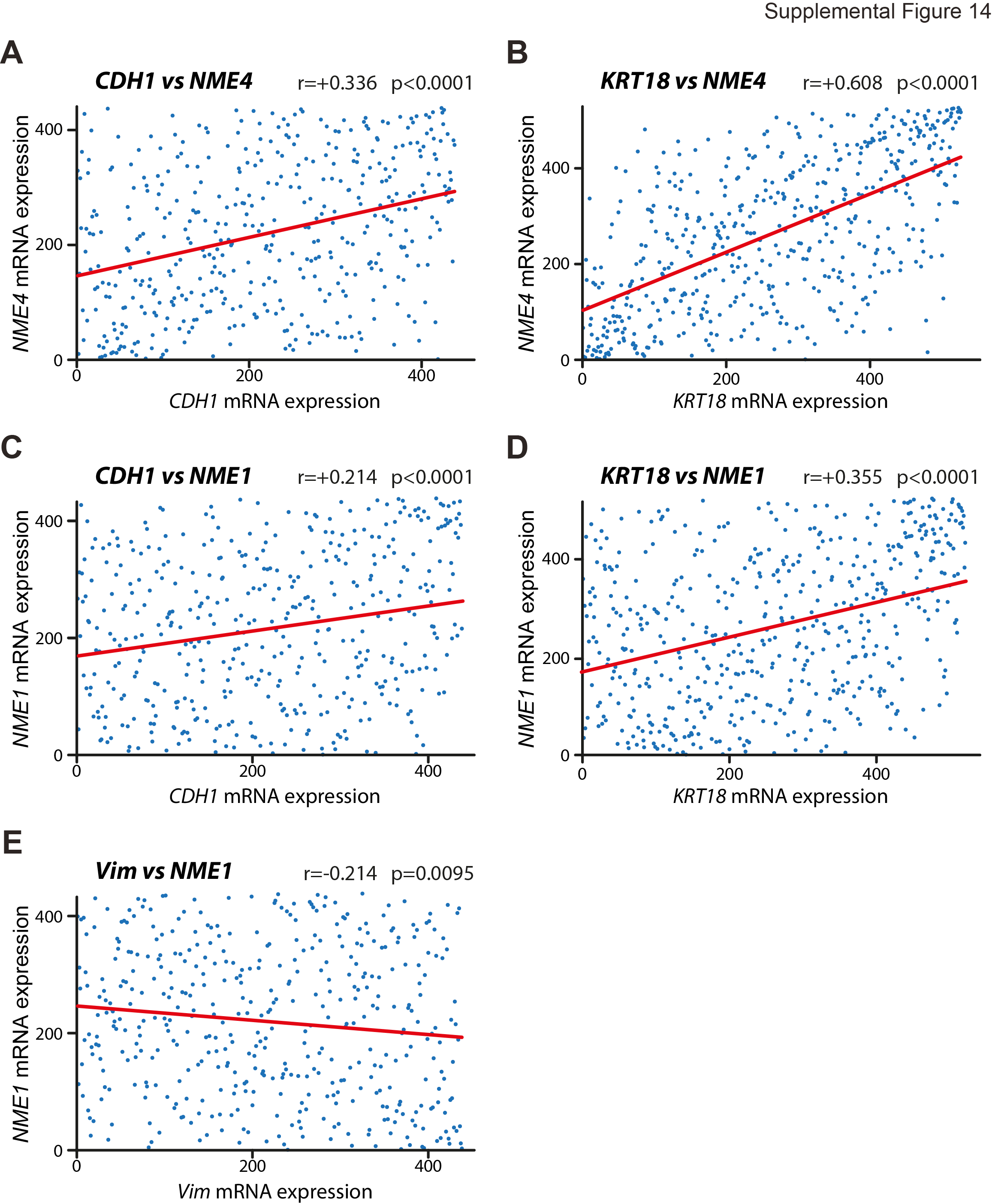

Supplement: Supplementary file 21 — Additional file 21: Fig. S14. Association between NME4, NME1 and markers of EMT in human breast tumors. NME4 and NME1 status in the cohort of 526 human breast tumor clinical samples: mRNA correlation between NME4 and CDH1 (A), NME4 and KRT18 (B), NME1 and CDH1 (C), NME1 and KRT18 (D), NME1 and VIM (E). [file 12915_2021_1155_MOESM21_ESM.tif]

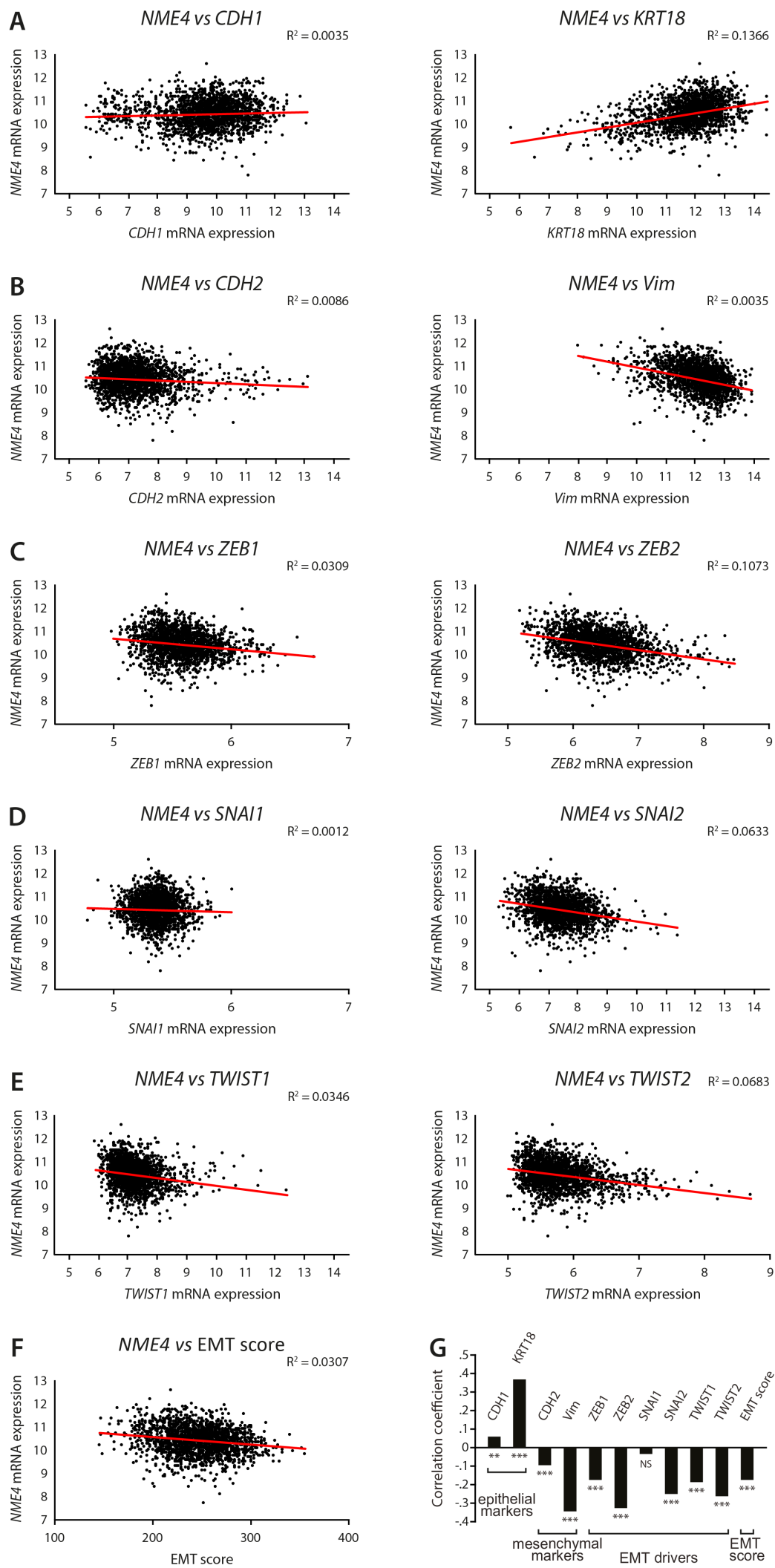

Supplement: Supplementary file 22 — Additional file 22: Fig. S15. Association between NME4 and regulators of EMT in the human breast tumor METABRIC database. The database (1904 human breast tumors) was retrieved for mRNA expression of NME4 and EMT markers and their correlation analyzed: epithelial markers, CDH1 and KRT18 (A); mesenchymal markers, CDH2 and VIM (B); EMT drivers, ZEB1, ZEB2 (C), SNAI1, SNAI2 (D), TWIST1, TWIST2 (E), and the EMT score (F). Correlation coefficients are summarized in (G). [file 12915_2021_1155_MOESM22_ESM.pdf]

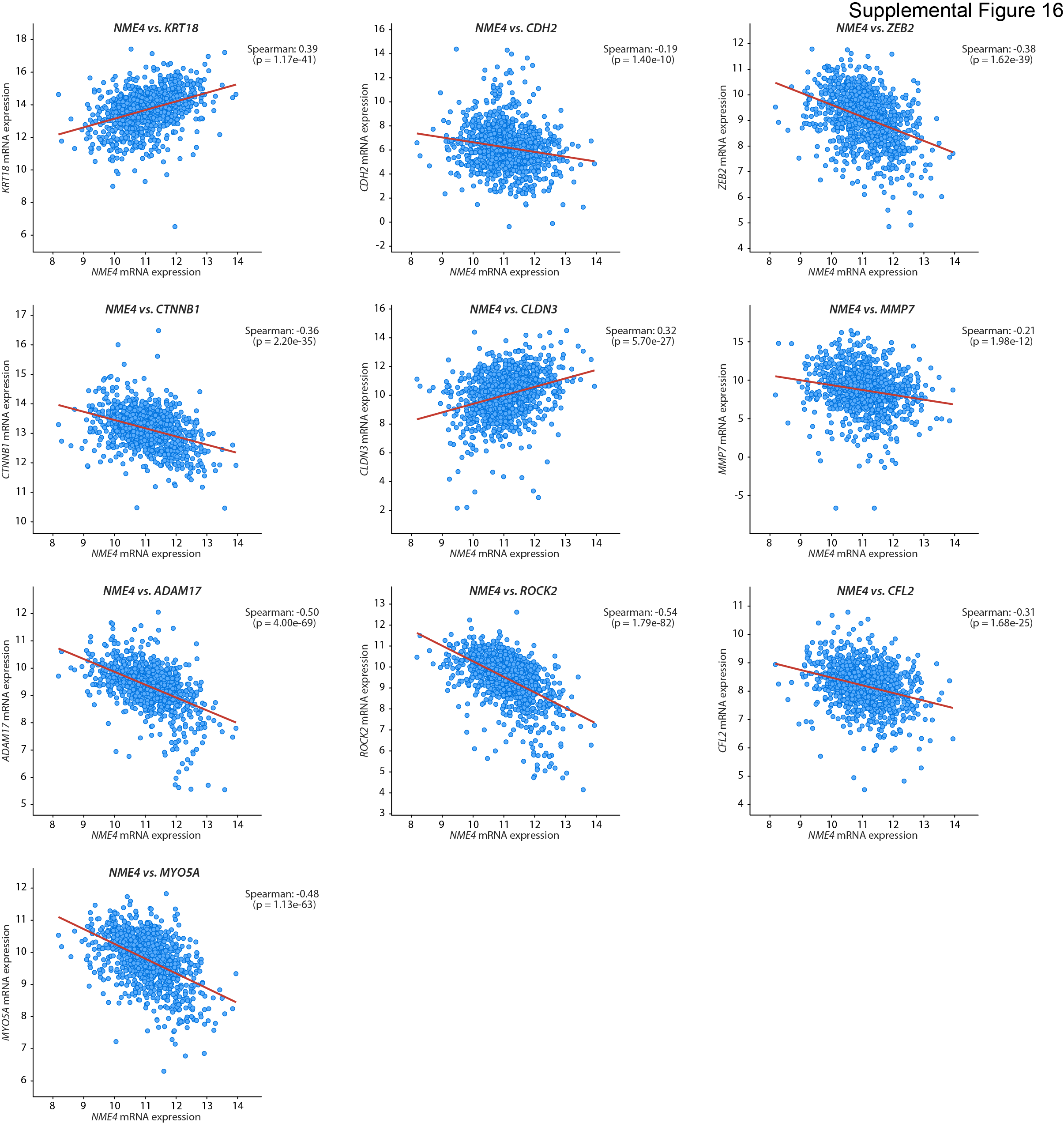

Supplement: Supplementary file 24 — Additional file 24: Fig. S16. Association between NME4 and markers of EMT and tumor invasion in the human breast tumor TCGA database. The database was retrieved for mRNA expression of NME4 and EMT (KRT18, CDH2, ZEB2, CTNNB1, CLDN3) and tumor invasion (MMP7, ADAM17, ROCK2, CFL2, MYO5A) markers and their correlation analyzed (see Additional file 23: Table S4 ). [file 12915_2021_1155_MOESM24_ESM.tif]

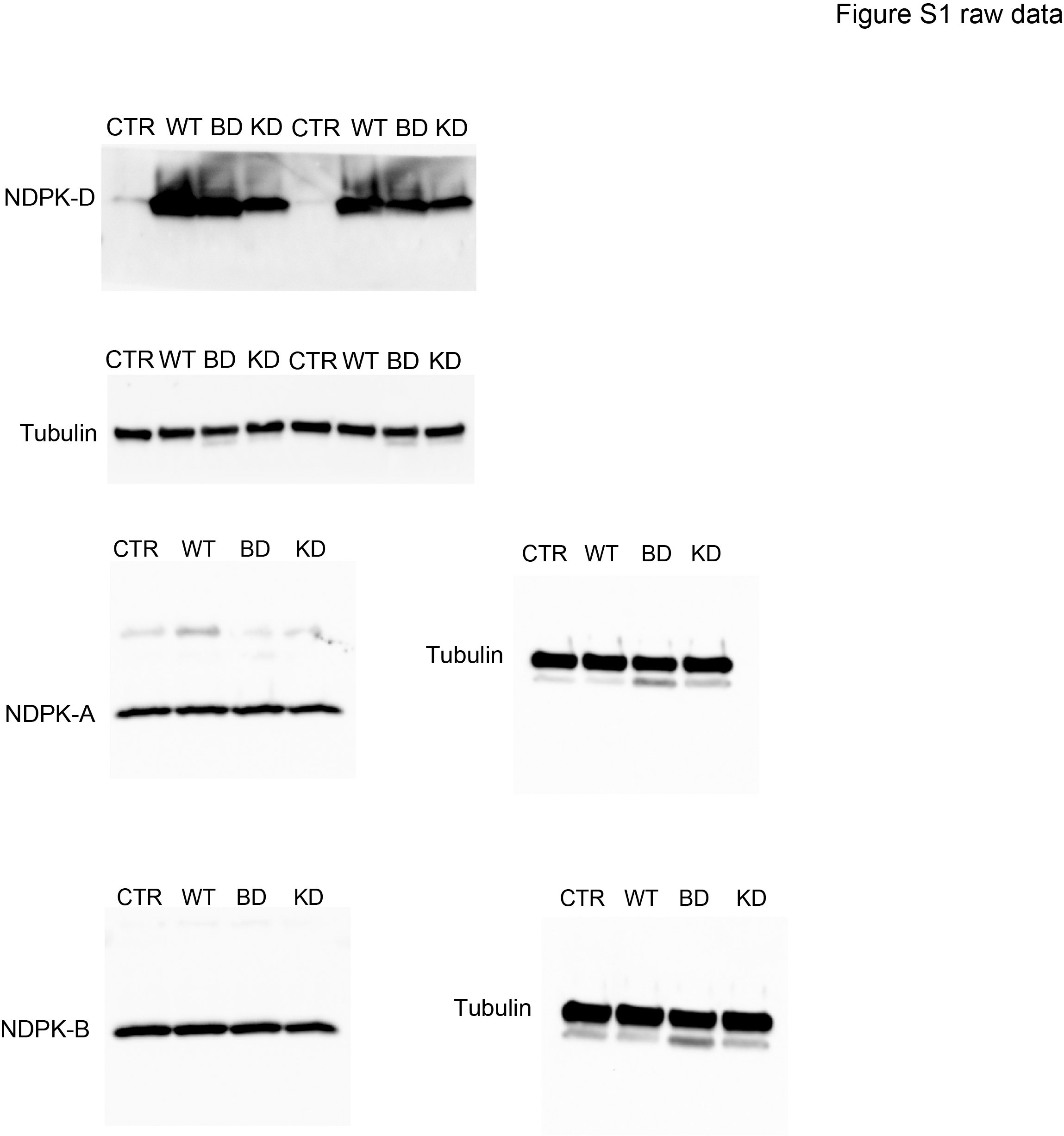

Supplement: Supplementary file 26 — Additional file 26. Images of the full immunoblots. [file 12915_2021_1155_MOESM26_ESM.zip › 26/Additional file 1, Fig. S1.tif]

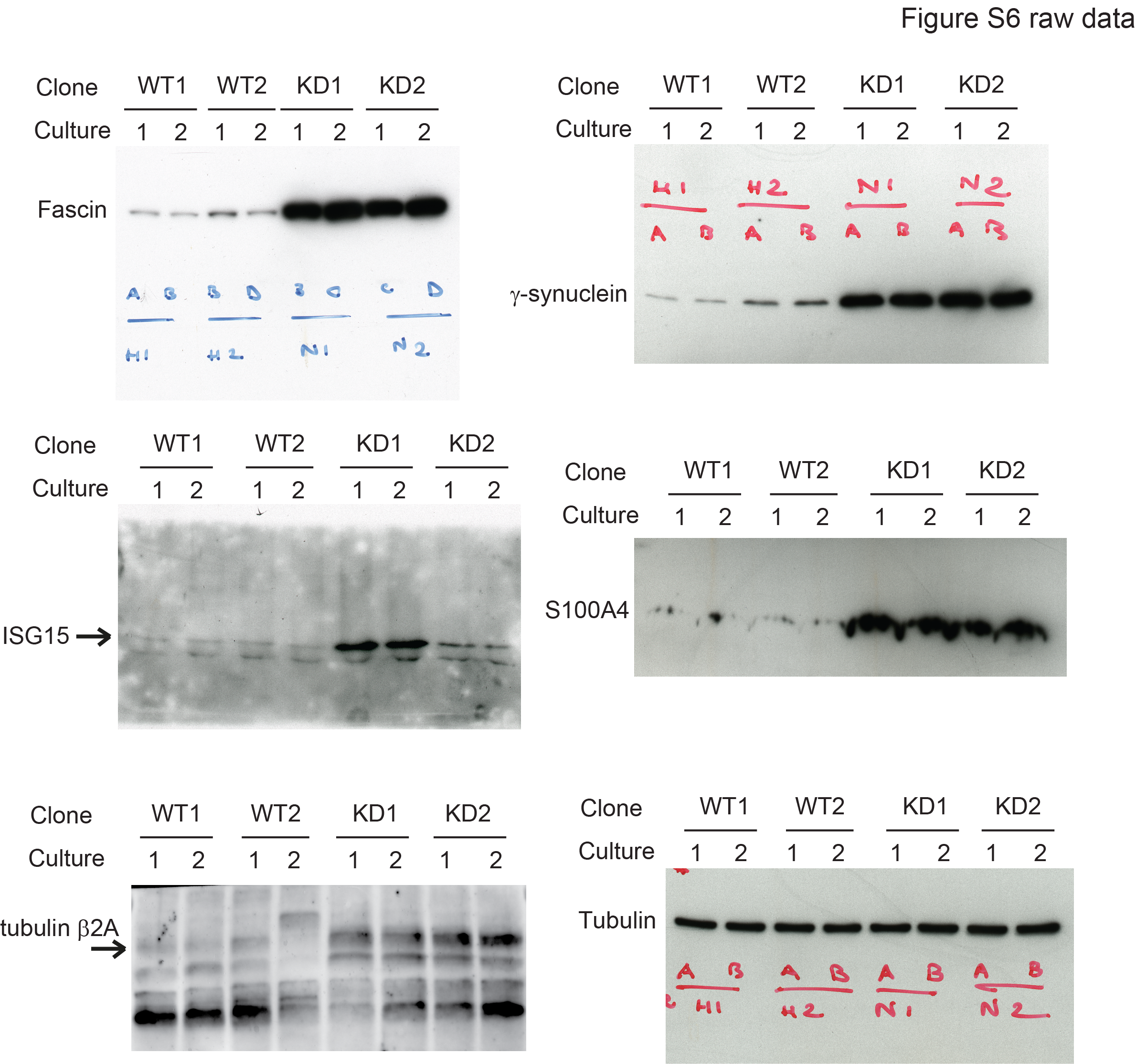

Supplement: Supplementary file 26 — Additional file 26. Images of the full immunoblots. [file 12915_2021_1155_MOESM26_ESM.zip › 26/Additional file 12, Fig. S6.tif]

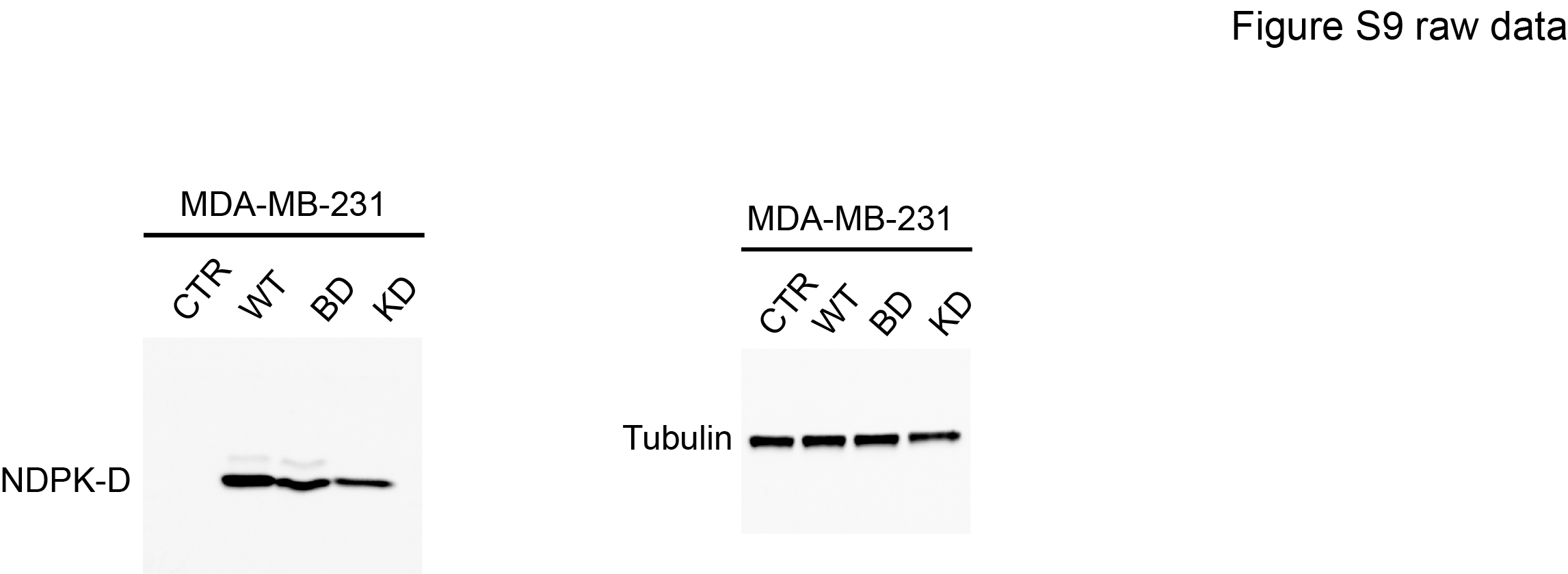

Supplement: Supplementary file 26 — Additional file 26. Images of the full immunoblots. [file 12915_2021_1155_MOESM26_ESM.zip › 26/Additional file 15, Fig. S9.tif]

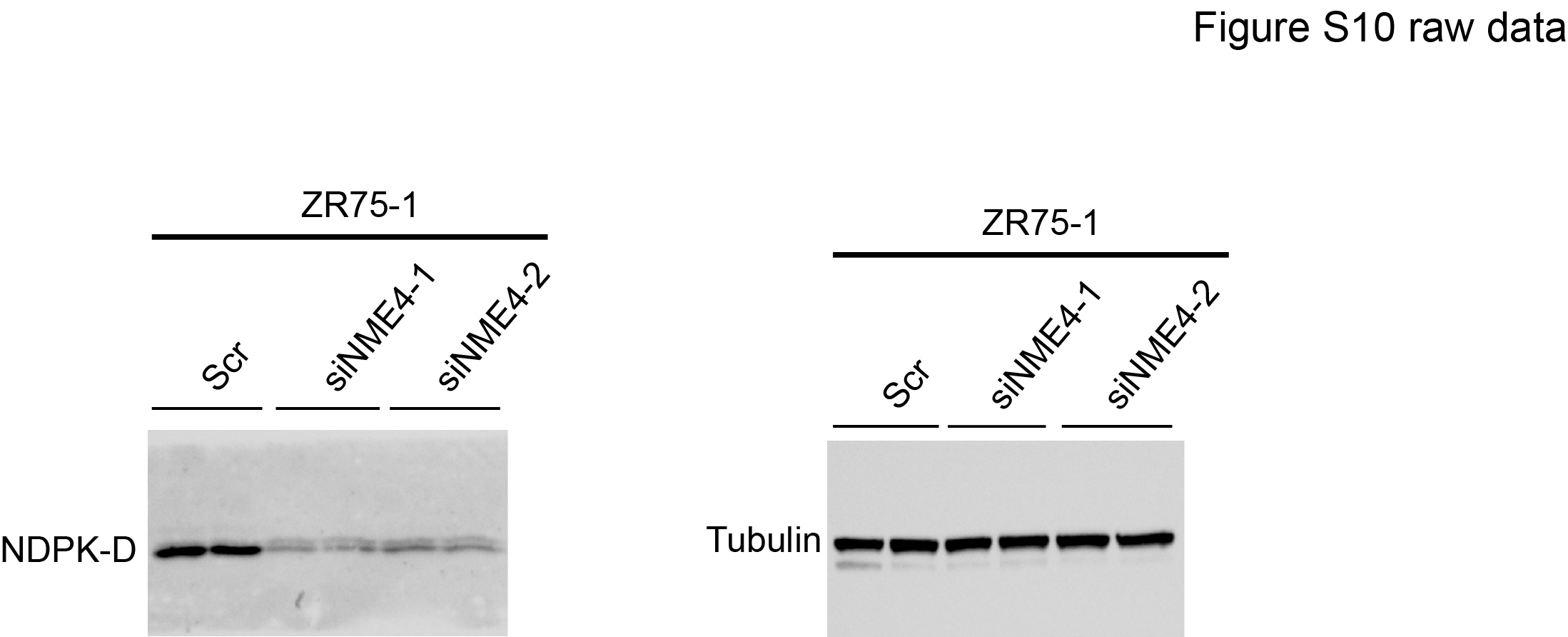

Supplement: Supplementary file 26 — Additional file 26. Images of the full immunoblots. [file 12915_2021_1155_MOESM26_ESM.zip › 26/Additional file 16, Fig. S10.tif]

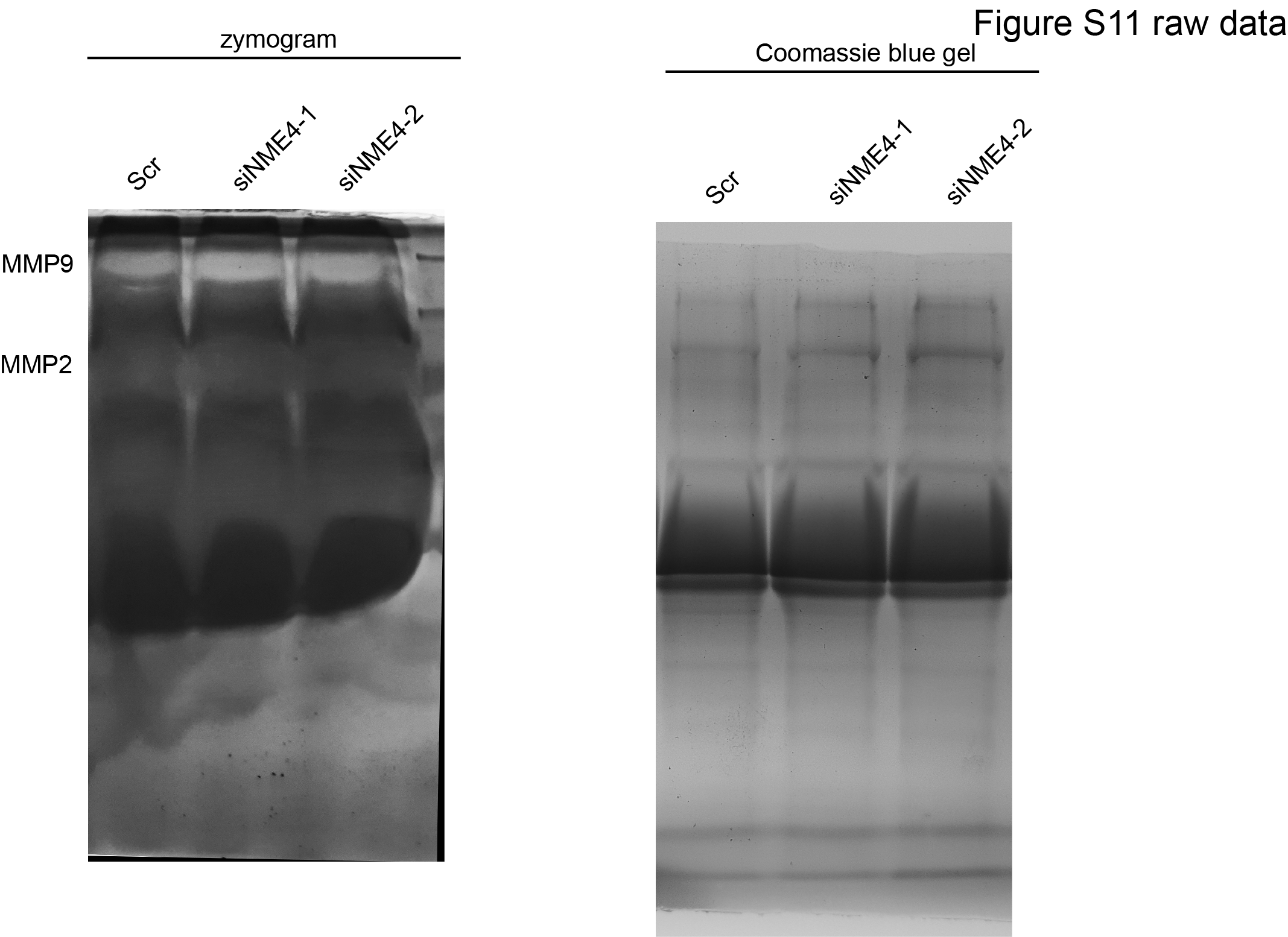

Supplement: Supplementary file 26 — Additional file 26. Images of the full immunoblots. [file 12915_2021_1155_MOESM26_ESM.zip › 26/Additional file 17, Fig. S11.tif]

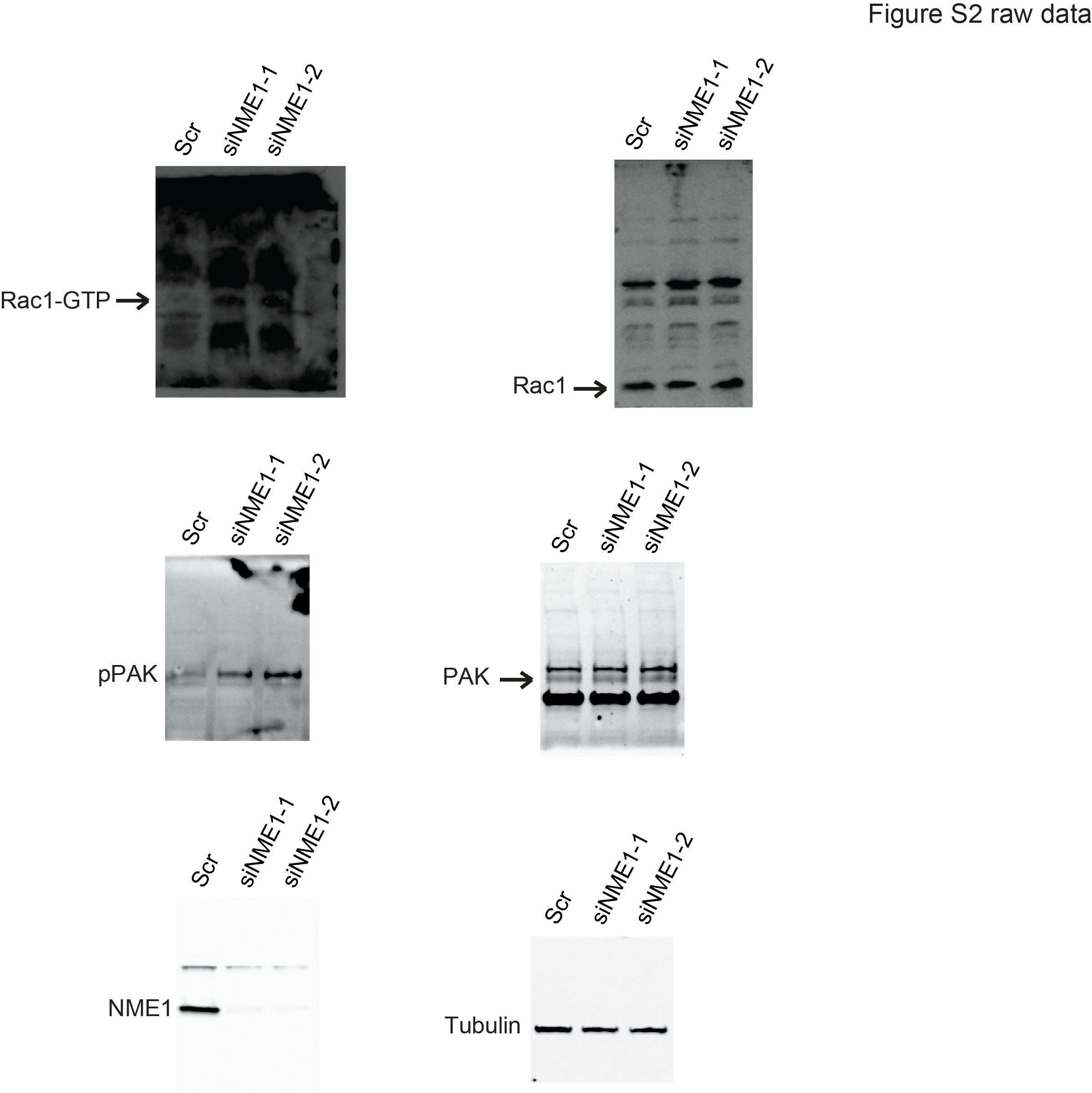

Supplement: Supplementary file 26 — Additional file 26. Images of the full immunoblots. [file 12915_2021_1155_MOESM26_ESM.zip › 26/Additional file 6, Fig. S2.png]

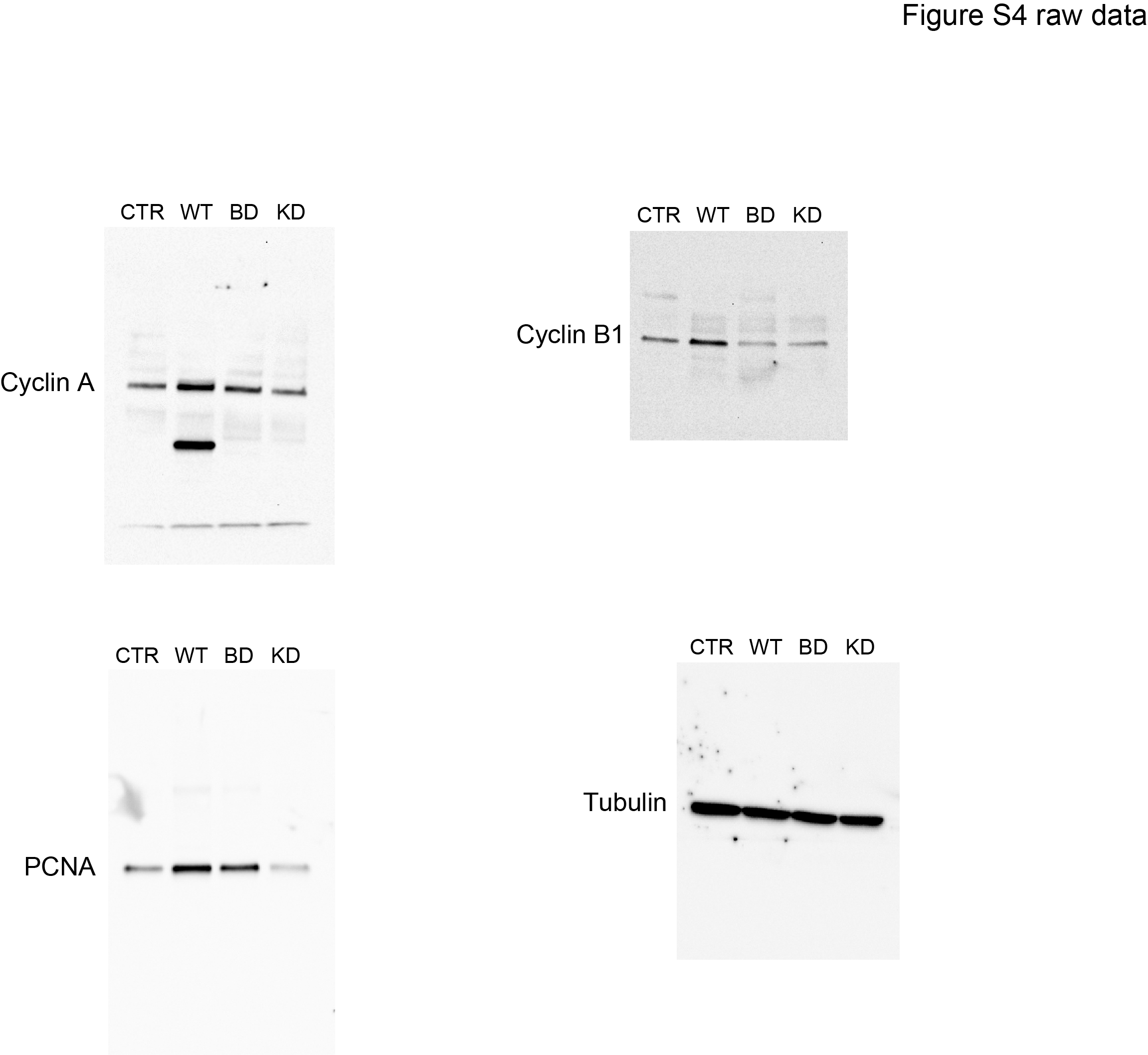

Supplement: Supplementary file 26 — Additional file 26. Images of the full immunoblots. [file 12915_2021_1155_MOESM26_ESM.zip › 26/Additional file 8, Fig. S4.tif]

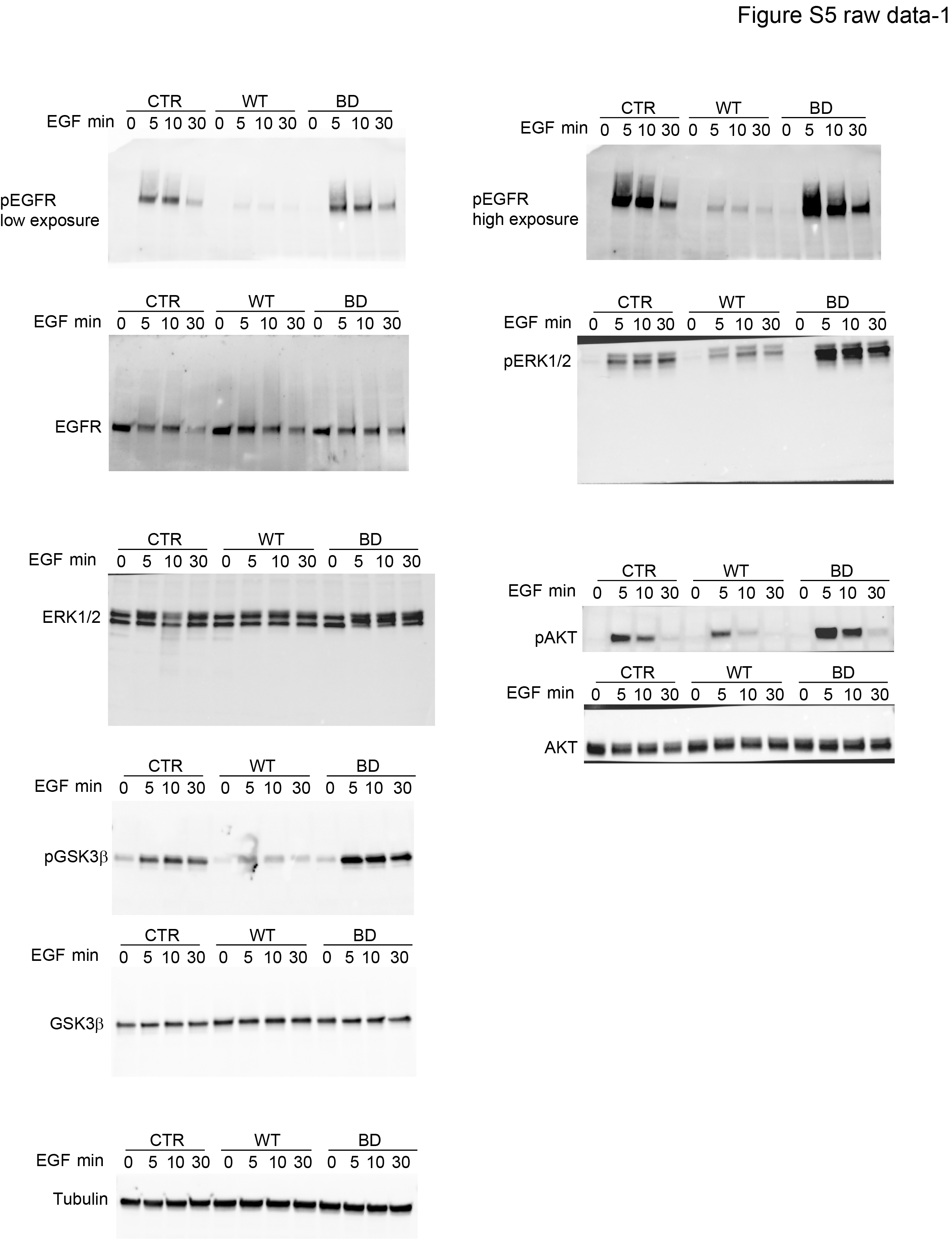

Supplement: Supplementary file 26 — Additional file 26. Images of the full immunoblots. [file 12915_2021_1155_MOESM26_ESM.zip › 26/Additional file 9, Fig. S5 first part.tif]

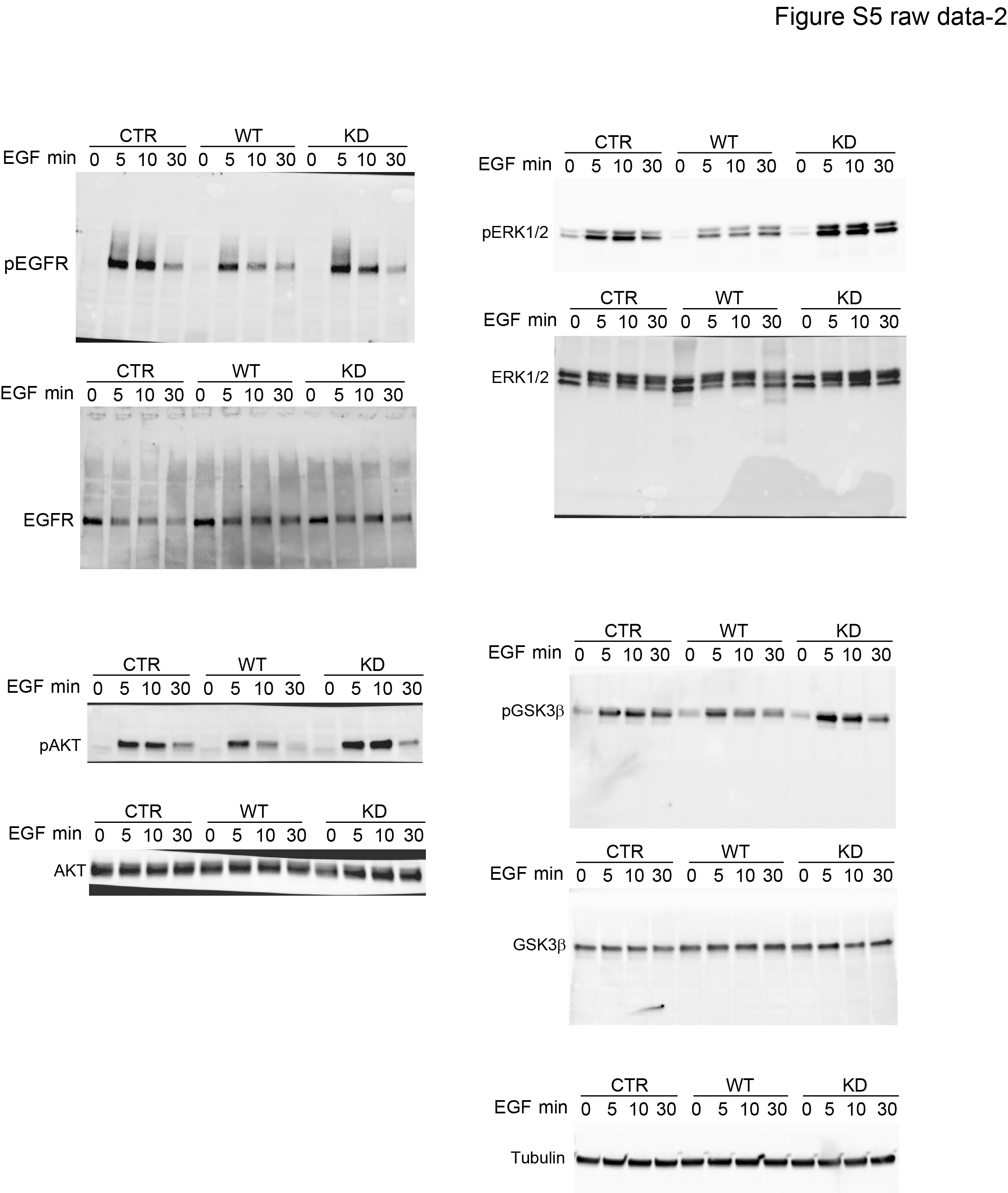

Supplement: Supplementary file 26 — Additional file 26. Images of the full immunoblots. [file 12915_2021_1155_MOESM26_ESM.zip › 26/Additional file 9, Fig. S5 second part.tif]

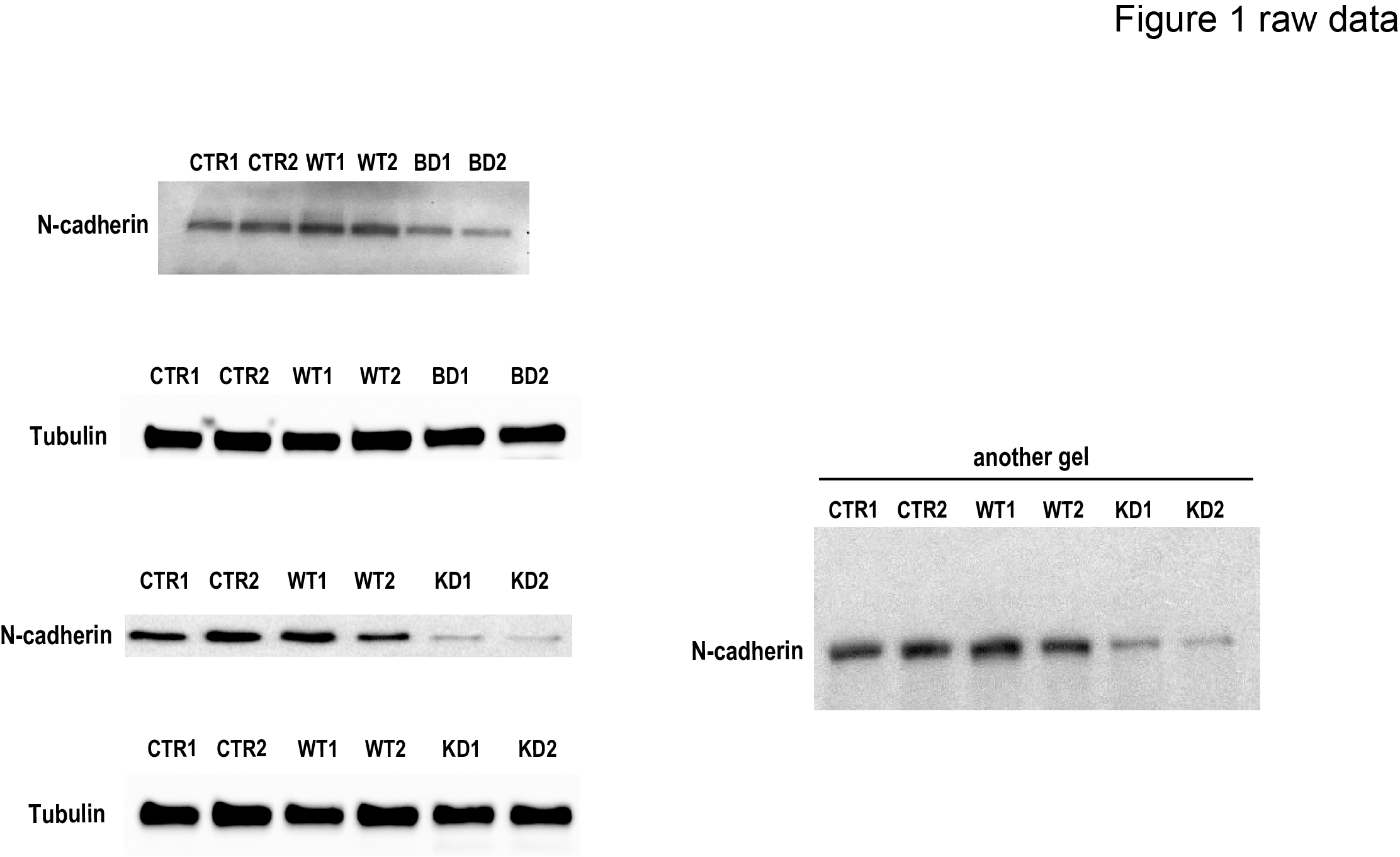

Supplement: Supplementary file 26 — Additional file 26. Images of the full immunoblots. [file 12915_2021_1155_MOESM26_ESM.zip › 26/Figure 1.tif]

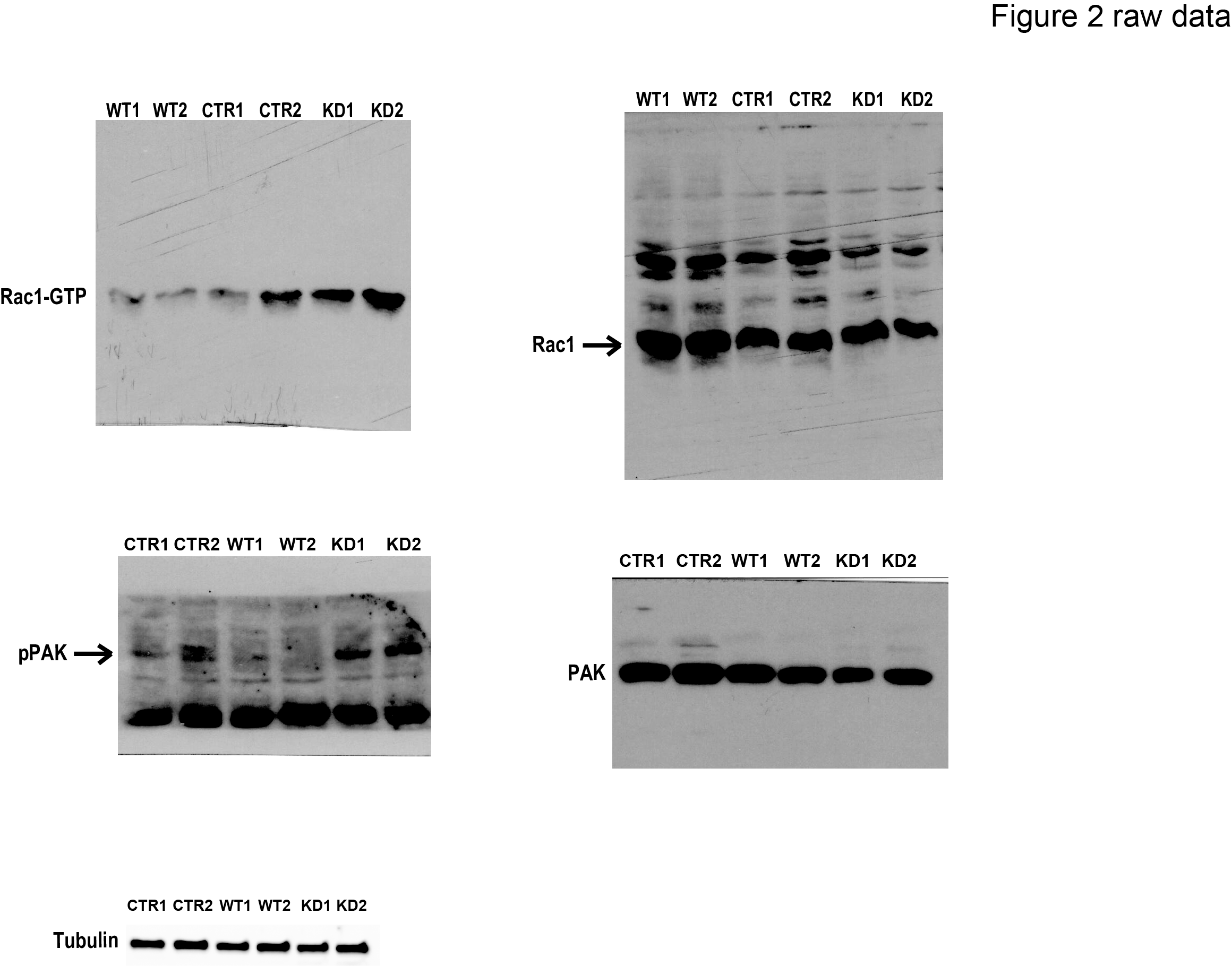

Supplement: Supplementary file 26 — Additional file 26. Images of the full immunoblots. [file 12915_2021_1155_MOESM26_ESM.zip › 26/Figure 2.tif]

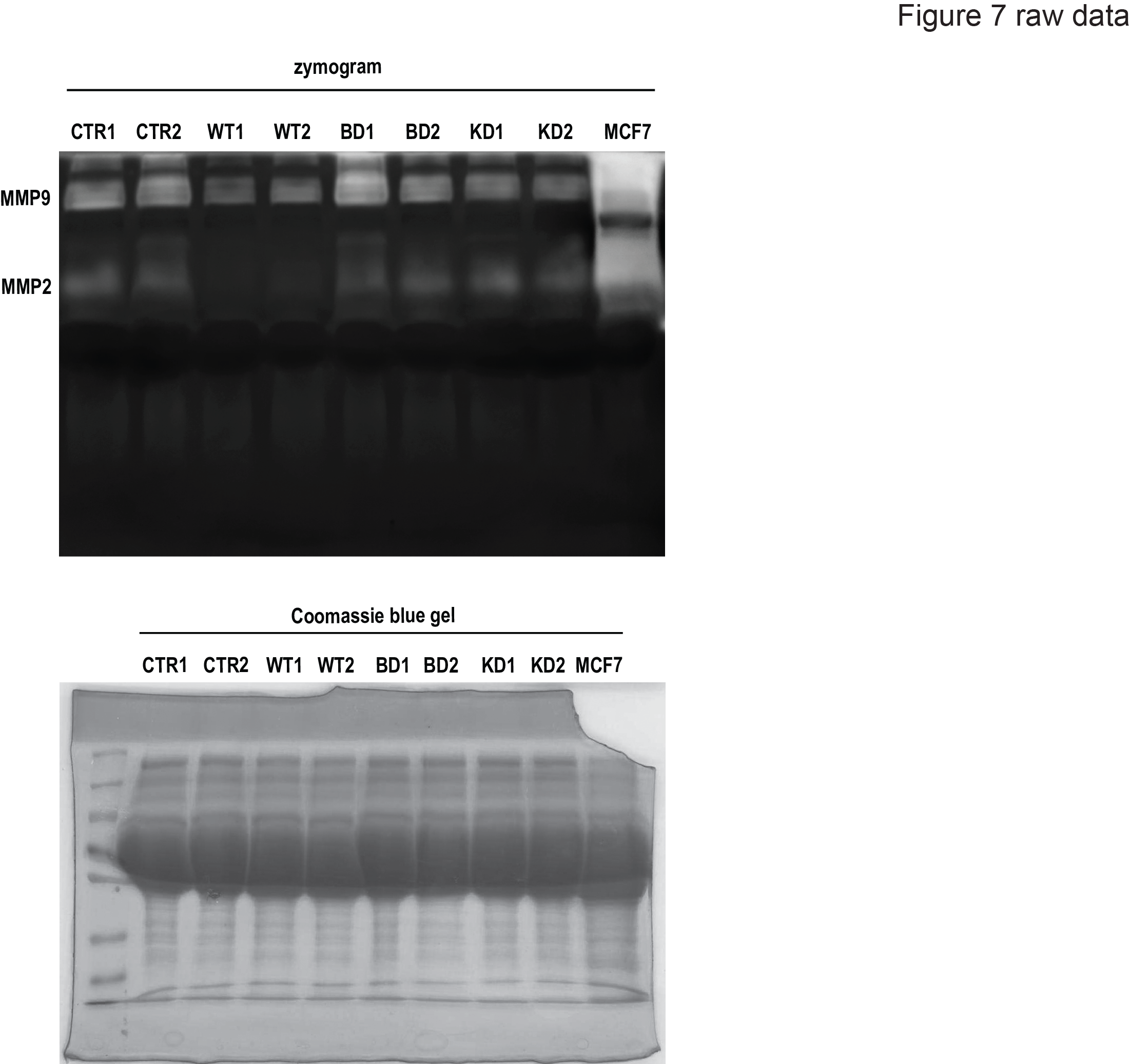

Supplement: Supplementary file 26 — Additional file 26. Images of the full immunoblots. [file 12915_2021_1155_MOESM26_ESM.zip › 26/Figure 7.png]
