# Supplementary material for: The mitochondrially-localized nucleoside diphosphate kinase D (NME4) is a novel metastasis suppressor
Source: BMC Biol. 2021 Oct 21;19:228. doi: 10.1186/s12915-021-01155-5 (PMC8529772; doi:10.1186/s12915-021-01155-5)
Supplement: Supplementary file 10 — Additional file 10: Table S1. Differently expressed proteins in HeLa clones expressing the mutant and the wild type NDPK-D. The full protein names are from the UniProt database. Accession number are from UniProt (Acc._HUMAN) and SwissProt databases. The one-way analysis of variance (ANOVA) test, followed by a Tukey’s multiple comparison test, was used to determine protein spots significantly different between analyses. p-values were calculated across pairwise comparisons (clones KD vs WT, BD vs WT and CTR vs WT) and considered significant when < 0.05. Proteins were ordered following the fold changes in the KD vs WT comparison. * Two identifications for the same spot. Bold values, fold change statistically significant (p< 0.05) and ≥1.3. Italic values, fold change not statistically valid (p > 0.05) or ≤1.3. § Proteins reported to present a mitochondrial localization (UniProt annotation) are indicated by M. [file 12915_2021_1155_MOESM10_ESM.docx]

Table S1: Differently expressed proteins in HeLa clones expressing the wild type and the mutant NDPK-D

| **Spot n°** | **Protein name** | **Accession** | |  |  | **N#H** | | **R#H** | | **V#H** | |
| --- | --- | --- | --- | --- | --- | --- | --- | --- | --- | --- | --- |
|  |  | **Uniprot** | **Swiss Prot** | **§** | **1-ANOVA** | **P-value** | **FC** | **P-value** | **FC** | **P-value** | **FC** |
| **2428** | **Ubiquitin-like protein ISG15** | **ISG15** | **P05161** |  | **2,9E-10** | **3,6E-06** | **8,1** | **5,9E-04** | **4,4** | **1,9E-02** | **5,3** |
| **2419** | **Gamma-synuclein** | **SYUG** | **O76070** |  | **0,0E+00** | **6,1E-13** | **7,8** | **7,2E-11** | **10,8** | **1,1E-04** | **3,9** |
| **1494** | **Aldo-keto reductase family 1 member C1** | **AK1C1** | **Q04828** |  | **7,9E-12** | **2,7E-07** | **5,0** | **1,2E-02** | **2,8** | *5,3E-02* | *2,9* |
| **2607** | **Protein S100-A4** | **S10A4** | **P26447** |  | **2,5E-13** | **2,1E-09** | **4,9** | **7,3E-09** | **4,9** | **1,0E-02** | **1,5** |
| **2599** | **Protein S100-A4** | **S10A4** | **P26447** |  | **4,8E-13** | **1,1E-08** | **4,6** | **1,8E-10** | **5,2** | **1,0E-03** | **1,5** |
| **1167** | **Tubulin beta-2A chain** | **TBB2A** | **Q13885** |  | **0,0E+00** | **1,3E-05** | **3,9** | **1,3E-05** | **3,8** | *4,5E-01* | *1,5* |
| **1409** | **Serpin B5** | **SPB5** | **P36952** |  | **2,1E-11** | **2,2E-12** | **3,5** | **1,4E-08** | **3,7** | *9,2E-01* | *1,3* |
| **1411** | **Serpin B5** | **SPB5** | **P36952** |  | **7,9E-13** | **2,5E-10** | **3,2** | **2,4E-08** | **3,4** | *9,3E-01* | *1,2* |
| **1654** | **Annexin A3** | **ANXA3** | **P12429** |  | **1,2E-10** | **8,0E-07** | **3,0** | **7,9E-04** | **2,5** | **1,2E-02** | **1,5** |
| **1497** | **Aldo-keto reductase family 1 member C1** | **AK1C1** | **Q04828** |  | **1,3E-13** | **6,8E-07** | **2,9** | *1,2E-01* | *1,7* | **5,7E-03** | **2,7** |
| **2610** | **Protein S100-A4** | **S10A4** | **P26447** |  | **1,2E-07** | **1,1E-05** | **2,8** | **1,4E-05** | **2,8** | *2,6E-01* | *1,1* |
| **2653** | **Protein S100-A6** | **S10A6** | **P06703** |  | **5,8E-06** | **4,7E-04** | **2,7** | **2,3E-03** | **6,5** | *2,8E-01* | *1,3* |
| **2571** | **Protein S100-A16** | **S10AG** | **Q96FQ6** |  | **5,6E-12** | **1,9E-07** | **2,7** | **1,1E-06** | **2,6** | **2,0E-05** | **-2,3** |
| **1070** | **Fascin** | **FSCN1** | **Q16658** |  | **2,7E-09** | **2,6E-07** | **2,6** | **2,4E-07** | **2,8** | **7,6E-03** | **1,4** |
| **2485** | **Fatty acid-binding protein, heart** | **FABPH** | **P05413** |  | **5,0E-15** | **5,0E-08** | **2,6** | **4,1E-04** | **3,5** | **3,5E-04** | **2,3** |
| **1168** | **Tubulin beta-2A chain** | **TBB2A** | **Q13885** |  | **6,0E-14** | **1,6E-04** | **2,6** | **5,7E-05** | **2,5** | *5,6E-01* | *1,2* |
| **750** | **X-ray repair cross-complementing protein 6** | **XRCC6** | **P12956** |  | **7,3E-07** | **2,1E-04** | **2,5** | *3,3E-01* | *1,1* | *2,3E-01* | *1,2* |
| **1361** | **Macrophage-capping protein** | **CAPG** | **P40121** |  | **2,7E-15** | **3,1E-09** | **2,5** | **1,4E-09** | **2,4** | *8,6E-01* | *1,0* |
| **1769** | **Voltage-dependent anion-selective channel protein** | **VDAC3** | **Q9Y277** | **M** | **1,6E-06** | **2,1E-03** | **2,4** | *2,5E-01* | *1,3* | **1,1E-03** | **4,5** |
| **1069** | **Fascin** | **FSCN1** | **Q16658** |  | **2,3E-08** | **2,4E-06** | **2,3** | **1,3E-06** | **2,3** | **4,4E-03** | **1,5** |
| **1166** | **Tubulin beta-2A chain** | **TBB2A** | **Q13885** |  | **2,0E-12** | **1,2E-08** | **2,2** | **3,1E-06** | **2,2** | *2,3E-01* | *-1,2* |
| **2547*** | **Cystatin-B** | **CYTB** | **P04080** |  | **1,3E-11** | **2,2E-08** | **2,2** | **3,5E-07** | **1,5** | *2,5E-04* | *1,2* |
| **2547*** | **Putative peptidyl-tRNA hydrolase PTRHD1** | **PTRD1** | **Q6GMV3** |  | **1,3E-11** | **2,2E-08** | **2,2** | **3,5E-07** | **1,5** | *2,5E-04* | *1,2* |
| **749** | **X-ray repair cross-complementing protein 6** | **XRCC6** | **P12956** |  | **1,3E-06** | **8,1E-05** | **2,2** | *9,2E-01* | *-1,0* | *3,6E-01* | *1,1* |
| **924** | **26S protease regulatory subunit 7** | **PRS7** | **P35998** |  | **3,2E-06** | **7,8E-05** | **2,2** | **2,0E-03** | **1,8** | *4,7E-01* | *1,1* |
| **2651** | **Protein S100-A6** | **S10A6** | **P06703** |  | **2,4E-08** | **1,6E-05** | **2,2** | **1,6E-06** | **2,0** | **3,1E-02** | **1,4** |
| **2645** | **Protein S100-A6** | **S10A6** | **P06703** |  | **7,7E-10** | **2,3E-06** | **2,1** | **2,2E-07** | **2,1** | **1,3E-02** | **1,4** |
| **1960** | **Glutathione S-transferase Mu 4** | **GSTM4** | **Q03013** |  | **1,4E-09** | **2,6E-07** | **2,1** | **2,0E-03** | **1,4** | **8,6E-05** | **1,3** |
| **2648** | **Protein S100-A6** | **S10A6** | **P06703** |  | **3,8E-06** | **2,3E-05** | **2,1** | **3,2E-05** | **2,0** | **4,0E-02** | **1,5** |
| **2566** | **Protein S100-A13** | **S10AD** | **Q99584** |  | **4,9E-11** | **3,0E-05** | **2,1** | **2,4E-04** | **1,8** | *1,2E-01* | *-1,2* |
| **1808** | **Uridine-cytidine kinase 2** | **UCK2** | **Q9BZX2** |  | **2,5E-08** | **5,5E-06** | **2,0** | **1,4E-05** | **1,9** | **2,8E-05** | **1,9** |
| **1526** | **Aldo-keto reductase family 1 member C1** | **AK1C1** | **Q04828** |  | **7,2E-07** | **4,0E-05** | **2,0** | **4,7E-03** | **1,5** | **7,9E-03** | **1,9** |
| **1532** | **Aldo-keto reductase family 1 member C3** | **AK1C3** | **P42330** |  | **1,0E-04** | **1,6E-04** | **2,0** | *6,0E-02* | *1,4* | **2,4E-02** | **1,6** |
| **2192** | **Protein DEK** | **DEK** | **P35659** |  | **3,6E-07** | **2,8E-04** | **2,0** | **2,1E-05** | **2,4** | **4,0E-04** | **1,8** |
| **2647** | **Protein S100-A6** | **S10A6** | **P06703** |  | **2,6E-07** | **7,8E-04** | **1,9** | **1,2E-02** | **2,9** | *1,9E-01* | *1,3* |
| **748** | **X-ray repair cross-complementing protein 6** | **XRCC6** | **P12956** |  | **9,4E-06** | **4,8E-05** | **1,9** | *1,1E-01* | *1,1* | *2,3E-01* | *1,1* |
| **1659*** | **HCLS1-associated protein X-1** | **HAX1** | **O00165** | **M** | **9,0E-07** | **2,5E-07** | **1,9** | **6,2E-07** | **1,7** | *1,6E-01* | *1,2* |
| **1659*** | **Ubiquitin thioesterase OTUB1** | **OTUB1** | **Q96FW1** |  | **9,0E-07** | **2,5E-07** | **1,9** | **6,2E-07** | **1,7** | *1,6E-01* | *1,2* |
| **1938** | **Glutathione S-transferase Mu 3** | **GSTM3** | **P21266** |  | **7,4E-13** | **1,1E-09** | **1,9** | **1,8E-05** | **1,6** | **1,6E-04** | **1,3** |
| **2591** | **Protein S100-A11** | **S10AB** | **P31949** |  | **3,9E-09** | **4,5E-04** | **1,8** | **1,0E-05** | **2,6** | *5,1E-01* | *1,1* |
| **2480** | **Profilin-2** | **PROF2** | **P35080** |  | **3,8E-10** | **6,5E-08** | **1,8** | **3,0E-05** | **1,5** | *2,1E-01* | *1,1* |
| **2300** | **Stathmin** | **STMN1** | **P16949** |  | **4,7E-12** | **2,6E-08** | **1,8** | **3,2E-07** | **1,6** | *4,5E-01* | *-1,1* |
| **2292** | **Stathmin** | **STMN1** | **P16949** |  | **1,8E-12** | **1,0E-10** | **1,8** | **1,8E-08** | **1,6** | **2,8E-04** | **1,3** |
| **2240** | **Superoxide dismutase** | **SODC** | **P00441** |  | **8,9E-10** | **1,1E-08** | **1,8** | **4,7E-06** | **1,3** | **7,9E-05** | **1,3** |
| **1076** | **Keratin, type II cytoskeletal 7** | **K2C7** | **P08729** |  | **5,4E-11** | **6,5E-08** | **1,7** | **2,8E-03** | **1,5** | *3,3E-01* | *1,2* |
| **1080** | **Keratin, type II cytoskeletal 7** | **K2C7** | **P08729** |  | **5,1E-10** | **9,9E-08** | **1,7** | *1,6E-01* | *1,4* | **1,4E-02** | **1,5** |
| **2285** | **Stathmin** | **STMN1** | **P16949** |  | **1,1E-08** | **6,2E-07** | **1,7** | **3,7E-05** | **1,3** | *7,0E-02* | *-1,2* |
| **1103** | **Septin-11** | **SEP11** | **Q9NVA2** |  | **6,4E-10** | **8,5E-10** | **1,7** | **6,7E-04** | **1,3** | **7,2E-06** | **1,3** |
| **2216** | **NADH dehydrogenase 1 alpha subcomplex subunit 8** | **NDUA8** | **P51970** | **M** | **7,1E-09** | **9,9E-10** | **1,7** | **3,6E-07** | **1,7** | *9,9E-02* | *1,1* |
| **2596** | **Protein S100-A11** | **S10AB** | **P31949** |  | **9,3E-10** | **1,1E-03** | **1,7** | **1,2E-05** | **2,2** | *2,2E-01* | *1,2* |
| **1932** | **Glutathione S-transferase Mu 3** | **GSTM3** | **P21266** |  | **4,5E-10** | **6,1E-09** | **1,7** | **5,3E-08** | **1,6** | **1,5E-03** | **1,3** |
| **789** | **Succinate dehydrogenase** | **SDHA** | **P31040** | **M** | **1,5E-08** | **7,2E-08** | **1,7** | **5,6E-06** | **1,6** | **1,2E-05** | **2,0** |
| **1861** | **Calcyclin-binding protein** | **CYBP** | **Q9HB71** |  | **1,6E-12** | **8,5E-07** | **1,7** | **4,4E-07** | **1,7** | **2,3E-04** | **1,6** |
| **2388** | **Histone H2B type 1-C/E/F/G/I** | **H2B1C** | **P62807** |  | **1,1E-04** | **1,4E-04** | **1,7** | **7,8E-05** | **1,9** | *1,3E-01* | *1,2* |
| **2518*** | **Peptidyl-prolyl cis-trans isomerase FKBP1A** | **FKB1A** | **P62942** |  | **5,0E-09** | **1,1E-05** | **1,7** | **1,8E-04** | **1,5** | *1,2E-02* | *1,2* |
| **2518*** | **Histone H4** | **H4** | **P62805** |  | **5,0E-09** | **1,1E-05** | **1,7** | **1,8E-04** | **1,5** | *1,2E-02* | *1,2* |
| **1545** | **Aldose reductase** | **ALDR** | **P15121** |  | **4,9E-06** | **6,2E-05** | **1,6** | *7,4E-02* | *1,3* | *1,4E-01* | *-1,1* |
| **1933** | **Glutathione S-transferase Mu 3** | **GSTM3** | **P21266** |  | **9,6E-10** | **1,2E-07** | **1,6** | **2,2E-06** | **1,4** | *1,9E-02* | *1,2* |
| **2351** | **Allograft inflammatory factor 1-like** | **AIF1L** | **Q9BQI0** |  | **1,0E-04** | **7,8E-05** | **1,6** | **1,2E-05** | **1,9** | *8,5E-01* | *-1,0* |
| **2459** | **Protein S100-A8** | **S10A8** | **P05109** |  | **2,9E-09** | **5,6E-03** | **1,6** | *5,1E-01* | *1,1* | **3,6E-02** | **1,3** |
| **2299** | **Stathmin** | **STMN1** | **P16949** |  | **5,0E-12** | **4,6E-06** | **1,6** | **8,5E-07** | **1,5** | *6,7E-01* | *1,1* |
| **392** | **Programmed cell death 6-interacting protein** | **PDC6I** | **Q8WUM4** |  | **4,1E-10** | **1,3E-06** | **1,6** | **5,1E-07** | **1,7** | *2,0E-02* | *1,2* |
| **1906** | **Calpain small subunit 1** | **CPNS1** | **P04632** |  | **2,3E-07** | **7,3E-06** | **1,6** | **1,7E-05** | **1,5** | *5,1E-01* | *1,1* |
| **1374** | **Macrophage-capping protein** | **CAPG** | **P40121** |  | **2,9E-10** | **1,4E-07** | **1,6** | **7,1E-07** | **1,6** | *2,2E-01* | *1,1* |
| **718** | **X-ray repair cross-complementing protein 6** | **XRCC6** | **P12956** |  | **1,2E-03** | **5,5E-05** | **1,6** | *1,3E-02* | *1,2* | *1,0E-01* | *1,2* |
| **1484** | **Annexin A1** | **ANXA1** | **P04083** |  | **3,3E-10** | **8,3E-05** | **1,6** | **6,3E-05** | **1,8** | **3,0E-02** | **1,3** |
| **2036** | **Flavin reductase (NADPH)** | **BLVRB** | **P30043** |  | **1,2E-08** | **1,0E-06** | **1,6** | *1,5E-01* | *1,1* | *1,8E-01* | *-1,2* |
| **2083** | **Peroxiredoxin-1** | **PRDX1** | **Q06830** |  | **1,1E-14** | **5,3E-05** | **1,6** | **1,7E-06** | **1,6** | **5,5E-05** | **2,0** |
| **1013** | **Adenylyl cyclase-associated protein 1** | **CAP1** | **Q01518** |  | **7,5E-07** | **7,7E-04** | **1,6** | **6,1E-07** | **1,8** | **9,5E-04** | **1,7** |
| **1018** | **Adenylyl cyclase-associated protein 1** | **CAP1** | **Q01518** |  | **1,7E-15** | **3,0E-05** | **1,5** | **1,9E-08** | **1,7** | **1,5E-03** | **1,4** |
| **2106** | **Interleukin-18** | **IL18** | **Q14116** |  | **8,4E-11** | **1,0E-06** | **1,5** | **4,9E-02** | **1,3** | **9,1E-04** | **1,6** |
| **2449** | **Fatty acid-binding protein, epidermal** | **FABP5** | **Q01469** |  | **1,6E-10** | **1,2E-06** | **1,5** | **9,9E-08** | **1,5** | **2,0E-03** | **-1,3** |
| **2462** | **Profilin-1** | **PROF1** | **P07737** |  | **6,5E-07** | **1,5E-06** | **1,5** | **7,6E-05** | **1,5** | *6,6E-02* | *1,1* |
| **1950** | **Triosephosphate isomerase** | **TPIS** | **P60174** |  | **6,5E-08** | **3,8E-04** | **1,5** | *5,1E-01* | *-1,1* | *6,4E-01* | *-1,0* |
| **2475** | **Profilin-2** | **PROF2** | **P35080** |  | **3,3E-10** | **2,2E-08** | **1,5** | **2,3E-07** | **1,5** | *1,7E-02* | *-1,2* |
| **387** | **Programmed cell death 6-interacting protein** | **PDC6I** | **Q8WUM4** |  | **7,3E-10** | **1,2E-05** | **1,5** | **2,2E-06** | **1,6** | *1,2E-01* | *1,1* |
| **1064** | **Keratin, type II cytoskeletal 8** | **K2C8** | **P05787** |  | **3,8E-10** | **3,3E-04** | **1,5** | **2,5E-03** | **1,4** | *4,7E-01* | *-1,1* |
| **1397** | **Serpin B6** | **SPB6** | **P35237** |  | **1,0E-10** | **1,0E-10** | **1,5** | **4,3E-06** | **1,6** | *9,2E-01* | *1,0* |
| **1527** | **Annexin A1** | **ANXA1** | **P04083** |  | **2,9E-10** | **2,1E-04** | **1,5** | **5,0E-05** | **1,7** | **4,8E-02** | **1,3** |
| **1857** | **Calcyclin-binding protein** | **CYBP** | **Q9HB71** |  | **6,7E-11** | **1,5E-06** | **1,5** | **1,9E-07** | **1,6** | **5,3E-06** | **1,4** |
| **2120** | **Transgelin-2** | **TAGL2** | **P37802** |  | **9,2E-11** | **9,2E-06** | **1,5** | **1,0E-06** | **1,8** | *1,9E-02* | *1,2* |
| **2322** | **Prefoldin subunit 2** | **PFD2** | **Q9UHV9** |  | **1,5E-09** | **1,9E-08** | **1,5** | **2,1E-05** | **1,6** | **1,7E-05** | **1,4** |
| **1723** | **Tetratricopeptide repeat protein 33** | **TTC33** | **Q6PID6** |  | **2,7E-02** | **5,8E-03** | **1,5** | **2,9E-03** | **2,0** | **4,6E-02** | **1,5** |
| **668** | **Prelamin-A/C** | **LMNA** | **P02545** |  | **1,4E-12** | **2,3E-07** | **1,5** | **1,2E-07** | **1,4** | **7,5E-06** | **1,3** |
| **2266** | **Cofilin-1** | **COF1** | **P23528** |  | **1,4E-08** | **2,3E-05** | **1,5** | **8,7E-07** | **1,5** | *6,9E-01* | *-1,0* |
| **2080** | **Peroxiredoxin-1** | **PRDX1** | **Q06830** |  | **2,6E-11** | **3,1E-05** | **1,5** | **2,7E-08** | **1,6** | **1,7E-05** | **1,8** |
| **2208** | **Deoxyuridine 5'-triphosphate nucleotidohydrol.** | **DUT** | **P33316** | **M** | **7,6E-11** | **5,3E-05** | **1,5** | **2,1E-03** | **1,5** | *9,6E-01* | *1,0* |
| **1330** | **Leukocyte elastase inhibitor** | **ILEU** | **P30740** |  | **1,2E-09** | **1,2E-05** | **1,5** | **1,2E-05** | **1,5** | *5,3E-01* | *1,1* |
| **1837** | **Chloride intracellular channel protein 4** | **CLIC4** | **Q9Y696** |  | **8,0E-05** | **5,1E-04** | **1,5** | **1,8E-03** | **1,6** | *7,9E-01* | *-1,0* |
| **1751** | **Tropomyosin alpha-4 chain** | **TPM4** | **P67936** |  | **4,6E-07** | **3,2E-07** | **1,4** | **5,9E-07** | **1,5** | **3,2E-05** | **1,3** |
| **543** | **Heat shock 70 kDa protein 1A/1B** | **HSP71** | **P08107** |  | **3,2E-03** | **3,4E-02** | **1,4** | **8,2E-04** | **1,8** | *5,2E-02* | *1,4* |
| **1523** | **Annexin A1** | **ANXA1** | **P04083** |  | **4,7E-10** | **1,3E-05** | **1,4** | **7,6E-06** | **1,5** | *3,2E-02* | *1,2* |
| **1939*** | **UPF0568 protein C14orf166** | **CN166** | **Q9Y224** |  | **3,6E-10** | **1,1E-05** | **1,4** | **5,3E-09** | **1,5** | *1,9E-03* | *1,2* |
| **1939*** | **Peroxiredoxin-6** | **PRDX6** | **P30041** |  | **3,6E-10** | **1,1E-05** | **1,4** | **5,3E-09** | **1,5** | *1,9E-03* | *1,2* |
| **1265** | **Protein SET** | **SET** | **Q01105** |  | **6,2E-04** | **4,8E-03** | **1,4** | **5,0E-03** | **1,6** | *7,7E-02* | *1,2* |
| **1254** | **Protein SET** | **SET** | **Q01105** |  | **1,2E-08** | **8,4E-07** | **1,4** | **1,5E-08** | **1,5** | *1,5E-05* | *1,2* |
| **2001** | **Lactoylglutathione lyase** | **LGUL** | **Q04760** |  | **1,4E-09** | **2,0E-04** | **1,4** | **5,4E-08** | **1,7** | *4,3E-02* | *1,1* |
| **1677** | **Phosphatidylinositol transfer protein alpha isoform** | **PIPNA** | **Q00169** |  | **7,0E-06** | **5,6E-04** | **1,4** | **3,9E-05** | **1,5** | *8,9E-01* | *-1,0* |
| **592** | **Ezrin** | **EZRI** | **P15311** |  | **3,6E-13** | **1,4E-07** | **1,3** | **4,7E-08** | **1,5** | *2,5E-01* | *1,0* |
| **2154** | **Diablo homolog** | **DBLOH** | **Q9NR28** | **M** | **3,8E-06** | **3,1E-04** | **1,3** | **8,2E-07** | **1,6** | **4,8E-05** | **1,3** |
| **2481** | **14 kDa phosphohistidine phosphatase** | **PHP14** | **Q9NRX4** |  | **4,3E-08** | **1,2E-03** | **1,3** | **2,5E-06** | **1,7** | *7,4E-01* | *-1,0* |
| **2186** | **Prostaglandin E synthase 3** | **TEBP** | **Q15185** |  | **8,7E-14** | **3,6E-05** | **1,3** | **1,5E-09** | **2,5** | *3,4E-03* | *1,2* |
| **2219*** | **Actin-related protein 2/3 complex subunit 4** | **ARPC4** | **P59998** |  | **8,4E-02** | **2,3E-02** | **1,3** | **2,3E-03** | **1,5** | *2,7E-01* | *1,2* |
| **2219*** | **Eukaryotic translation elongation factor 1 epsilon-1** | **MCA3** | **O43324** |  | **8,4E-02** | **2,3E-02** | **1,3** | **2,3E-03** | **1,5** | *2,7E-01* | *1,2* |
| **2379** | **Protein mago nashi homolog** | **MGN** | **P61326** |  | **2,9E-03** | **6,4E-03** | **1,3** | **3,2E-04** | **1,6** | **1,6E-02** | **1,3** |
| **1642** | **Phosphatidylinositol transfer protein beta isoform** | **PIPNB** | **P48739** |  | **1,3E-04** | **5,4E-03** | **1,3** | **5,1E-05** | **1,7** | *7,0E-01* | *-1,0* |
| **1819** | **S-methyl-5'-thioadenosine phosphorylase** | **MTAP** | **Q13126** |  | **5,7E-08** | **1,9E-03** | **1,3** | **7,7E-04** | **1,5** | *8,9E-01* | *-1,0* |
| **2067** | **Alpha/beta hydrolase domain-containing protein 14B** | **ABHEB** | **Q96IU4** |  | **1,1E-06** | **8,8E-06** | **1,3** | **1,1E-06** | **1,7** | *1,3E-01* | *1,1* |
| **2164** | **Prostaglandin E synthase 3** | **TEBP** | **Q15185** |  | **3,6E-15** | **1,6E-05** | **1,3** | **1,6E-09** | **2,7** | *2,4E-03* | *1,2* |
| **587** | **Ezrin** | **EZRI** | **P15311** |  | **1,3E-08** | **5,6E-05** | **1,3** | **7,0E-07** | **1,4** | *2,5E-01* | *1,0* |
| **947** | **Tyrosine--tRNA ligase, cytoplasmic** | **SYYC** | **P54577** |  | **2,7E-11** | *2,9E-03* | *1,2* | **2,8E-09** | **1,5** | **3,0E-06** | **1,6** |
| **2003** | **Translationally-controlled tumor protein** | **TCTP** | **P13693** |  | **1,8E-08** | *3,2E-04* | *1,2* | **4,2E-05** | **1,7** | *2,6E-03* | *1,2* |
| **2008*** | **Acyl-protein thioesterase 2** | **LYPA2** | **O95372** |  | **7,3E-09** | *3,8E-05* | *1,2* | **1,7E-07** | **1,5** | *9,5E-03* | *1,1* |
| **2008*** | **MOB kinase activator 1A** | **MOB1A** | **Q9H8S9** |  | **7,3E-09** | *3,8E-05* | *1,2* | **1,7E-07** | **1,5** | *9,5E-03* | *1,1* |
| **2540** | **Thioredoxin** | **THIO** | **P10599** |  | **2,4E-11** | *1,2E-02* | *1,2* | **4,7E-08** | **1,6** | **1,5E-03** | **1,4** |
| **1671** | **Glyoxalase domain-containing protein 4** | **GLOD4** | **Q9HC38** |  | **2,1E-07** | *6,1E-02* | *1,2* | **3,6E-06** | **1,6** | *1,1E-01* | *-1,2* |
| **550** | **Heat shock 70 kDa protein 1A/1B** | **HSP71** | **P08107** |  | **4,2E-04** | *1,6E-01* | *1,1* | **1,1E-07** | **1,6** | *9,8E-01* | *1,0* |
| **2584** | **Small nuclear ribonucleoprotein F** | **RUXF** | **P62306** |  | **2,0E-07** | *1,4E-01* | *1,1* | **7,5E-05** | **1,5** | *7,4E-02* | *-1,1* |
| **1661** | **S-formylglutathione hydrolase** | **ESTD** | **P10768** |  | **1,1E-11** | *1,3E-01* | *1,1* | **3,8E-06** | **2,1** | *3,5E-01* | *-1,0* |
| **2277** | **Low Mr phosphotyrosine protein phosphatase** | **PPAC** | **P24666** |  | **3,1E-01** | *5,9E-01* | *1,1* | **1,4E-03** | **1,6** | *3,8E-01* | *1,3* |
| **2057** | **DNA-directed RNA polymerase II subunit RPB7** | **RPB7** | **P62487** |  | **6,4E-07** | *8,7E-01* | *1,0* | **1,1E-05** | **1,7** | *5,9E-02* | *1,3* |
| **1038*** | **Angio-associated migratory cell protein** | **AAMP** | **Q13685** |  | **8,4E-09** | *3,4E-02* | *-1,1* | **8,0E-07** | **-1,4** | *1,5E-01* | *-1,1* |
| **1038*** | **Calreticulin** | **CALR** | **P27797** |  | **8,4E-09** | *3,4E-02* | *-1,1* | **8,0E-07** | **-1,4** | *1,5E-01* | *-1,1* |
| **693** | **Heat shock protein 75 kDa** | **TRAP1** | **Q12931** | **M** | **5,5E-03** | *2,2E-01* | *-1,1* | **2,2E-02** | **-1,5** | *3,2E-01* | *1,2* |
| **419** | **Transitional endoplasmic reticulum ATPase** | **TERA** | **P55072** |  | **8,5E-09** | *2,1E-01* | *-1,1* | **3,2E-03** | **-1,7** | **1,3E-04** | **-1,7** |
| **1220** | **Guanine deaminase** | **GUAD** | **Q9Y2T3** |  | **2,2E-09** | *4,9E-03* | *-1,2* | **9,6E-07** | **-1,5** | *7,9E-02* | *-1,1* |
| **798** | **Calreticulin** | **CALR** | **P27797** |  | **5,0E-09** | *1,2E-04* | *-1,2* | **3,2E-08** | **-1,6** | *1,9E-02* | *-1,1* |
| **980** | **UDP-glucose 6-dehydrogenase** | **UGDH** | **O60701** |  | **1,1E-11** | *2,9E-03* | *-1,2* | **1,3E-06** | **-1,7** | *7,9E-02* | *1,1* |
| **429** | **Transitional endoplasmic reticulum ATPase** | **TERA** | **P55072** |  | **1,3E-03** | *2,6E-01* | *-1,2* | **1,1E-02** | **-1,8** | **1,2E-02** | **-1,6** |
| **606** | **Lamina-associated polypeptide 2, isoform alpha** | **LAP2A** | **P42166** |  | **2,6E-07** | *9,1E-06* | *-1,2* | **1,2E-05** | **-1,6** | *5,0E-01* | *-1,0* |
| **1380** | **Heterogeneous nuclear ribonucleoprotein A/B** | **ROAA** | **Q99729** |  | **1,4E-03** | *9,0E-03* | *-1,2* | **4,5E-03** | **-1,6** | *2,6E-01* | *-1,1* |
| **1980** | **3-hydroxyacyl-CoA dehydrogenase type-2** | **HCD2** | **Q99714** | **M** | **2,6E-08** | *4,4E-05* | *-1,2* | **1,1E-07** | **-1,4** | *1,1E-02* | *-1,2* |
| **577** | **Far upstream element-binding protein 2** | **FUBP2** | **Q92945** |  | **1,8E-09** | **5,9E-05** | **-1,3** | **3,9E-07** | **-1,5** | **4,3E-05** | **-1,3** |
| **1876** | **Endoplasmic reticulum resident protein 29** | **ERP29** | **P30040** |  | **1,4E-09** | **1,4E-06** | **-1,3** | **5,5E-08** | **-1,5** | *4,4E-01* | *-1,1* |
| **2100** | **ATP synthase subunit d** | **ATP5H** | **O75947** | **M** | **1,0E-06** | **6,6E-03** | **-1,3** | **4,8E-05** | **-1,6** | *5,8E-03* | *-1,3* |
| **524** | **Aconitate hydratase, mitochondrial** | **ACON** | **Q99798** | **M** | **1,4E-10** | **1,2E-07** | **-1,3** | **1,0E-07** | **-1,4** | *5,2E-04* | *-1,1* |
| **1061** | **Alpha-aminoadipic semialdehyde dehydrogenase** | **AL7A1** | **P49419** |  | **6,9E-07** | **2,0E-04** | **-1,3** | **8,0E-07** | **-1,5** | *8,5E-01* | *-1,0* |
| **288** | **2-oxoglutarate dehydrogenase** | **ODO1** | **Q02218** | **M** | **1,9E-04** | **2,6E-03** | **-1,3** | **6,7E-06** | **-1,6** | *1,0E-01* | *-1,1* |
| **584** | **Far upstream element-binding protein 2** | **FUBP2** | **Q92945** |  | **1,7E-12** | **2,4E-06** | **-1,3** | **8,9E-09** | **-1,8** | ***1,2E-03*** | ***-1,4*** |
| **1696** | **Elongation factor Ts** | **EFTS** | **P43897** | **M** | **2,3E-07** | **7,8E-05** | **-1,3** | **1,4E-04** | **-1,8** | *2,7E-02* | *-1,1* |
| **1684** | **Voltage-dependent anion-selective channel protein** | **VDAC1** | **P21796** | **M** | **1,8E-09** | **1,8E-03** | **-1,4** | **6,4E-04** | **-1,5** | *6,7E-02* | *-1,1* |
| **2278** | **Destrin** | **DEST** | **P60981** |  | **1,2E-08** | **3,3E-03** | **-1,4** | **1,8E-03** | **-1,6** | *1,4E-01* | *1,4* |
| **1115** | **Serine hydroxymethyltransferase** | **GLYM** | **P34897** | **M** | **3,0E-09** | **5,7E-06** | **-1,4** | **3,6E-07** | **-1,6** | *4,2E-01* | *-1,1* |
| **2103** | **ATP synthase subunit d** | **ATP5H** | **O75947** | **M** | **2,4E-08** | **2,7E-06** | **-1,4** | **8,2E-07** | **-1,5** | **1,4E-02** | **-1,4** |
| **1998** | **GTP-binding nuclear protein Ran** | **RAN** | **P62826** |  | **2,2E-08** | **4,3E-07** | **-1,4** | **8,1E-08** | **-1,7** | **2,1E-03** | **-1,3** |
| **1028** | **Tryptophan--tRNA ligase, cytoplasmic** | **SYWC** | **P23381** |  | **5,5E-13** | **1,3E-07** | **-1,4** | *6,0E-05* | *-1,2* | *3,8E-01* | *1,1* |
| **1669** | **Voltage-dependent anion-selective channel protein** | **VDAC2** | **P45880** | **M** | **6,3E-10** | **4,2E-05** | **-1,4** | **3,0E-07** | **-1,6** | **1,0E-05** | **-1,5** |
| **580** | **Far upstream element-binding protein 2** | **FUBP2** | **Q92945** |  | **1,0E-09** | **3,4E-04** | **-1,4** | **5,4E-05** | **-1,5** | **9,0E-04** | **-1,3** |
| **2046** | **Cysteine and glycine-rich protein 1** | **CSRP1** | **P21291** |  | **3,7E-07** | **2,3E-05** | **-1,4** | **2,7E-06** | **-1,8** | **1,4E-04** | **-1,5** |
| **305** | **Inhibitor of nuclear factor kappa-B kinase subunit alpha** | **IKKA** | **O15111** |  | **1,9E-05** | **1,2E-05** | **-1,5** | **2,3E-06** | **-1,6** | **4,7E-04** | **-1,4** |
| **1109** | **ATP synthase subunit alpha** | **ATPA** | **P25705** | **M** | **2,3E-12** | **1,9E-07** | **-1,5** | **2,7E-08** | **-1,6** | **6,6E-03** | **-1,5** |
| **1903** | **Electron transfer flavoprotein subunit beta** | **ETFB** | **P38117** | **M** | **3,3E-07** | **1,9E-05** | **-1,5** | **1,4E-03** | **-1,5** | *2,7E-02* | *-1,1* |
| **1326** | **DnaJ homolog subfamily B member 11** | **DJB11** | **Q9UBS4** |  | **3,7E-07** | **5,1E-06** | **-1,5** | **8,4E-07** | **-1,5** | *8,4E-03* | *-1,2* |
| **1919** | **Peroxiredoxin-4** | **PRDX4** | **Q13162** |  | **8,0E-11** | **5,2E-05** | **-1,5** | **8,3E-07** | **-1,5** | *7,0E-01* | *-1,0* |
| **1350** | **Alpha-2-macroglobulin receptor-associated protein** | **AMRP** | **P30533** |  | **1,5E-08** | **9,5E-06** | **-1,5** | **3,0E-06** | **-1,7** | *1,7E-02* | *-1,2* |
| **1682** | **Voltage-dependent anion-selective channel protein** | **VDAC1** | **P21796** | **M** | **3,1E-06** | **4,7E-05** | **-1,5** | **2,2E-04** | **-1,5** | **4,0E-03** | **-1,3** |
| **2020** | **Thioredoxin-dependent peroxide reductase** | **PRDX3** | **P30048** | **M** | **7,1E-10** | **3,3E-08** | **-1,5** | **6,4E-08** | **-1,5** | **2,2E-05** | **-1,5** |
| **691** | **Stress-70 protein** | **GRP75** | **P38646** | **M** | **1,8E-13** | **7,7E-04** | **-1,5** | **1,3E-05** | **-1,6** | *3,1E-01* | *1,1* |
| **1922** | **Peroxiredoxin-6** | **PRDX6** | **P30041** |  | **3,0E-07** | **2,1E-05** | **-1,5** | **6,4E-06** | **-1,5** | *1,8E-01* | *-1,1* |
| **1113** | **ATP synthase subunit beta** | **ATPB** | **P06576** | **M** | **3,2E-11** | **9,5E-07** | **-1,5** | **4,1E-07** | **-1,5** | **2,8E-02** | **-1,3** |
| **332** | **Neutral alpha-glucosidase AB** | **GANAB** | **Q14697** |  | **6,1E-11** | **7,8E-09** | **-1,5** | **1,7E-07** | **-1,4** | *2,3E-04* | *-1,2* |
| **618** | **NADH-ubiquinone oxidoreductase 75 kDa subunit** | **NDUS1** | **P28331** | **M** | **3,6E-06** | **1,3E-06** | **-1,5** | **1,2E-04** | **-1,3** | *2,3E-01* | *-1,1* |
| **1398*** | **Galactokinase** | **GALK1** | **P51570** |  | **9,2E-07** | **3,8E-06** | **-1,5** | **2,0E-05** | **-1,4** | *1,3E-02* | *-1,1* |
| **1398*** | **Replication factor C subunit 2** | **RFC2** | **P35250** |  | **9,2E-07** | **3,8E-06** | **-1,5** | **2,0E-05** | **-1,4** | *1,3E-02* | *-1,1* |
| **1110** | **ATP synthase subunit alph** | **ATPA** | **P25705** | **M** | **3,2E-08** | **7,1E-06** | **-1,5** | **2,8E-07** | **-1,7** | **1,2E-03** | **-1,4** |
| **1215** | **Calumenin** | **CALU** | **O43852** |  | **6,0E-10** | **1,1E-09** | **-1,5** | **1,4E-08** | **-1,9** | **3,9E-04** | **-1,3** |
| **170** | **Pyruvate carboxylase** | **PYC** | **P11498** | **M** | **3,8E-10** | **8,4E-05** | **-1,5** | **2,8E-04** | **-1,7** | *3,4E-01* | *-1,1* |
| **946** | **60 kDa heat shock protein** | **CH60** | **P10809** | **M** | **1,2E-11** | **2,9E-06** | **-1,5** | **7,1E-06** | **-1,3** | *6,1E-01* | *1,1* |
| **1136** | **ATP synthase subunit beta** | **ATPB** | **P06576** | **M** | **6,7E-08** | **6,6E-09** | **-1,5** | **2,5E-06** | **-1,4** | *3,1E-02* | *-1,1* |
| **2251** | **Mesencephalic astrocyte-derived neurotrophic factor** | **MANF** | **P55145** |  | **5,6E-03** | **6,3E-04** | **-1,5** | **3,2E-03** | **-1,6** | **1,0E-02** | **-1,5** |
| **47** | **Carbamoyl-phosphate synthase** | **CPSM** | **P31327** | **M** | **1,7E-08** | **1,7E-07** | **-1,5** | **6,8E-04** | **-1,6** | *7,2E-02* | *1,1* |
| **666** | **78 kDa glucose-regulated protein** | **GRP78** | **P11021** |  | **2,3E-10** | **1,4E-05** | **-1,6** | **5,3E-05** | **-1,5** | **7,3E-04** | **-1,3** |
| **455** | **Glucosidase 2 subunit beta** | **GLU2B** | **P14314** |  | **2,6E-06** | **1,6E-04** | **-1,6** | **7,8E-04** | **-1,4** | **4,4E-03** | **-1,3** |
| **1360** | **Vimentin** | **VIME** | **P08670** |  | **2,0E-08** | **3,8E-04** | **-1,6** | **1,2E-03** | **-1,5** | **2,1E-02** | **-1,3** |
| **1771** | **B-cell lymphoma/leukemia 10** | **BCL10** | **O95999** |  | **7,1E-05** | **3,0E-03** | **-1,6** | *7,8E-02* | *-1,2* | *4,6E-01* | *-1,1* |
| **1904*** | **GTP:AMP phosphotransferase AK3** | **KAD3** | **Q9UIJ7** | **M** | **9,8E-05** | **4,0E-04** | **-1,6** | **1,7E-03** | **-1,4** | *4,2E-01* | *1,1* |
| **1904*** | **Protein NipSnap homolog 1** | **NIPS1** | **Q9BPW8** | **M** | **9,8E-05** | **4,0E-04** | **-1,6** | **1,7E-03** | **-1,4** | *4,2E-01* | *1,1* |
| **2241*** | **Protein canopy homolog 2** | **CNPY2** | **Q9Y2B0** |  | **7,7E-09** | **7,6E-07** | **-1,6** | **7,4E-06** | **-1,7** | *8,6E-02* | *-1,1* |
| **2241*** | **Trafficking protein particle complex subunit 3** | **TPPC3** | **O43617** |  | **7,7E-09** | **7,6E-07** | **-1,6** | **7,4E-06** | **-1,7** | *8,6E-02* | *-1,1* |
| **2586** | **Small integral membrane protein 12** | **SIM12** | **Q96EX1** |  | **1,3E-03** | **2,5E-04** | **-1,6** |  | *-1,9* | *4,4E-01* | *1,1* |
| **1456*** | **Alpha-enolase** | **ENOA** | **P06733** |  | **3,4E-12** | **2,4E-04** | **-1,6** | **3,9E-04** | **-1,5** | *5,9E-01* | *-1,1* |
| **1456*** | **Isocitrate dehydrogenase** | **IDH3A** | **P50213** | **M** | **3,4E-12** | **2,4E-04** | **-1,6** | **3,9E-04** | **-1,5** | *5,9E-01* | *-1,1* |
| **435** | **Ezrin** | **EZRI** | **P15311** |  | **5,9E-08** | **8,0E-05** | **-1,6** | **4,1E-03** | **-1,4** | **6,2E-03** | **-1,4** |
| **1179** | **Vimentin** | **VIME** | **P08670** |  | **5,0E-06** | **6,3E-03** | **-1,6** | **1,6E-03** | **-2,0** | **7,7E-03** | **-1,7** |
| **621** | **NADH-ubiquinone oxidoreductase 75 kDa subunit** | **NDUS1** | **P28331** | **M** | **1,3E-05** | **3,8E-04** | **-1,7** | **2,7E-05** | **-1,6** | *1,3E-01* | *-1,1* |
| **2218** | **Peptidyl-prolyl cis-trans isomerase B** | **PPIB** | **P23284** |  | **4,4E-06** | **1,3E-05** | **-1,7** | **5,4E-04** | **-1,5** | **1,9E-05** | **-1,5** |
| **292** | **2-oxoglutarate dehydrogenase** | **ODO1** | **Q02218** | **M** | **5,6E-09** | **4,8E-09** | **-1,7** | **3,4E-10** | **-2,0** | **2,5E-04** | **-1,5** |
| **314** | **Neutral alpha-glucosidase AB** | **GANAB** | **Q14697** |  | **7,2E-08** | **3,0E-05** | **-1,7** | **1,6E-05** | **-1,6** | **2,3E-04** | **-1,4** |
| **538** | **Mitochondrial inner membrane protein** | **IMMT** | **Q16891** | **M** | **1,4E-07** | **2,1E-06** | **-1,7** | **2,7E-06** | **-1,6** | *6,8E-01* | *-1,0* |
| **324** | **Neutral alpha-glucosidase AB** | **GANAB** | **Q14697** |  | **9,3E-09** | **7,3E-07** | **-1,7** | **2,8E-06** | **-1,5** | **2,3E-04** | **-1,3** |
| **998** | **Protein disulfide-isomerase A3** | **PDIA3** | **P30101** |  | **3,1E-11** | **1,8E-04** | **-1,7** | **1,3E-03** | **-1,6** | **3,6E-03** | **-1,4** |
| **1008** | **Protein disulfide-isomerase A3** | **PDIA3** | **P30101** |  | **8,3E-12** | **2,2E-05** | **-1,7** | **1,6E-04** | **-1,6** | **1,1E-03** | **-1,4** |
| **436** | **Ezrin** | **EZRI** | **P15311** |  | **6,4E-05** | **3,8E-03** | **-1,8** | **1,2E-02** | **-1,5** | **2,2E-03** | **-1,8** |
| **533** | **Mitochondrial inner membrane protein** | **IMMT** | **Q16891** | **M** | **1,2E-12** | **1,2E-06** | **-1,8** | **1,2E-07** | **-1,7** | *7,8E-01* | *1,0* |
| **390** | **Endoplasmin** | **ENPL** | **P14625** |  | **2,1E-12** | **5,3E-07** | **-1,8** | **1,2E-06** | **-1,8** | **1,2E-02** | **-1,3** |
| **1597** | **Galectin-8** | **LEG8** | **O00214** |  | **2,6E-07** | **4,1E-04** | **-1,8** | **2,5E-04** | **-2,0** | *2,1E-01* | *-1,2* |
| **971** | **Protein disulfide-isomerase** | **PDIA1** | **P07237** |  | **3,7E-11** | **5,1E-07** | **-1,8** | **1,7E-07** | **-2,0** | **2,2E-02** | **-1,3** |
| **2156** | **Ferritin light chain** | **FRIL** | **P02792** |  | **1,8E-12** | **1,4E-04** | **-1,8** | **3,1E-05** | **-2,5** | *1,1E-01* | *1,1* |
| **178** | **ATP-citrate synthase** | **ACLY** | **P53396** |  | **4,3E-04** | **4,7E-05** | **-1,9** | **1,4E-05** | **-2,2** | **1,6E-04** | **-1,9** |
| **653** | **78 kDa glucose-regulated protein** | **GRP78** | **P11021** |  | **2,1E-11** | **2,4E-04** | **-1,9** | *3,2E-01* | *-1,2* | *5,7E-02* | *-1,3* |
| **2414** | **UPF0556 protein C19orf10** | **CS010** | **Q969H8** |  | **4,6E-09** | **5,9E-07** | **-1,9** | **8,0E-07** | **-2,0** | **1,2E-05** | **-1,6** |
| **28** | **Carbamoyl-phosphate synthase** | **CPSM** | **P31327** | **M** | **1,7E-13** | **3,5E-05** | **-1,9** | **3,0E-03** | **-2,0** | *1,0E-02* | *1,1* |
| **1112** | **ATP synthase subunit beta** | **ATPB** | **P06576** | **M** | **9,4E-07** | **2,5E-05** | **-2,0** | **9,1E-06** | **-1,9** | **3,2E-02** | **-1,4** |
| **52** | **Carbamoyl-phosphate synthase** | **CPSM** | **P31327** | **M** | **4,0E-06** | **3,2E-07** | **-2,0** | **5,4E-03** | **-1,8** | *6,5E-01* | *1,1* |
| **1847*** | **Proteasome subunit alpha type-3** | **PSA3** | **P25788** |  | **3,3E-08** | **3,0E-06** | **-2,0** | **2,1E-06** | **-2,1** | **1,4E-06** | **-2,0** |
| **1847*** | **Tumor protein D54** | **TPD54** | **O43399** |  | **3,3E-08** | **3,0E-06** | **-2,0** | **2,1E-06** | **-2,1** | **1,4E-06** | **-2,0** |
| **49** | **Carbamoyl-phosphate synthase** | **CPSM** | **P31327** | **M** | **2,0E-09** | **1,9E-09** | **-2,1** | **1,6E-03** | **-2,0** | *6,3E-02* | *1,1* |
| **2318** | **Alpha-synuclein** | **SYUA** | **P37840** |  | **3,3E-14** | **2,6E-05** | **-2,1** | **6,7E-08** | **-3,4** | *4,9E-01* | *-1,1* |
| **51** | **Carbamoyl-phosphate synthase** | **CPSM** | **P31327** | **M** | **6,7E-16** | **1,4E-05** | **-2,2** | **3,0E-03** | **-2,2** | *2,7E-02* | *1,1* |
| **1024** | **Protein disulfide-isomerase** | **PDIA1** | **P07237** |  | **2,1E-10** | **3,3E-06** | **-2,2** | **2,6E-06** | **-2,1** | *6,9E-02* | *-1,3* |
| **1134*** | **NF-kappa-B essential modulator** | **NEMO** | **Q9Y6K9** |  | **1,6E-06** | **2,8E-03** | **-2,2** | **6,3E-03** | **-1,9** | *3,8E-01* | *-1,2* |
| **1134*** | **Na(+)/H(+) exchange regulatory cofactor NHE-RF1** | **NHRF1** | **O14745** |  | **1,6E-06** | **2,8E-03** | **-2,2** | **6,3E-03** | **-1,9** | *3,8E-01* | *-1,2* |
| **175** | **Elongation factor 2** | **EF2** | **P13639** |  | **6,1E-07** | **6,0E-06** | **-2,2** | **8,0E-07** | **-2,5** | **1,9E-05** | **-2,4** |
| **64** | **Carbamoyl-phosphate synthase** | **CPSM** | **P31327** | **M** | **1,6E-14** | **1,3E-06** | **-2,3** | **3,3E-03** | **-2,3** | *6,2E-02* | *1,1* |
| **2015** | **Heterogeneous nuclear ribonucleoprotein H** | **HNRH1** | **P31943** |  | **1,0E-09** | **9,8E-06** | **-2,3** | **3,3E-05** | **-2,0** | *6,0E-02* | *-1,3* |
| **50** | **Carbamoyl-phosphate synthase** | **CPSM** | **P31327** | **M** | **9,2E-13** | **1,6E-07** | **-2,4** | **1,9E-03** | **-2,3** | *1,0E-01* | *1,1* |
| **1820** | **Galectin-3** | **LEG3** | **P17931** |  | **4,8E-14** | **8,5E-06** | **-2,4** | **1,0E-03** | **-1,8** | **5,6E-03** | **-1,8** |
| **1250** | **Reticulocalbin-1** | **RCN1** | **Q15293** |  | **2,5E-12** | **2,0E-05** | **-2,8** | **2,2E-05** | **-2,6** | *4,0E-01* | *-1,2* |
| **1318** | **Reticulocalbin-1** | **RCN1** | **Q15293** |  | **1,4E-10** | **9,9E-07** | **-4,2** | **1,7E-06** | **-4,0** | **1,3E-02** | **-1,7** |

The full protein names are from the UniProt database. Accession number are from UniProt (Acc._HUMAN) and SwissProt data bases. The one-way analysis of variance (ANOVA) test, followed by a Tukey’s multiple comparison test, was used to determine protein spots significantly different between analyses. *p*-values were calculated across pairwise comparisons (clones N vs H, R vs H and V vs H) and considered significant when <0.05. Proteins were ordered following the fold changes in the N vs H comparison. * Two identifications for the same spot. Bold values, fold change statistically valid (*p*< 0.05) and ≥1.3. Italic values, fold change not statistically valid (*p* > 0.05) or ≤1.3. § Proteins reported to present a mitochondrial localization (UniProt annotation) are indicated by M.
