# Supplementary material for: The mitochondrially-localized nucleoside diphosphate kinase D (NME4) is a novel metastasis suppressor
Source: BMC Biol. 2021 Oct 21;19:228. doi: 10.1186/s12915-021-01155-5 (PMC8529772; doi:10.1186/s12915-021-01155-5)
Supplement: Supplementary file 20 — Additional file 20: Table S3. Characteristics of the 526 human breast tumor cohort. [file 12915_2021_1155_MOESM20_ESM.doc]

Table S3: Characteristics of the 526 breast tumors

|  | Number of patients (%) | Number with metastases (%) | p-valuea |
| --- | --- | --- | --- |
|  |  |  |  |
| *Total* | 526 (100) | 209 (39.7) |  |
|  |  |  |  |
| *Age*  ≤50  >50 | 125 (23.8)  401 (76.2) | 52 (41.6)  157 (39.2) | 0.71 (NS) |
| *SBR histological grade* b,c  I  II  III | 60 (11.7)  241 (47.2)  210 (41.1) | 12 (20.0)  100 (41.5)  93 (44.3) | **0.0012** |
| *Lymph node status* d  0  1-3  >3 | 160 (30.7)  248 (47.6)  113 (21.7) | 48 (30.0)  87 (35.1)  72 (63.7) | **<0.0001** |
| *Macroscopic tumor size e*  ≤25mm  >25mm | 248 (48.1)  268 (51.9) | 76 (30.6)  132 (49.3) | **<0.0001** |
| *ERα status*  Negative  Positive | 181 (34.4)  345 (65.6) | 76 (42.0)  133 (38.6) | 0.14 (NS) |
| *PR status*  Negative  Positive | 254 (48.3)  272 (51.7) | 109 (42.9)  100 (36.8) | **0.032** |
| *ERBB2 status*  Negative  Positive | 395 (75.1)  131 (24.9) | 152 (38.5)  57 (43.5) | 0.39 (NS) |
| *Molecular subtypes*  HR- ERBB2-  HR- ERBB2+  HR+ ERBB2-  HR+ ERBB2+ | 101 (19.2)  73 (13.9)  294 (55.9)  58 (11.0) | 38 (37.6)  36 (49.3)  114 (38.8)  21 (36.2) | 0.11 (NS) |
| *PIK3CA mutation status f*  wild type  mutated | 354 (68.1)  166 (31.9) | 145 (41.0)  61 (36.7) | 0.22 (NS) |

a Log-rank test (521 samples with MFS>6 months). NS: not significant

b Scarff Bloom Richardson classification

c Information available for 511 patients

d Information available for 521 patients

e Information available for 516 patients

f Information available for 520 patients
