# Supplementary material for: The mitochondrially-localized nucleoside diphosphate kinase D (NME4) is a novel metastasis suppressor
Source: BMC Biol. 2021 Oct 21;19:228. doi: 10.1186/s12915-021-01155-5 (PMC8529772; doi:10.1186/s12915-021-01155-5)
Supplement: Supplementary file 23 — Additional file 23: Table S4. Association between NME4 and EMT and tumor invasion marker expression in breast tumors. The relationship between NME4 expression and several key players of EMT and tumor invasion was studied in human breast tumors from the TCGA database. [file 12915_2021_1155_MOESM23_ESM.docx]

Table S4: Association between *NME4* and EMT and tumor invasion marker expression in breast tumors

| Correlated Gene | Description | Spearman’s Correlation | p-value |
| --- | --- | --- | --- |
| **EMT** |  |  |  |
| *KRT18* | CK18 | 0.392 | 1.17.10^-41^ |
| *KRT8* | CK8 | 0.336 | 2.13.10^-30^ |
| *VIM* | Vimentin | -0.125 | 3.048.10^-5^ |
| *CDH2* | N-cadherin | -0.192 | 1.40.10^-10^ |
| *SNAI1* | Snail | -0.0689 | 0.0223 |
| *SNAI2* | Slug | -0.189 | 2.69.10^-10^ |
| *ZEB1* | ZEB1 | -0.263 | 6.93.10^-19^ |
| *ZEB2* | ZEB2 | -0.382 | 1.62.10^-39^ |
| *CTNNB1* | β-catenin | -0.362 | 2.20.10^-35^ |
| *JUP* | plakoglobin | 0.175 | 5.52.10^-9^ |
| *TJP3* | ZO-3 | 0.311 | 4.40.10^-26^ |
| *CLDN3* | Claudin 3 | 0.316 | 5.70.10^-27^ |
| **INVASION** |  |  |  |
| *MMP7* | MMP7 | -0.210 | 1.98.10^-12^ |
| *ADAM17* | ADAM17 | -0.495 | 4.00.10^-69^ |
| *CTSB* | Cathepsin B | -0.104 | 5.405.10^-4^ |
| *CDC42* | Cdc42 | -0.100 | 8.866.10^-4^ |
| *RHOA* | RhoA | -0.0573 | 0.05 |
| *ROCK1* | ROCK1 | -0.494 | 7.99.10^-69^ |
| *ROCK2* | ROCK2 | -0.535 | 1.79.10^-82^ |
| *LIMK2* | LIMK2 | -0.215 | 6.41.10^-13^ |
| *CFL2* | Cofilin 2 | -0.307 | 1.68.10^-25^ |
| *MYO5A* | Myosin Va | -0.477 | 1.13.10^-63^ |
