# Supplementary material for: The mitochondrially-localized nucleoside diphosphate kinase D (NME4) is a novel metastasis suppressor
Source: BMC Biol. 2021 Oct 21;19:228. doi: 10.1186/s12915-021-01155-5 (PMC8529772; doi:10.1186/s12915-021-01155-5)
Supplement: Supplementary file 25 — Additional file 25: Table S5. Association between NME4, EMT and tumor invasion marker expression in cervix tumors. The relationship between NME4 expression and several key players of EMT and tumor invasion was studied in human cervix tumors from the TCGA database. [file 12915_2021_1155_MOESM25_ESM.docx]

Table S5: Association between *NME4* and EMT and tumor invasion marker expression in cervix tumors

| Correlated Gene | Description | Spearman’s Correlation | p-value |
| --- | --- | --- | --- |
| **EMT** |  |  |  |
| *KRT18* | CK18 | 0.471 | 1.26.10^-17^ |
| *KRT8* | CK8 | 0.398 | 1.32.10^-12^ |
| *SNAI2* | Slug | -0.199 | 5.892.10^-4^ |
| *ZEB2* | ZEB2 | -0.115 | 0.0483 |
| *CLDN3* | Claudin 3 | 0.241 | 2.949.10^-5^ |
| **INVASION** |  |  |  |
| *MMP14* | MT1-MMP | -0.232 | 5.839.10^-5^ |
| *ADAM20* | ADAM20 | -0.187 | 1.247.10^-3^ |
| *CTSS* | Cathepsin S | -0.149 | 0.0107 |
| *CDC42* | Cdc42 | -0.322 | 1.64.10^-8^ |
| *RAC1* | Rac1 | -0.366 | 9.72.10^-11^ |
| *LIMK2* | LIMK2 | -0.165 | 4.597.10^-3^ |
| *MYO5A* | Myosin Va | -0.244 | 2.363.10^-5^ |
| *SH3PXD2A* | TKS5 | -0.306 | 8.92.10^-8^ |
| *NCK1* | NCK1 | -0.364 | 1.25.10^-10^ |
| *NCK2* | NCK2 | -0.271 | 2.520.10^-6^ |
| *PTK2B* | Pyk2 | -0.259 | 7.003.10^-6^ |
