# Supplementary material for: The mitochondrially-localized nucleoside diphosphate kinase D (NME4) is a novel metastasis suppressor
Source: BMC Biol. 2021 Oct 21;19:228. doi: 10.1186/s12915-021-01155-5 (PMC8529772; doi:10.1186/s12915-021-01155-5)

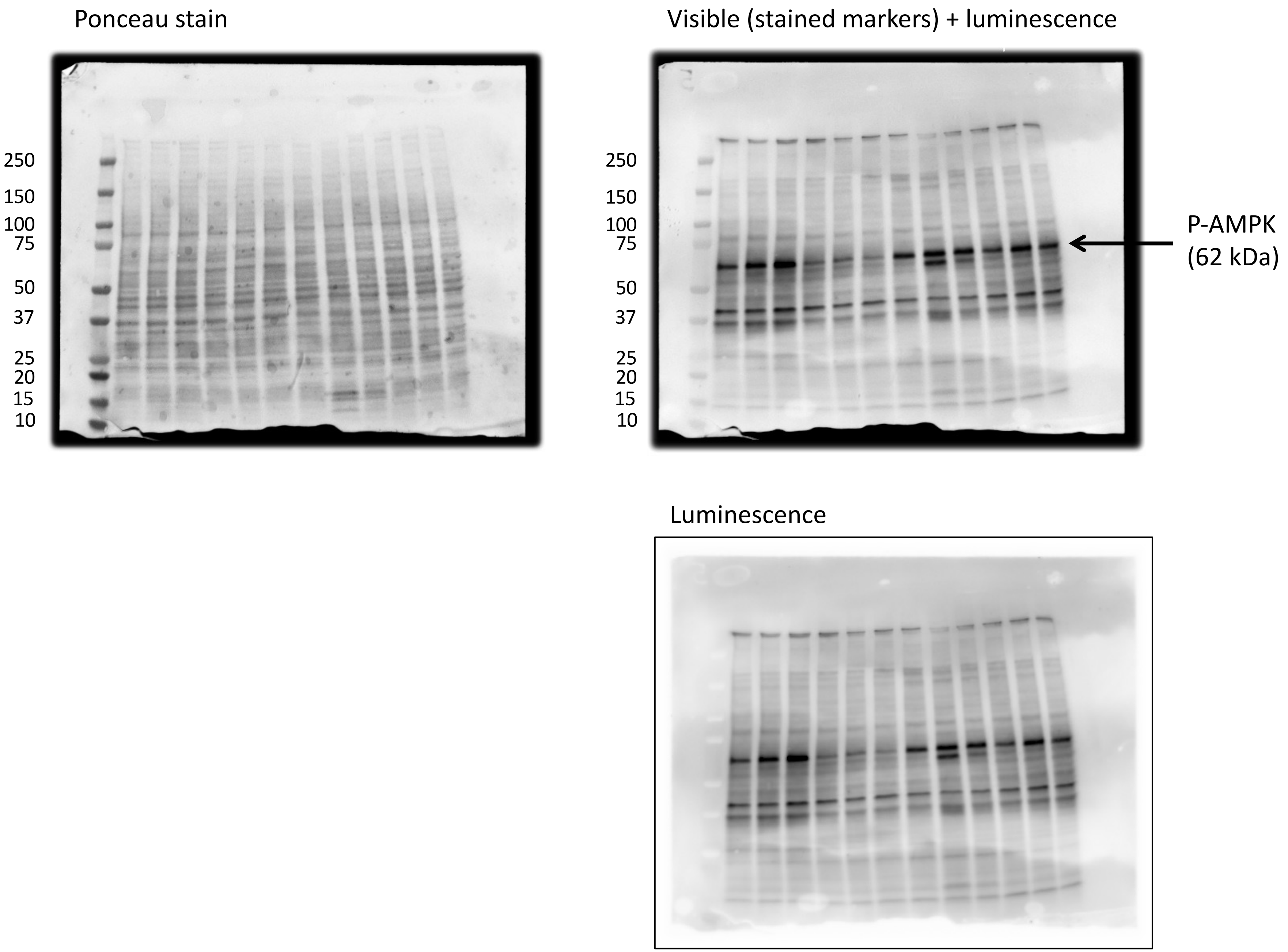
Ponceau stain
Visible (stained markers) + luminescence
Luminescence
250
150
100
75
50
37
25
20
15
10
P-AMPK
(62 kDa)

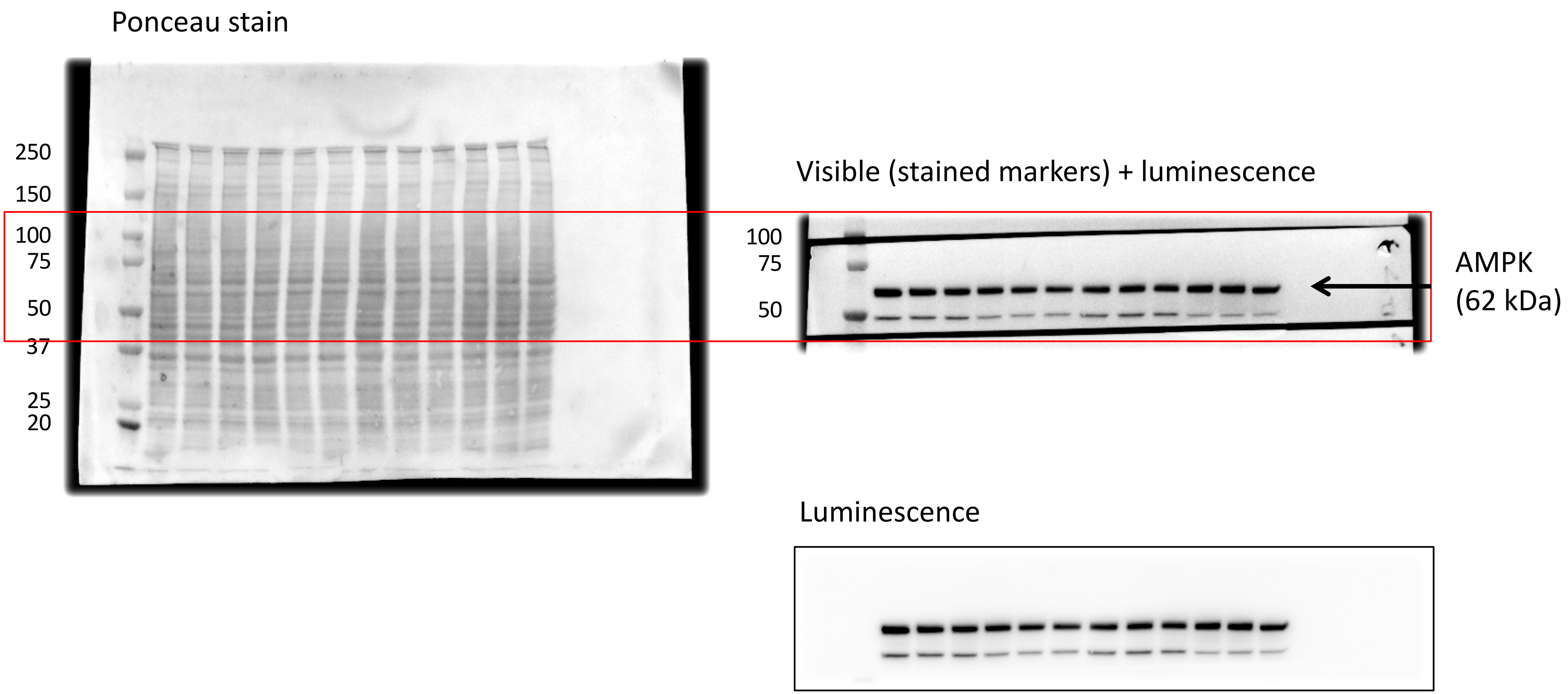

Ponceau stain
250
150
100
75
50
37
25
20
Visible (stained markers) + luminescence
100
75
50
AMPK
(62 kDa)
Luminescence

Ponceau stain

250
150
100
75
50
37
25
20

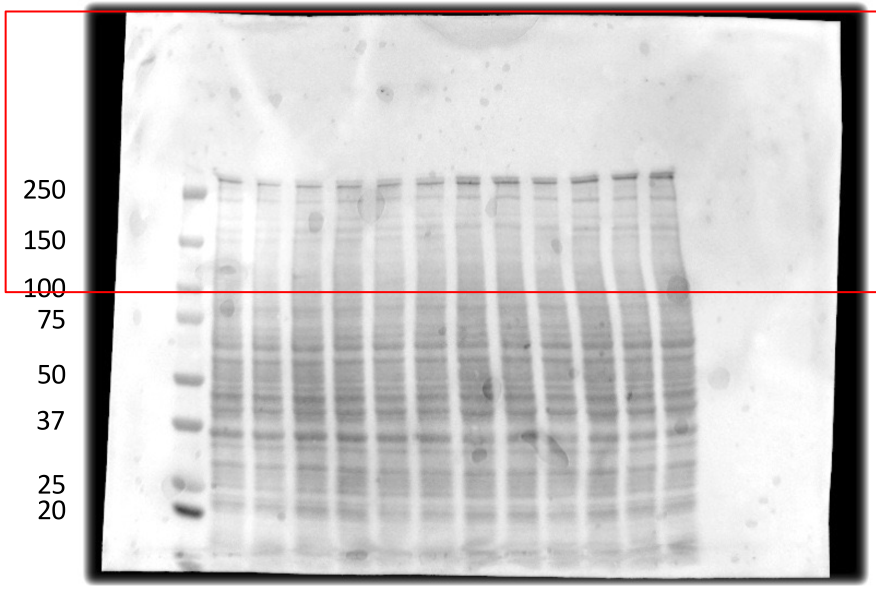

Visible (stained markers) + luminescence

250
150
100

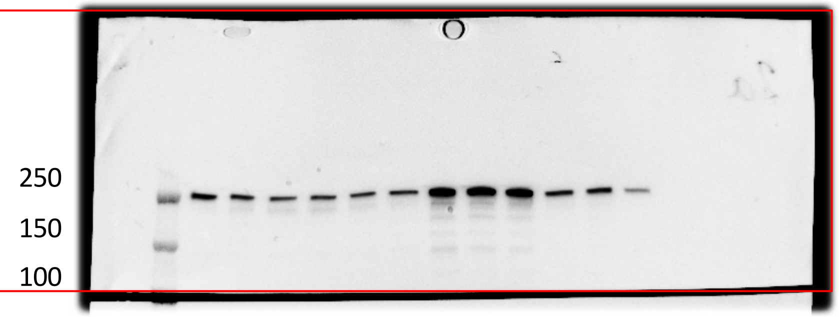

P-ACC
(266 kDa)

Luminescence

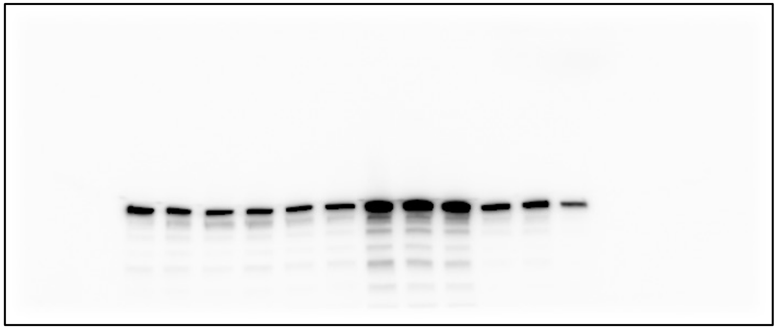

Ponceau stain

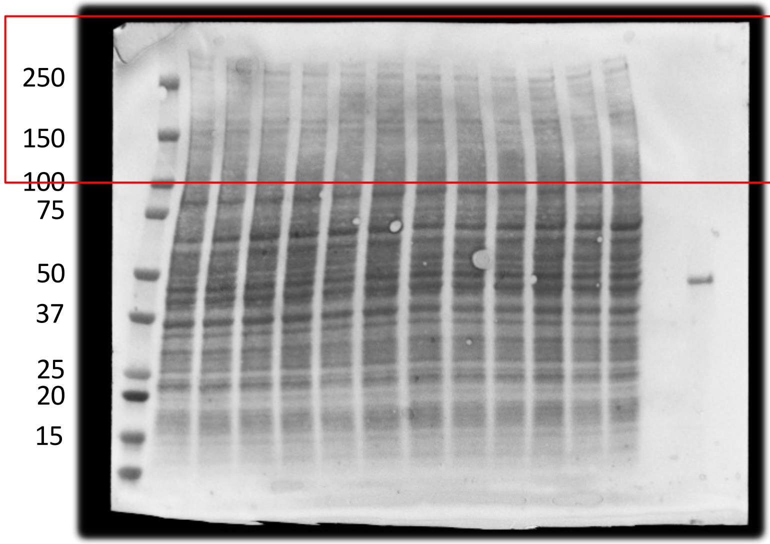

Visible (stained markers) + luminescence

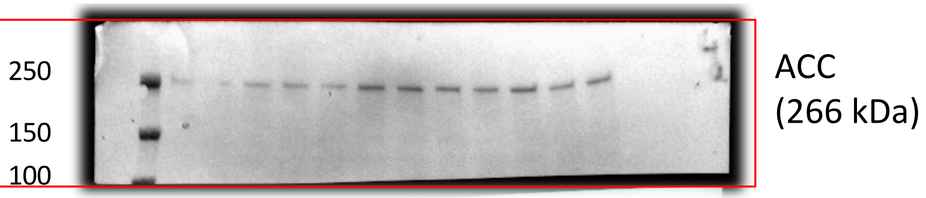

Luminescence

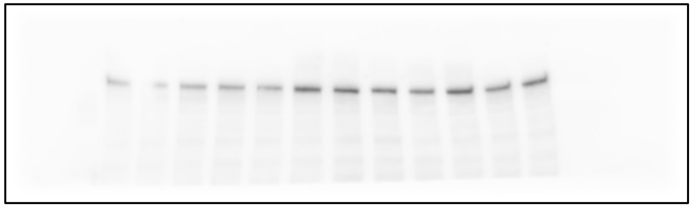

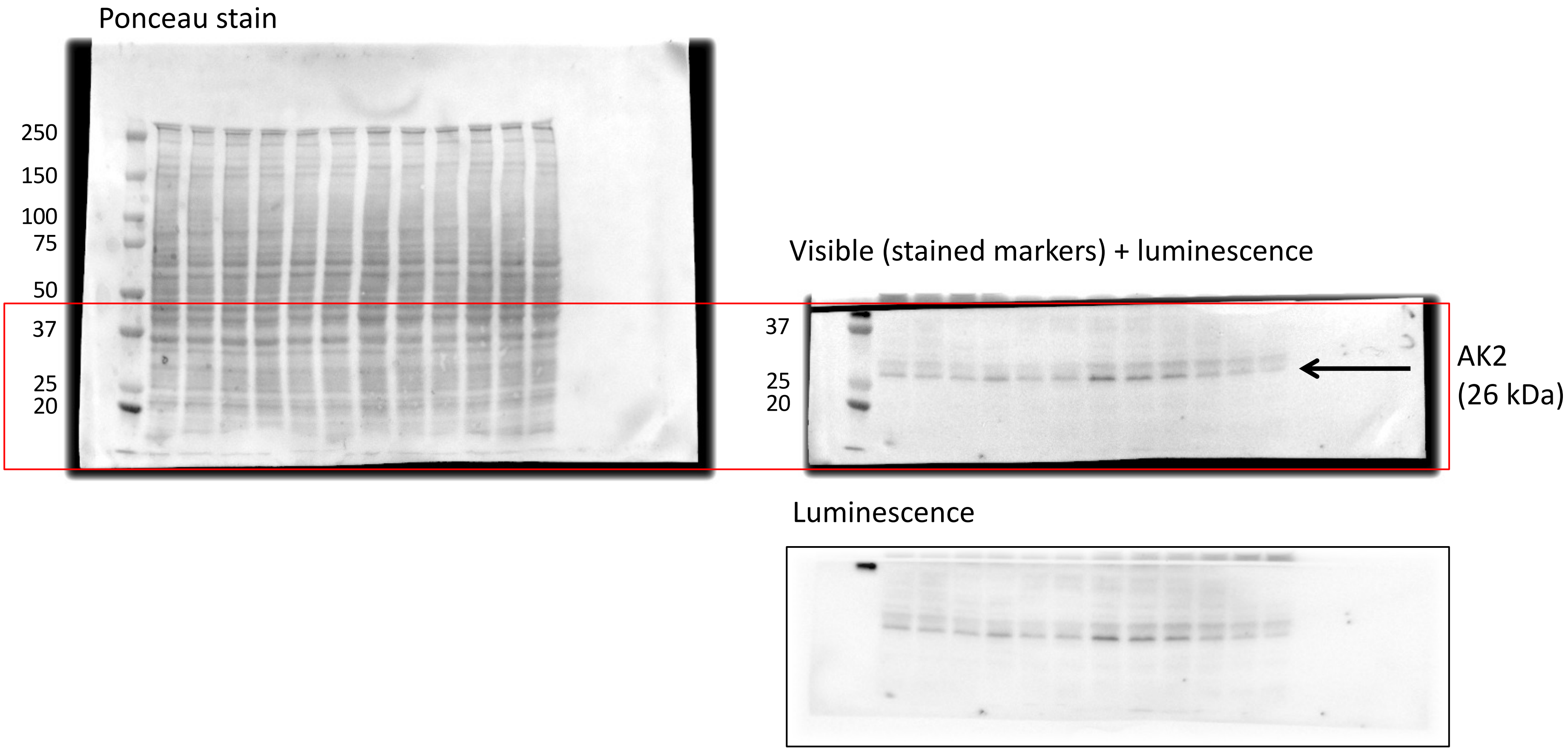
Ponceau stain
250
150
100
75
50
37
25
20
Visible (stained markers) + luminescence
37
25
20
AK2
(26 kDa)
Luminescence

Ponceau stain

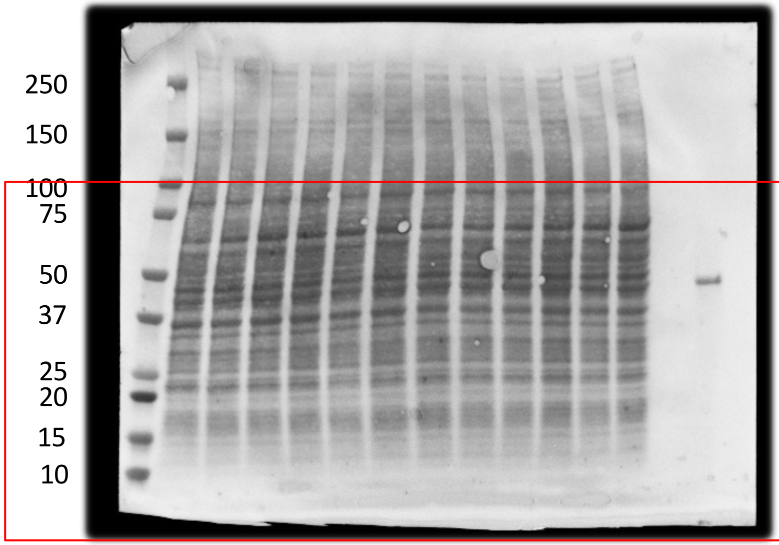

Visible (stained markers) + luminescence

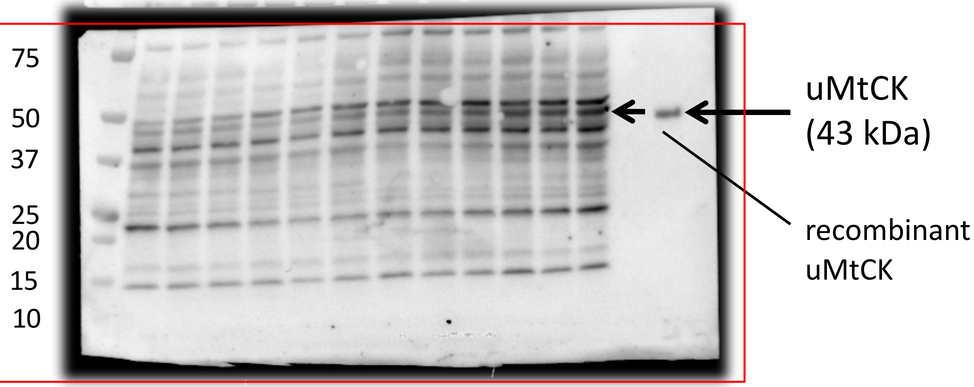

Luminescence

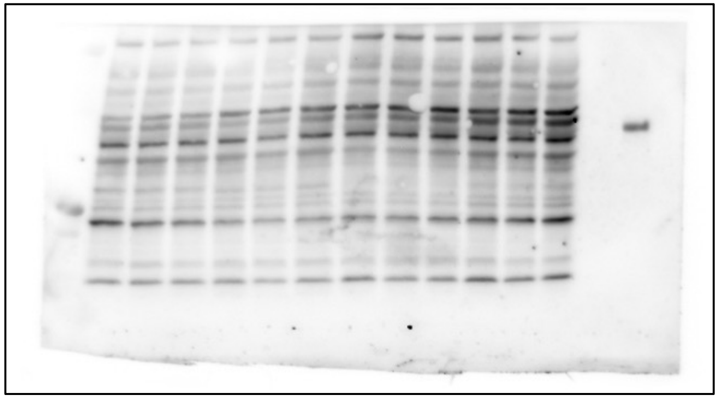

Ponceau stain

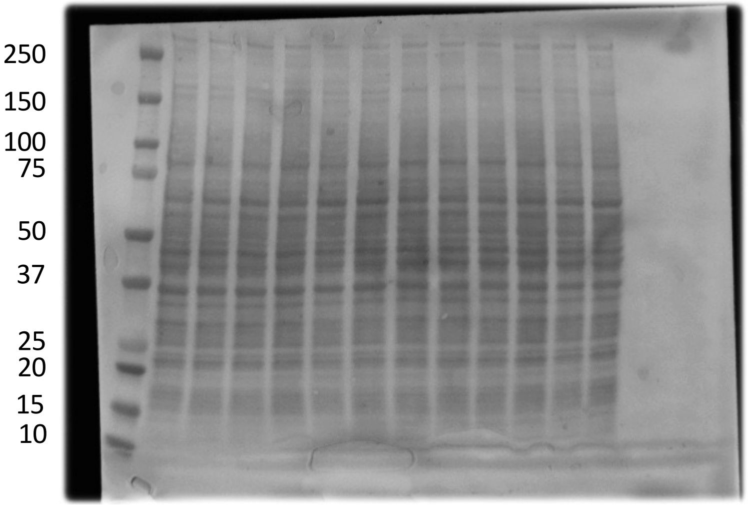

Visible (stained markers) + luminescence

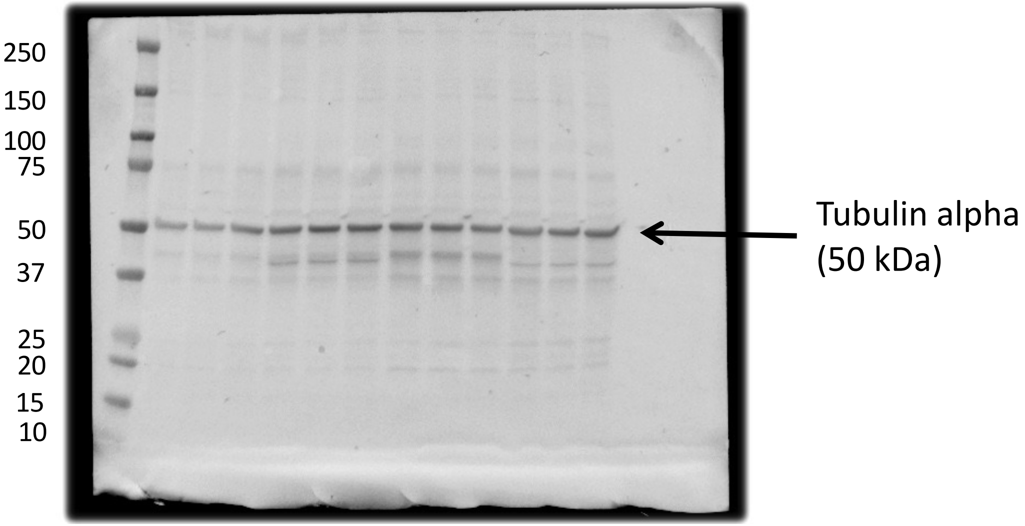

Luminescence

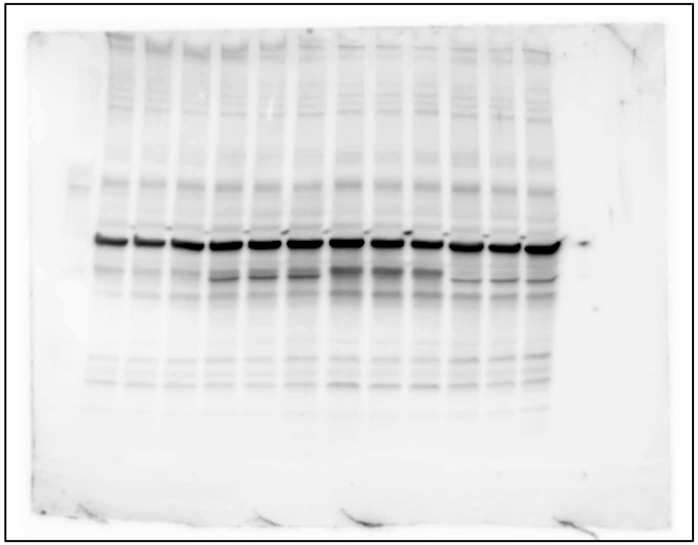

Supplement: Supplementary file 26 — Additional file 26. Images of the full immunoblots. [file 12915_2021_1155_MOESM26_ESM.zip › 26/Figure 6.pdf]
